# Safirinium Fluorescent “Click” Molecular Probes: Synthesis, CuAAC Reactions, and Microscopic Imaging

**DOI:** 10.3390/molecules30030731

**Published:** 2025-02-06

**Authors:** Patryk Kasza, Przemysław W. Szafrański, Joanna Fedorowicz, Faustyna Krzysztofiak, Krzysztof Pociecha, Katarzyna Wójcik-Pszczoła, Paulina Koczurkiewicz-Adamczyk, Mariusz Kępczynski, Jarosław Sączewski, Paweł Zajdel, Marek Cegła

**Affiliations:** 1 Faculty of Pharmacy, Jagiellonian University Medical College, Medyczna 9, 30-688 Kraków, Poland; patrykkasza@gmail.com (P.K.); k.pociecha@uj.edu.pl (K.P.); katarzynaanna.wojcik@uj.edu.pl (K.W.-P.); paulina.koczurkiewicz@uj.edu.pl (P.K.-A.); pawel.zajdel@uj.edu.pl (P.Z.); marek.cegla@uj.edu.pl (M.C.); 2 Faculty of Pharmacy, Medical University of Gdańsk, Al. Gen. J. Hallera 107, 80-416 Gdańsk, Poland; jfedorowicz@gumed.edu.pl (J.F.); js@gumed.edu.pl (J.S.); 3 Faculty of Chemistry, Jagiellonian University, Gronostajowa 2, 30-387 Kraków, Poland; m.kepczynski@uj.edu.pl

**Keywords:** fluorescent probe, click chemistry, azide, alkyne, Safirinium, CuAAC

## Abstract

Fluorescent labeling utilizing Cu(I)-catalyzed azide–alkyne cycloaddition reactions (CuAAC) is among the leading applications of the “click” chemistry strategy. Fluorescent probes for this approach can be constructed by linking an azide or alkyne group to a fluorophore, such as the recently developed Safirinium derivatives. These compounds are water-soluble, highly fluorescent heterocycles based on 1,2,4-triazolium, with significant potential for various labeling applications, although they have not yet been converted to azide or alkyne probes. Herein, we report the synthesis of Safirinium-based azide and alkyne functionalized molecular probes for “click” chemistry labeling. We also describe their CuAAC reactions with model compounds, including a lipid mimetic long-chain azide, an azido sugar derivative, and azidothymidine, as well as two model alkynes. We demonstrate that the Safirinium-based probes and their derivatives are chemically stable, suitable for fluorescent microscopy observations, and safe to use. Most of these probes show no toxic effects on CHO-K1 and NIH-3T3 cells.

## 1. Introduction

Fluorescent labelling and derivatization are often used for the analysis of biologically active compounds in biological matrices [1,2]. In biochemical and ADME studies, labelling can help determine drug distribution and their fate in the organism [3,4]. On the other hand, tracking and visualization of sugars, lipids, nucleic acids, or proteins are vital for understanding biological processes and mechanisms, as well as developing and improving pharmacological therapies [5,6].

The labelling reactions should be efficient, selective, and compatible with an aqueous environment [7,8]. These requirements are met by the copper-catalysed azide–alkyne cycloaddition (CuAAC) reaction, a flagship reaction of the click chemistry approach [9,10]. This strategy focuses on the use of efficient and easy-to-perform reactions, and is widely used in pharmaceutical sciences and analytical chemistry [11,12,13]. Recent developments in click chemistry range from simple tools [14,15] to artificial enzymes [16,17]. The click chemistry approach is also a perfect fit for fluorescent labeling [18,19] and creating theranostics [20]. The labeling application created a great need for fluorescent probes containing azide and alkyne fragments, suitable for the CuAAC-based labelling, particularly in various biological environments [21,22].

**Figure 1:**
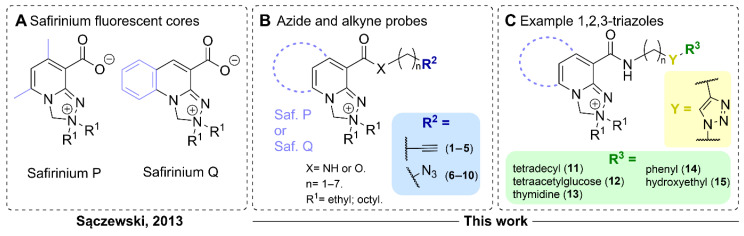
Structures of Safirinium P and Q dyes and their “click”—oriented modifications described here: (**A**) structures of Safirinium P and Q zwitterions [23]; (**B**) a general structure of Safirinium-based alkyne probes **1**–**5** and azide probes **6**–**10** obtained herein; and (**C**) a general structure, for example, 1,2,3-triazole derivatives **11**–**15** of the Safirinium-based azide and alkyne probes.

The recently developed Safirinium dyes are notable among the fluorophores that have yet to be converted into azide and alkyne probes (Figure 1A). These dyes possess a polycyclic ring system that includes a 1,2,4-triazole ring and a quaternary ammonium cation, both of which contribute to their improved aqueous solubility. The most important classes of Safirinium fluorophores comprise pyridine (Safirinium P, Figure 1A) or quinoline derivatives (Safirinium Q, Figure 1A) [23]. These dyes are characterized with high quantum yields, sizeable Stokes shifts, low molecular weight and stability in solvents [24]. Initially they were used in sensitive and selective detection of formaldehyde and secondary aliphatic amines [23]. Safirinium dyes are used as ionization tags in mass spectrometry for proteomic analysis [25,26]. Moreover, they are used for cytotoxic [27] and antibacterial [28,29,30] agent construction or staining bacteria [31]. The carboxyl group in Safirinium is a ready-to-use attachment point for labeling, most often accomplished using non-selective NHS chemistry [31].

Herein, we report on the synthesis of alkyne- and azide-functionalized fluorescent molecular probes (**1**–**10**, Figure 1B) based on Safirinium fluorophores, for fluorescent labelling and derivatization using azide–alkyne cycloaddition reactions. We used the developed probes to synthesize 1,2,3-triazole derivatives (**11**–**15**) using CuAAC reactions (Figure 1C). To obtain these derivatives, we utilized model non-fluorescent azide/alkyne counterparts, including a long-chain lipid mimetic azide, an azido sugar derivative, azidothymidine, and two simple alkynes. We studied UV–VIS absorption and fluorescence characteristics for selected probes and triazole derivatives. Finally, the in vitro cytotoxicity and cellular fluorescent microscopic imaging properties for selected probes and triazole derivatives have been assessed.

## 2. Results and Discussion

### 2.1. Synthesis of Safirinium-Based Azide and Alkyne—Functionalized Fluorescent Probes

**Figure 2:**
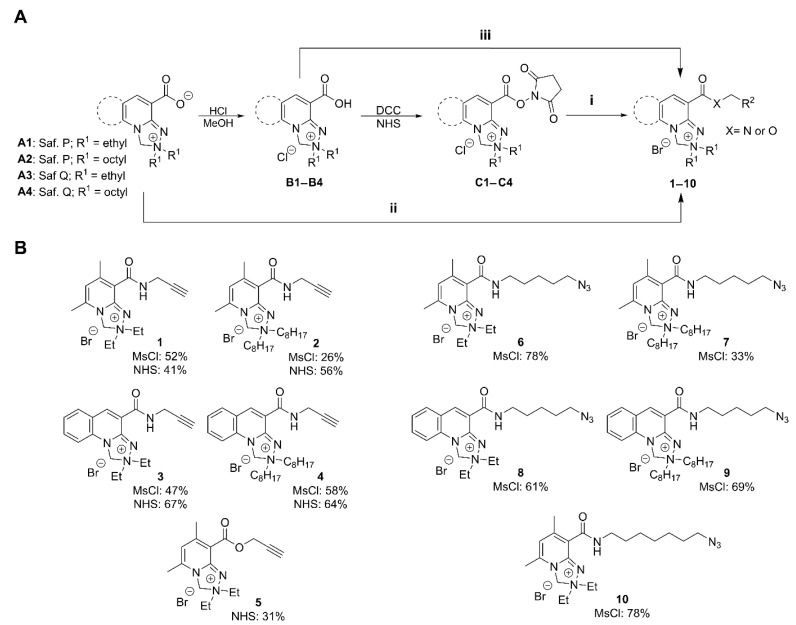
(**A**) The synthesis of Safirinium-based alkyne (**1**–**5**) and azide (**6**–**10**) fluorescent probes. Experimental conditions for esterification/amidation reactions: **i.** (NHS ester amidation/esterification): 1 eq. K_2_CO_3_, 1.5 eq. amine or alcohol, dichloromethane, RT overnight; **ii.** (MsCl amidation): 1 eq. K_2_CO_3_, 1–1.2 eq. MsCl, dichloromethane, RT 1h, then 1–1.5 eq amine, RT, overnight; **iii.** (CDI amidation): 1.25 eq. CDI, dichloromethane, RT 1h, then 1.5 equiv. amine, RT, overnight; and (**B**) the structures of Safirinium-based alkyne probes **1**–**5** and azide probes **6**–**10** with NHS (i) and/or MsCl (ii) amidation yields. Abbreviations: Saf. = Safirinium.

The key step for the synthesis of Safirinium-based azide- and alkyne-functionalized molecular probes was linking the fluorophore with azide or alkyne fragments using amide or ester bonds (Figure 2A). To find an efficient, convenient, and scalable protocol for azide/alkyne probe synthesis, we investigated three modes of carboxyl group activation: NHS esters (C1–C4), methanesulfonyl chloride (MsCl) activation of zwitterions **A1**–**A4** and, additionally, carbonyldiimidazole (CDI) activation of Safirinium carboxylic acid forms (**B1**–**B4**). As the amine counterparts in amide synthesis, we selected commercially available propargylamine (alkyne probes **1**–**4**) and easily synthesized azidoalkyl amines (azide probes **6**–**10**). For the esterification reaction (alkyne probe **5**), we used propargyl alcohol.

The amide formation reactions using NHS esters **C1**–**C4** (synthesis of alkyne probes **1**–**4**) were very efficient and, in most cases, proceeded overnight with complete conversion of the active ester. In some cases, it was possible to obtain pure products after filtering off precipitates (NHS and the inorganic base) and evaporating the solvent, without chromatographic purification. However, this was only possible for high-purity NHS ester substrates. If the NHS ester contained some free acid Safirinium, which is its decomposition product, chromatography after amide formation was necessary.

The amide formation reaction with MsCl (synthesis of probes **1**–**4** and **6**–**10**) was completed directly with zwitterionic Safirinium P (**A1**–**A2**) and Safirinium Q (**A3**–**A4**), which saved the time and effort needed to prepare and purify the NHS esters. It enabled rapid substrate conversion with satisfactory-to-good reaction yields (26–78%, Figure 2B). The better availability of the Safirinium zwitterions **A1**–**A4** made us choose this protocol for the syntheses of azide probes **6**–**10**. However, in all cases, this protocol required chromatographic purification, and the most problematic point was the removal of the methanesulfonamide byproducts, difficult to observe using standard TLC visualization methods.

We also carried out some test reactions with carbonyldiimidazole (CDI) activation of Safirinium carboxylic acid form (**B1**). The CDI amidation with propargylamine was completed in nearly quantitative yields with a short reaction time (up to 2 h). Unfortunately, imidazole formed during the reaction was difficult to separate from the amide product as it coeluted in various chromatographic systems tested.

To sum up, we found that azide- and alkyne-functionalized probes based on Safirinium can be efficiently synthesized using NHS esters **C1**–**C4**, like in earlier studies, or with the MsCl activation of the zwitterions **A1**–**A4**. The scale and yield of the developed syntheses is comparable to or better than that of some known fluorescent probes. For example, a cyanine-3 alkyne derivative for the ratiometric analysis of azidothymidine, used to study azidothymidine incorporation into DNA, was synthesized using EDC amidation (13 mg scale, 28% yield) [32]. In their studies on cyclooctyne probes for copper-free click chemistry, Grost and Berg described high-yield syntheses of a cyclooctyne BODIPY derivative, as well as 5-TAMRA azide and an azide derivative of BODIPY, using p-nitrophenyl carbonate or NHS ester chemistry; however, these efficient reactions were conducted on a small scale (3–15 mg) [33]. In a study on an efficient BODIPY-based light harvesting system built with click chemistry, large-scale (1 g) syntheses of BODIPY azide and alkyne derivatives were described, using BODIPY-related carbonyl chemistry, with limited yields (19–34%) [34].

### 2.2. Chromatographic Purification of the Safirinium-Based Probes

The ionic nature of Safirinium probes presents some challenges for their chromatographic purification. In prior studies involving Safirinium and its probes and fluorescent conjugates were typically purified using reversed-phase column chromatography or preparative RP-HPLC. While these methods are often effective for polar and ionic compounds and are becoming increasingly prevalent, they impose limitations on the reaction scale and require the use of RP-18 stationary phases. To address these challenges, we developed two procedures for purifying Safirinium derivatives using normal-phase chromatography on silica gel. The first one involves saturating a silica gel column with sodium bromide, which facilitates the elution of ionic substances. This approach is based on the work of Bluhm et al. [35] and Urban et al. [36], who successfully purified a series of pyridinium and imidazolium derivatives using similar techniques. The second one is a slight modification of the first, using a 3% methanolic NaBr solution as an eluent component.

### 2.3. CuAAC Reactions with Safirinium-Based Alkyne- and Azide-Functionalized Probes

**Figure 3:**
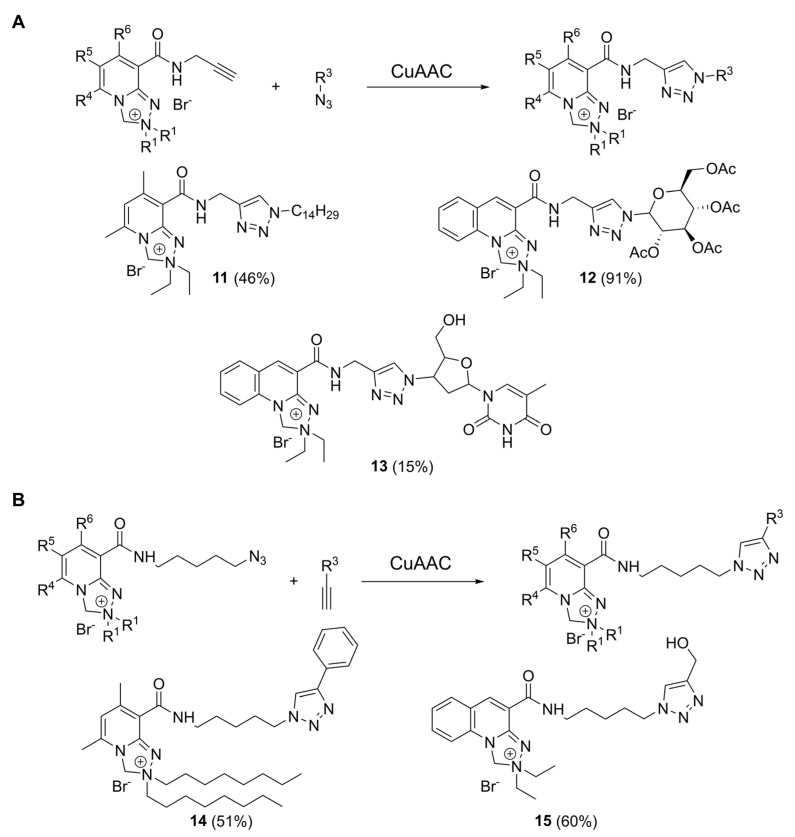
(**A**) CuAAC reactions with Safirinium-based alkyne probes using tetradecyl azide (triazole **11**), azide-modified tetraacetyl glucose (triazole **12**), and zidovudine (triazole **13**). (**B**) CuAAC reactions with Safirinium-based azide probes with phenylacetylene (triazole **14**) and propargyl alcohol (triazole **15**). Experimental CuAAC conditions: 10 mol% CuSO_4_, 20 mol% AMTC ligand, 10 mol% Na ascorbate, 30 °C, overnight, solvent: for alkyne probes—9:1 *v*/*v* dichloromethane: water; for azide probes: 1:2 *v*/*v* dichloromethane: water (**14, 15**) or 9:1 *v*/*v* water: methanol (**15**).

CuAAC reactions were carried out using a protocol we previously elaborated for azidothymidine labeling [18,37]. We reacted alkyne probes **1** and **3** with various azide counterparts (Figure 3A): 1-azidotetradecane (triazole **11**), azidotetraacetyl glucose (triazole **12**), and azidothymidine (triazole **13**), as well as azide probes (Figure 3B) with phenylacetylene (triazole **14**) and propargyl alcohol (triazole **15**). This method utilized a dichloromethane–water mixture and AMTC as the Cu-binding ligand [38], which enhanced the solubility of Cu(I) species and improved reaction outcomes compared to ligand-free conditions [37].

The synthesis of triazole **11** on a 30 mg scale proceeded in moderate yields (46%). In contrast, the reaction with a glucose derivative (triazole **12**), conducted on a smaller scale (9 mg), resulted in a good yield, with purification limited to filtering through a silica pad to remove cooper. However, the reaction involving AZT, the most polar azide substrate, resulted in a low yield, probably due to the limited substrate solubility in dichloromethane or difficulties encountered during the purification. For CuAAC reactions with azide probes, a similar situation occurred, with the non-polar triazole **14** being easy to prepare and purify (51% yield), and the polar compound **15** being more troublesome (24% yield). Low yields for polar triazoles **13** and **15** prompted us to find better reaction conditions for polar substrate sets. Thus, for triazole **15**, we performed a small-scale solvent screening with an LC-MS conversion analysis. The 9:1 *v*/*v* water: methanol solvent mixture gave the best result (Table S1, electronic supporting information), and, thus, we used it for the preparative synthesis of **15**, which proceeded in good yield (60%). Good screening yields achieved for compound **15** in water and 9:1 water–methanol solution are also a promising prompt towards future studies on biomolecule labelling, run in water and buffers.

### 2.4. Spectral and Physicochemical Properties of the Safirinium-Based Probes and Their Triazole Derivatives

**Figure 4:**
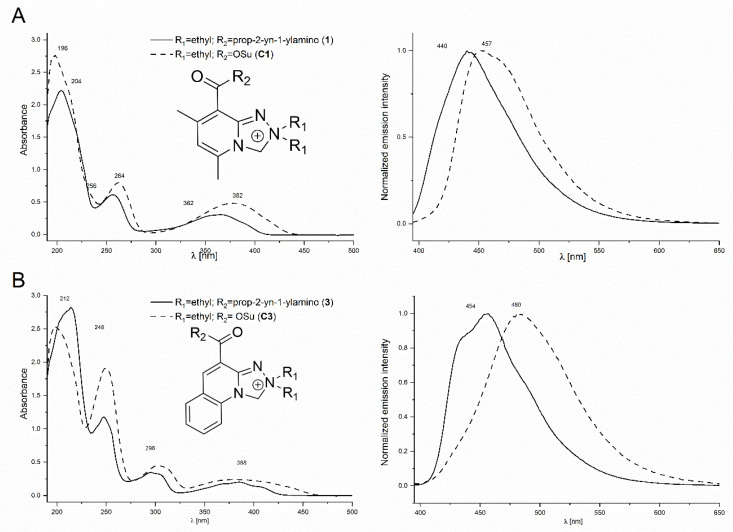
Comparison of UV–Vis absorption and emission spectra of Safirinium-based alkyne probes and their parent NHS esters: (**A**) alkyne probe **1** and NHS ester **C1** (diethyl Safirinium P); and (**B**) alkyne probe **3** and NHS ester **C3** (diethyl Safirinium Q). All absorption spectra were measured for 10^−4^ mol/dm^3^ acetonitrile solutions and emission spectra were measured for 10^−7^ mol/dm^3^ acetonitrile solutions of respective substances.

For the synthesized probes and triazole derivatives, we evaluated some fundamental spectral and physicochemical properties to verify their potential for fluorescent labelling. Initially, we compared the UV–VIS absorption and emission spectra for the synthesized alkyne and azide probes with those of the parent Safirinium NHS esters (Figure 4). Compared to the Safirinium P and Safirinium Q active esters or zwitterions, the spectra recorded for the alkyne and azide derivatives are similar, the longest-wave maximum is shifted by ca. 20 nm towards a shorter wavelength, and its absorption intensity is reduced. Likewise, for the emission spectra, the emission maxima are shifted by ca. 20 nm towards a shorter wavelength. The absorption/emission parameters of selected probes are given in Table 1. Importantly, the fluorescent properties are conserved upon amide bond formation and the subsequent CuAAC reaction. The fluorescence quantum yields determined in water and acetonitrile (Table 1) demonstrate that clearly. In addition, a very high quantum yield was obtained for alkyne probe **1** in water**.** A significant drop in quantum yield was observed only for the lipophilic triazole **11** in the aqueous solution, possibly due to aggregation.

To assess the chemical stability of Safirinium-based alkyne probes for RP-HPLC analysis, we developed a preliminary RP-HPLC method with fluorescent detection for the alkyne probe **1**. This was accomplished using an LC-PCN column and a simple isocratic elution approach. Subsequently, we prepared a 10^−6^ M aqueous solution of probe **1** and kept it exposed to natural daylight on a laboratory bench for 7 days. RP-HPLC analyses performed after that time revealed that the peak area decreased by 50%; thus, its half-life can be estimated at 168 h. Assuming pseudo-first-order kinetics, it can be calculated that probe **1** can remain stable (95%) under such conditions for 12 h. This test indicated that, without using any additional safety precautions (like storage in the dark or cold), the Safirinium alkyne probe **1** can remain stable in an aqueous solution exposed to daylight for 12 h. Of note, the decomposition of probe **1** did not produce fluorescent impurities that could impair the HPLC analysis.

**Figure 5:**
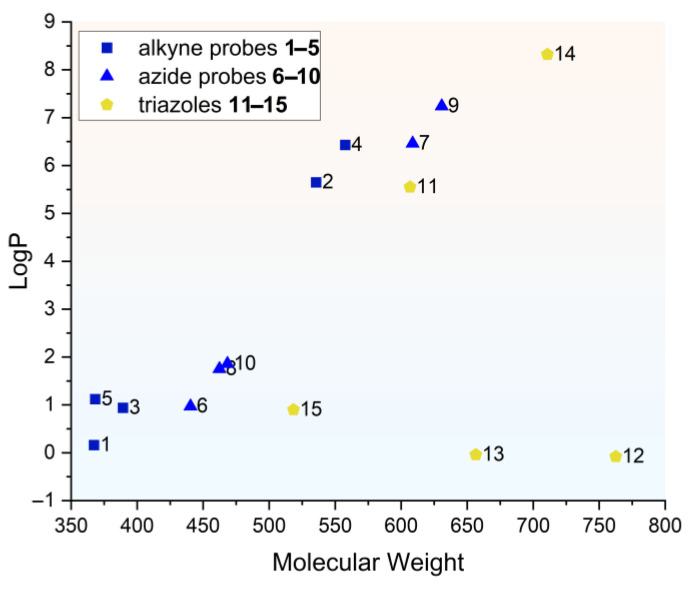
Octanol–water partition coefficient (LogP) values calculated for alkyne-functionalized probes **1**–**5** (■), azide-functionalized probes **6**–**10** (▲), and triazoles **11**–**15** (⬟), plotted against the corresponding values of molecular weight (MW) for each compound. MarvinSketch 22.22 software was used for LogP calculation.

In addition to evaluating their fluorescent properties, chemical stability, and usefulness for RP-HPLC analysis, we assessed the lipophilicity of the synthesized alkyne and azide probes **1**–**10** using computational LogP predictions with MarvinSketch version 22.22 software [39]. We found that the predicted LogP values (Figure 5) range from around 0 to over 7, depending on the substituents in the molecule—most importantly, the length of the alkyl chains attached to the quaternary ammonium atom. The lowest LogP values were obtained for diethyl (probes **1**, **3**, **5** and **6**, **8**, **10**) and the highest ones for dioctyl substitution (probes **2**, **4**, **7**, and **9**). Due to the larger size and lipophilicity of the azidoalkyl fragment, the azide probes **6**–**10** exhibited higher lipophilicity than analogous alkyne counterparts **1**–**5** containing the smaller propargyl fragment.

### 2.5. Cellular Uptake and Distribution of Probes and Triazole Derivatives Observed Using Fluorescence Microscopy

**Figure 6:**
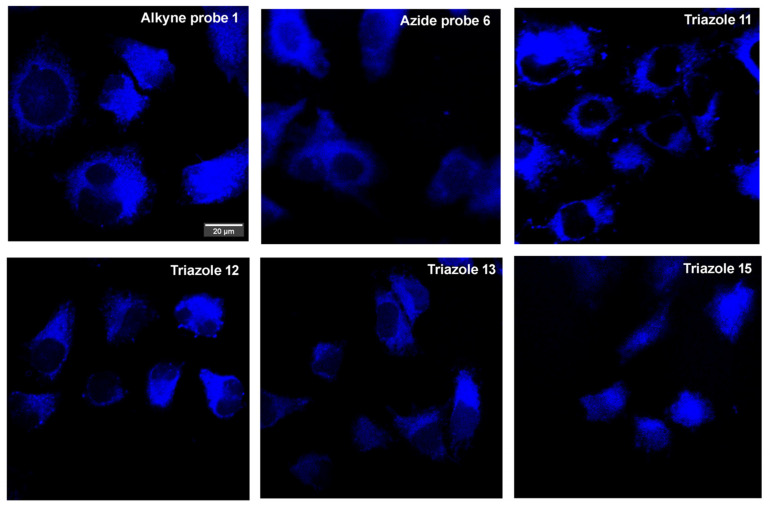
Cellular uptake and intracellular distribution of selected probes (**1** and **6**) and triazole derivatives (**11**, **12**, **13**, and **15**) in Chinese hamster ovary cells. CHO-K1 cells were cultured overnight and then incubated with the tested compounds (10 μM). Representative images (100× magnification) illustrating intracellular distribution of the compounds in CHO-K1 cells were captured after 30 min of incubation with the tested compounds. Scale bar = 20 μm.

In the next move, we examined the application of selected probes (alkyne **1** and azide **6**) and triazole derivatives (**11**, **12**, **13,** and **15**) for fluorescent microscopic observation. For this purpose, we used CHO-K1 cells, and the slides were prepared in accordance with the procedure described in the experimental section, which was similar to the methods used in a previous study on pyridine derivatives [40]. We observed that both the Safirinium based probes (**1** and **6**) and their triazole derivatives remain stable and fluorescent under the microscopic observation conditions. These probes were capable of entering the cytoplasm of the cells; however, they did not penetrate the nuclei, as demonstrated in Figure 6. There was no significant signs of cell death or damage upon staining with Safirinium derivatives. The observation used standard equipment and settings with no tweaks or addons necessary. Therefore, for this first study, we can conclude that the presented probes and their triazole derivatives can be used for fluorescent microscopy imaging applications. This is in good agreement with the earlier research on fluorescent staining with Safirinium NHS esters, like compounds **C1**–**C4** (Figure 2A) [25,31].

### 2.6. Safirinium Azide/Alkyne Probes and Their Conjugates Are Safe in CHO-K1 and NIH-3T3 Cells

**Figure 7:**
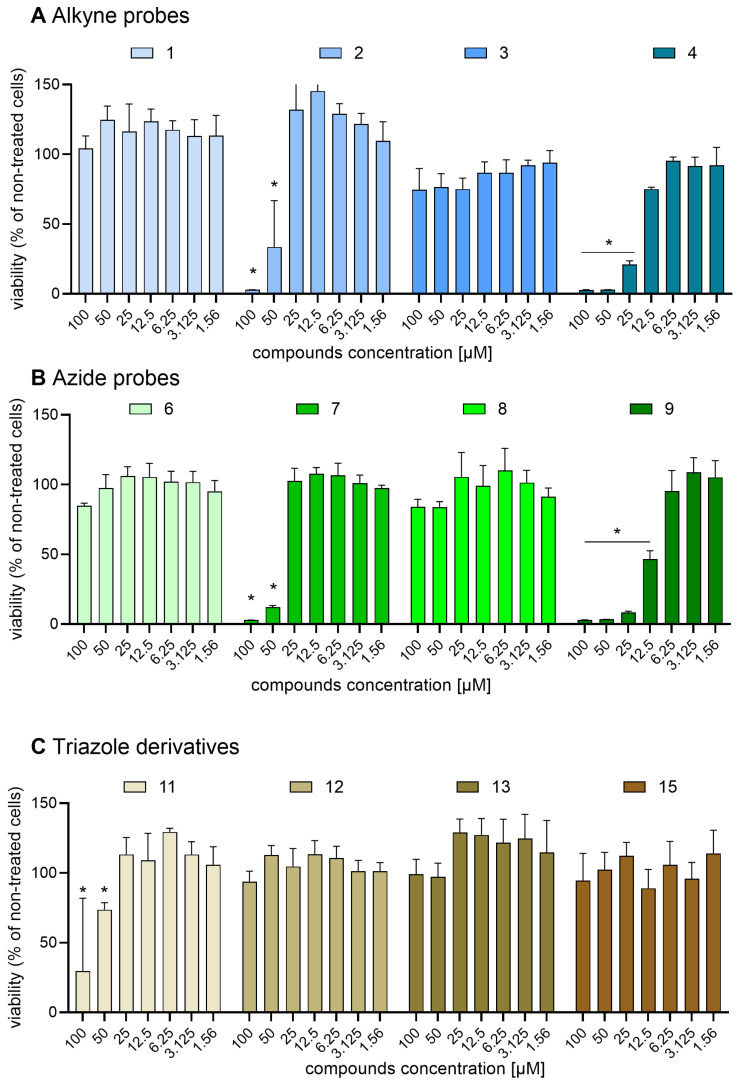
Effect of Safirinium-based probes and their triazole derivatives on Chinese hamster ovary (CHO-K1) cells viability: (**A**) alkyne probes **1**–**4**; (**B**) azide probes **6**–**9**; and (**C**) triazoles **11**–**13** and **15**. CHO-K1 cells were cultured overnight and then incubated in growing concentrations of the compounds. Cell viability was determined by the MTT assay after 24 h of incubation with the tested compounds. Each bar represents mean (±S.E.M.) percentage of viability (in comparison to non-treated cells normalized to 100%). The asterisk-(*) marked results were considered statistically significant at the *p* level of 0.05, n = 4 (Kruskal–Wallis test with Dunn’s post hoc test).

**Figure 8:**
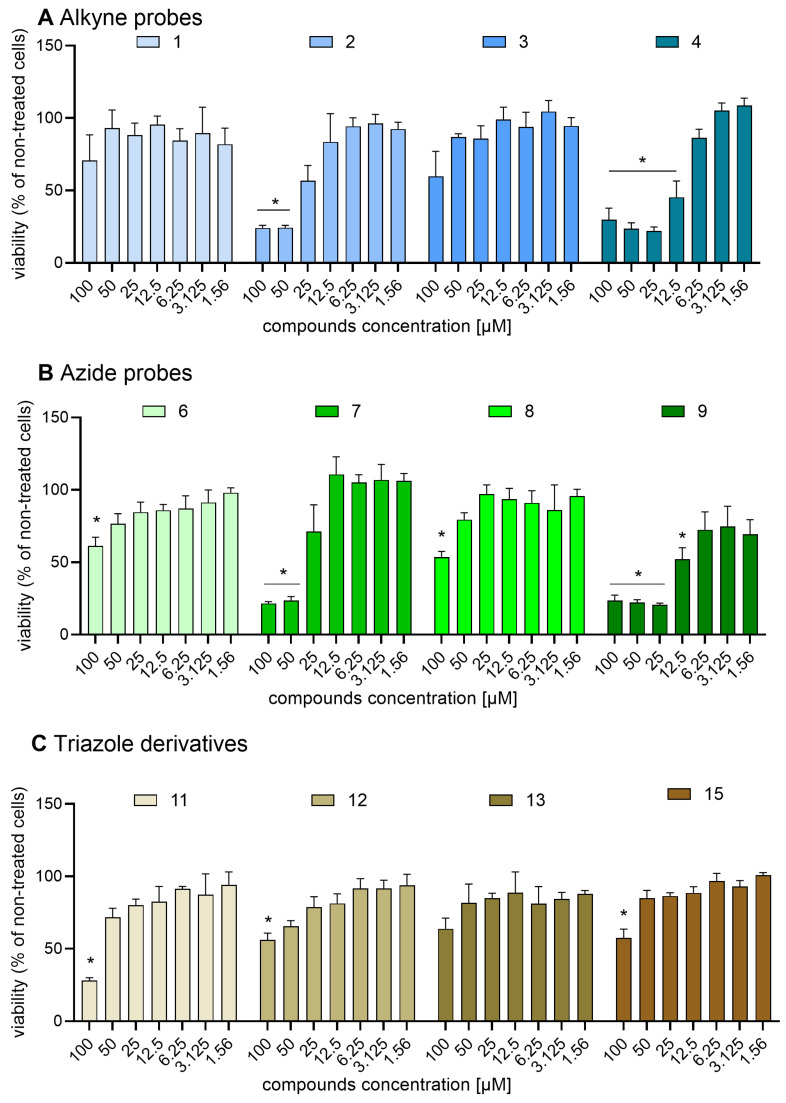
Effect of Safirinium-based probes and their triazole derivatives on mouse fibroblast cell line (NIH-3T3) viability: (**A**) alkyne probes **1**–**4**; (**B**) azide probes **6**–**9**; and (**C**) triazoles **11**–**13** and **15**. NIH-3T3 cells were cultured overnight and then incubated in growing concentrations of the compounds. Cell viability was determined by the MTT assay after 24 h of incubation with the tested compounds. Each bar represents mean (±S.E.M.) percentage of viability (in comparison to non-treated cells normalized to 100%). The asterisk (*) marked results were considered statistically significant at the *p* level of 0.05, n = 4 (Kruskal–Wallis test with Dunn’s post hoc test).

Previous studies on Safirinium dye derivatives revealed that a *N*,*N*-dihexyl Safirinium-P amide conjugate exhibited a cytotoxic effect with an IC_50_ value of 5.44 μM against melanoma cells [27]. This compound differs from those reported herein by the length of the R1 substituent: hexyl vs. ethyl and octyl. This finding prompted us to assess alkyne probes **1**–**4** (Figure 7A and Figure 8A), and azide probes **6**–**9** (Figure 7B and Figure 8B), as well as triazole derivatives **11**–**13** and **15** (Figure 7C and Figure 8C) against two representative cellular models: an epithelial cell line generated from Chinese hamster ovary (CHO-K1, Figure 7) and a fibroblast cell line isolated from a mouse of NIH/Swiss embryo (NIH-3T3, Figure 8).

We found that the majority of the tested compounds were safe against the cell lines used in the analysis. Significant cytotoxicity against both cell lines was observed only for two compounds: alkyne probe **4** (CHO-K1 IC_50_ = 17.30 µM; NIH-3T3 IC_50_ = 8.7 µM) and azide probe **9** (CHO-K1 IC_50_ = 10.97 µM; NIH-3T3 IC_50_ = 13.02 µM). For the NIH-3T3 line, two more compounds were found to be cytotoxic: alkyne probe **2** (NIH-3T3 IC_50_ = 17.48 µM) and azide probe **7** (NIH-3T3 IC_50_ = 26.39 µM). Out of these compounds, only one (**4**) reduced the viability of one cell line (NIH-3T3) after a 24 h-long incubation by half at concentrations below 10 µM, with all of them safe against CHO-K1 up to the 10 micromolar concentration used for microscopic staining. Simultaneously, other alkyne and azide probes, as well as all tested triazole derivatives, did not cause a significant decrease in the viability of both cell lines at concentrations of 25 µM.

Interestingly, the probes **2**, **4**, **7,** and **9** all shared one structural motif: the *N,N*-dioctyl substitution at the ammonium nitrogen (R^1^ = octyl), resembling the dihexyl substitution pattern in the cytotoxic agents described earlier.

These results allow us to conclude that the diethyl Safirinium derivatives we developed can be safely used as fluorescent probes, while extending the R^1^ chains to 6 or more carbon atoms can lead to cytotoxic properties.

## 3. Conclusions

In summary, we synthesized ten novel alkyne- and azide-functionalized fluorescent molecular probes (**1**–**10**) based on the Safirinium P and Q fluorophores, as well as five new 1,2,3-triazole derivatives (**11**–**15**) of these probes. For these compounds, we developed efficient purification protocols using NaBr-modified normal-phase column chromatography. The resulting probes are stable, exhibit spectral properties similar to Safirinium dyes, and can be analyzed with RP-HPLC using fluorescent detection. Likewise, triazole derivatives maintain their fluorescent properties and stability. Polar triazole derivatives of Safirinium probes like compound **15** can be easily synthesized in aqueous solutions (water or 9:1 H_2_O: MeOH), which is a promising prompt towards biomolecule labelling in water and buffers. Both the azide and alkyne probes, along with their triazole derivatives, are suitable for fluorescent microscopy imaging. The diethyl Safirinium (R^1^ = ethyl) derivatives were safe against CHO-K1 and NIH-3T3 cells, while the dioctyl Safirinium (R^1^ = octyl) derivatives showed some cytotoxic effects, particularly against the NIH-3T3 cell line.

Therefore, the Safirinium-based fluorescent molecular probes open avenues for efficient labeling and should find application in the fluorescent derivatization of drugs containing azide or alkyne groups (zidovudine and erlotinib) or the construction of fluorescent protein, antibody, and nucleic acid conjugates, used in anti-cancer drug development. The cytotoxicity of dioctyl Safirinium species might pave the way towards novel click-based theranostics.

## 4. Materials and Methods

### 4.1. General Information

The starting materials were purchased from Merck, Tokyo Chemical Industry (TCI), and Fluorochem. They were used without further purification. Solvents were purchased from Chempur. The ^1^H NMR (500 MHz) and ^13^C NMR (125 MHz) spectra were taken in CDCl_3_, CD_3_OD, and D_2_O, on a 500 MHz JEOL JNMECZR500 RS1 spectrometer. The ^1^H NMR (300 MHz) and ^13^C NMR (75 MHz) spectra were taken on a Varian Mercury spectrometer. Liquid chromatography−mass spectrometry (UPLC−MS) analyses were performed on an Acquity TQD apparatus (Waters), equipped with an Acquity UPLC BEH C18 1.7 mm 2.1 × 100 mm column, eluting with 0.3 mL/min of water−acetonitrile mixture containing 0.1% of HCOOH, in 5−100% acetonitrile gradient over 10 min, followed by 2 min of 100% acetonitrile (also containing 0.1% HCOOH). The detection was carried out by e(λ) diode array detector and ESI-MS. UV–VIS absorption spectra were measured on a UV-2900 Spectrophotometer (VWR-Hitachi, Tokyo, Japan). Fluorescence spectra were measured in acetonitrile and water using a Hitachi F-70000 spectrofluorimeter (P VWR-Hitachi, Tokyo, Japan). RP-HPLC analyses with fluorescent detection were performed using a LaChrom Elite system (Merck-Hitachi, Tokyo, Japan).

### 4.2. Synthesis of Safirinium Carboxylic Acids, Zwitterions, and NHS Esters

Safirinium P and Q derivatives were synthesized according to procedures from earlier studies [23]. For small-scale preparations, the NHS esters were purified using preparative RP-HPLC, as described earlier, and, for larger-scale syntheses, the purification was accomplished using chromatographic method described below (sodium-bromide-saturated silica gel column).

### 4.3. Synthesis of Non-Fluorescent Azides and Alkynes

The non-fluorescent azides and alkynes used as substrates in probe synthesis, 5-azidopentanamine and 7-azidoheptanamine [41], tetradecyl azide [42,43], and 2,3,4,6-Tetra-O-acetyl-β-D-glucopyranosyl azide [44], were synthesized using literature-based procedures.

### 4.4. Azide-Related Safety Measures

Organic azides are potentially toxic and explosive; therefore, all work with them requires caution and adequate security measures. Information concerning azide-related hazards and appropriate security measures can be found in the literature [45,46]. Most important ones include avoiding contact between sodium azide and transition metals and chlorinated solvents, as well as exercising caution while heating and evaporating azide-containing mixtures.

### 4.5. Synthesis and Purification of Safirinium Azide and Alkyne Probes ***1***–***10***

**NHS procedure**. To an NHS ester of Safirinium P or Q, dissolved in anhydrous dichloromethane, 1.5 equiv. of propargylamine (amides **1**–**4**) or propargyl alcohol (ester **5**) was added, alongside 1.1 equiv. of finely powdered anhydrous potassium carbonate. The resulting mixture was stirred at room temperature overnight; then, potassium carbonate was filtered off and the filtrate was evaporated under reduced pressure. The dry residue was purified using column chromatography according to procedures described below.

**MsCl procedure**. To a solution of Safirinium P or Q zwitterion (1.0 eq.) in acetonitrile, potassium carbonate (1.0 eq.) was added, followed by an acetonitrile solution of methanesulfonyl chloride (1.0 eq for azidoamines and 1.2 eq for propargylamine). The resulting mixture was stirred at room temperature for 1 h. After that, acetonitrile solution of the amine (azidoamine: 1.5 eq or propargylamine: 1.2 eq.) was added. The reaction was continued for 1 h at room temperature. After that time, potassium carbonate was filtered off and the filtrate was evaporated under reduced pressure. Dry residue was purified using column chromatography using procedures described below.

**Chromatographic purification** [35,36]. Silica gel column saturated with NaBr. A chromatographic column was wet-packed with silica gel using dichloromethane: methanol 9:1 *v*/*v* solvent mixture; next, it was washed with at least 7 column volumes of 12% *m*/*v* NaBr solution in methanol. After the column was saturated with NaBr, the compound for purification was introduced and elution commenced with 9:1 *v*/*v* dichloromethane: methanol mixture as eluent. For more polar compounds, higher methanol concentrations can be used (e.g., 85:15).

A modification of this method could also be used: silica gel column eluted with sodium-bromide-containing eluent. The column is wet-packed in a standard fashion, using 3% *m*/*v* methanolic sodium bromide solution instead of methanol as a component of eluent, for example, a 9:1 *v*/*v* dichloromethane: methanolic NaBr mixture. Caution: for smaller methanol concentrations, the amount of NaBr should be reduced to avoid precipitation.

TLC plates can either be prepared by soaking in saturated methanolic NaBr and drying prior to DCM: methanol elution or developed using an eluent with 3% w/v NaBr in methanol (e.g., 9: 1 *v*/*v* DCM: 3% methanolic NaBr).

Caution: Chromatographic purification using NaBr-saturated column or NaBr-containing eluent causes ammonium salt counterion change to Br^-^ and presence of sodium bromide in the collected fractions. The NaBr can be removed by redissolving the purified compound in dichloromethane, diethyl ether, acetone, or acetonitrile and filtration.

**2,2-Diethyl-5,7-dimethyl-8-[(prop-2-yn-1-yl)carbamoyl]-2H,3H-[1,2,4]triazolo [4,3-a]pyridin-2-ium bromide 1,** yield: 52% (NHS procedure), 41% (MsCl procedure); BrC_16_H_23_N_4_O, MW 367.27. ^1^H NMR (300 MHz, CDCl_3_): δ ppm 8,40 (t, *J* = 4.7 Hz, 1H), 6.55 (s, 2 H), 5.99 (s, 1H), 4.11 (dd, *J* = 5.0 Hz, 2.6 Hz, 2H), 3.99–4.08 (m, 2H), 3.69–3.83 (m, 2H), 2.58 (d, *J* = 7.6 Hz, 6H), 2,22 (t, *J* = 2.3 Hz, 1H), 1,43 (t, *J* = 6.7 Hz, 6H); ^13^C NMR (75 MHz, CDCl_3_): δ ppm 163.0, 159.6, 156.5, 144.3, 113.9, 107.4, 79.6, 73.0, 71.4, 62.6, 29.6, 29.2, 22.9, 19.7, 8.6; UPLC/MS purity 100.00%, t_r_ = 2.0 min, monoisotopic mass 287.2, [M]^+^ 287.2.

**5,7-Dimethyl-2,2-dioctyl-8-(prop-2-yn-1-ylcarbamoyl)-2,3-dihydro-[1,2,4]triazolo[4,3-a]pyridin-2-ium bromide 2**, yield: 56% (NHS procedure), 26% (MsCl procedure); BrC_28_H_47_N_4_O, MW 535.16; Anal. Calcd. C (62.79%), H (8.85%), N (10.46%), found: C (62.16%); H (8.63%), N (12.07%); ^1^H NMR (300 MHz, CD_3_OD): δ ppm 6.16 (s, 1H), 5.95 (s, 2H), 4.11 (d, *J* = 2.9 Hz, 2H), 3.55–3.76 (m, 4H), 2,64 (t, *J* = 2.6 Hz, 1H), 2.42 (s, 3H), 2.34 (s, 3H), 1.81 (br, s, 4H), 1.29–1.39 (m, 20H), 0.89 (t, *J* = 7.0 Hz, 6H); ^13^C NMR (75 MHz, CDCl_3_): δ ppm 163.0, 160.0, 157.3, 156.4, 144.0, 114.3, 107.6, 79.6, 74.6, 71.5, 67.6, 42.1, 31.7, 29.4, 29.2, 29.1, 26.2, 23.6, 23.1, 23.0, 22.6, 20.1, 14.1 ppm; UPLC/MS purity: 95.31%, t_r_ = 7.24 min, monoisotopic mass 455.37, [M]^+^ 455.4

**2,2-Diethyl-4-(prop-2-yn-1-ylcarbamoyl)-1,2-dihydro-[1,2,4]triazolo[4,3-a]quinolin-2-ium bromide 3,** yield: 67% (NHS procedure), 47% (MsCl procedure); BrC_18_H_21_N_4_O, MW 389.30; ^1^H NMR (300 MHz, CD_3_OD): δ ppm 8.76 (s, 1H), 7.91–7.97 (m, 1H), 7.78–7.87 (m, 1H), 7.35–7.47 (m, 2H), 6.09 (s, 2H), 4.24 (d, *J* = 2.9 Hz, 2H), 3.87–4.00 (m, 4H), 3.06–3.11 (m, 1H), 1.48 (t, *J* = 7.0 Hz, 6H); ^1^H NMR (300 MHz, (CD_3_)_2_SO): δ ppm 8.72 (s, 1 H), 8.54 (t, *J* = 5.6 Hz, 1 H), 8.03 (dd, *J* = 8.2, 1.2 Hz, 1 H), 7.75–7.86 (m, 1 H), 7.39 (t, *J* = 7.3 Hz, 1 H), 7.28 (d, *J* = 8.2 Hz, 1 H), 6.02 (s, 2 H), 4.15 (dd, *J* = 5.6, 2.6 Hz, 2 H), 3.73–3.89 (m, 4 H), 3.21 (t, *J* = 2.3 Hz, 1 H), 1,34 (t, *J* = 7.0 Hz, 6 H); ^13^C NMR (75 MHz, CD_3_OD): δ ppm 173.2, 160.9, 154.1, 145.0, 135.3, 134.5, 131.5, 124.3, 120.6, 115.2, 114.9, 81.0, 74.0, 73.1, 61.1, 29.5, 25.7, 8.4; UPLC/MS purity: 100.0%, t_r_ = 2.95 min, monoisotopic mass: 309.17, [M]^+^ 309.2.

**2,2-Dioctyl-4-(prop-2-yn-1-ylcarbamoyl)-1,2-dihydro-[1,2,4]triazolo[4,3-a]quinolin-2-ium bromide 4**, yield: 64% (NHS procedure), 58% (MsCl procedure); C_30_H_45_N_4_O MW 557.621; ^1^H NMR (500 MHz, CDCl_3_): δ ppm 8.79 (s, 1 H), 8.19 (t, *J* = 5.1 Hz, 1 H), 7.70–7.83 (m, 2 H), 7.51 (d, *J* = 8.2 Hz, 1 H), 7.39 (t, *J* = 7.6 Hz, 1 H), 6.55 (s, 2 H), 4.22–4.35 (m, 4 H), 3.79 (td, *J* = 12.0, 5.0 Hz, 2 H), 2.28 (t, *J* = 2.6 Hz, 1 H), 1.73–1.84 (m, 4 H), 1.35–1.44 (m, 4 H), 1.21–1.30 (m, 16 H), 0.81–0.84 ppm (m, 6 H);^13^C NMR (126 MHz, CDCl_3_): δ ppm 160.3, 154.3, 146.6, 135.6, 134.5, 131.3, 125.3, 120.4, 115.1, 113.8, 78.9, 74.1, 72.1, 68.1, 31.7, 29.9, 29.2, 29.1, 26.1, 23.1, 22.6, 14.1; UPLC/MS purity: 98.03%, t_r_ = 7.14 min, monoisotopic mass: 477.36, [M]^+^ 477.4.

**2,2-Diethyl-5,7-dimethyl-8-((prop-2-yn-1-yloxy)carbonyl)-2,3-dihydro-[1,2,4]triazolo[4,3-a]pyridin-2-ium bromide 5,** yield: 31% (NHS procedure); BrC_16_H_22_N_3_O_2_ MW 368.27; ^1^H NMR (300 MHz, CDCl_3_): δ ppm 6.42 (s, 2H), 5.91 (s, 1H), 4.88 (d, *J* = 2.9 Hz, 2H), 4.02–4.12 (m, 2H), 3.64–3.76 (m, 2H), 2.58 (s, 3H), 2.51 (t, *J* = 2.3 Hz, 1H), 2.36 (s, 3H), 1.46 (t, *J* = 7.0 Hz, 6H); ^13^C NMR (75 MHz, CDCl_3_): δ ppm 173.9, 156.0, 142.5, 142.2, 113.9, 111.4, 75.5, 72.5, 62.6, 52.7, 29.7, 21.4, 20.1, 8.7. UPLC/MS purity: 95.19%, t_r_ = 2.96 min, monoisotopic mass: 288.17 [M]^+^ 288.2.

**8-((5-Azidopentyl)carbamoyl)-2,2-diethyl-5,7-dimethyl-2,3-dihydro-[1,2,4]triazolo[4,3-a]pyridin-2-ium bromide 6**, yield: 78%; (MsCl procedure); BrC_18_H_30_N_7_O, MW 440.39; ^1^H NMR (500 MHz, CDCl_3_): δ ppm 7.88 (t, *J* = 5.4 Hz, 1 H), 6.60 (s, 2 H), 6.01 (s, 1 H), 4.00–4.16 (m, 2 H), 3.79 (dd, *J* = 12.6, 7.2 Hz, 2 H), 3.34–3.40 (m, 2 H), 3.27 (t, *J* = 6.7 Hz, 2 H), 2.60 (s, 3 H), 2.56 (s, 3 H), 1.56–1.65 (m, 4 H), 1.42–1.47 ppm (m, 8 H); ^13^C NMR (126 MHz, CDCl_3_): δ ppm 163.2, 158.8, 156.8, 143.3, 114.1, 108.9, 73.6, 62.6, 51.4, 39.4, 29.8, 29.0, 28.6, 24.3, 22.8, 20.0, 8.9 ppm; UPLC/MS purity: 100.0%, t_r_ = 3.59 min, monoisotopic mass: 360.25, [M]^+^ 360.4.

**8-((5-Azidopentyl)carbamoyl)-5,7-dimethyl-2,2-dioctyl-2,3-dihydro-[1,2,4]triazolo[4,3-a]pyridin-2-ium bromide 7**, yield: 33% (MsCl procedure); BrC_30_H_54_N_7_O, MW 608.71; ^1^H NMR (500 MHz, CD_3_OP): δ ppm 6.12 (s, 1H), 5.81 (s, 2H), 3.71–3.80 (m, 2H), 3.51–3.66 (m, 4H), 3.30–3.33 (m, 2H), 2.35 (s, 3H), 2.28 (s, 3H), 1.72–1.87 (m, 4H), 1.57–1.66 (m, 4H), 1.45–1.51 (m, 2H), 1.27–1.38 (m, 20H), 0.86–0.90 (m, 6H); UPLC/MS purity: 96.52%, t_r_ = 8.01 min, monoisotopic mass 528.44, [M]^+^ 528.4.

**4-((5-Azidopentyl)carbamoyl)-2,2-diethyl-1,2-dihydro-[1,2,4]triazolo[4,3-a]quinolin-2-ium bromide 8**, yield: 61% (MsCl procedure); BrC_20_H_28_N_7_O, MW 462.396; ^1^H NMR (500 MHz, CDCl_3_): δ ppm 1.46–1.49 (m, 8 H) 1.60–1.66 (m, 4 H) 3.27–3.29 (m, 3 H) 3.44–3.52 (m, 3 H) 3.87–3.99 (m, 3 H) 4.31–4.42 (m, 3 H) 6.53 (s, 2 H) 7.38 (t, *J* = 7.63 Hz, 1 H) 7.52 (d, *J* = 8.23 Hz, 1 H) 7.74–7.84 (m, 3 H) 8.03 (t, *J* = 5.58 Hz, 1 H) 8.79 (s, 1 H); ^13^C NMR (126 MHz, CDCl_3_): δ ppm 160.5, 154.8, 146.2, 135.3, 134.3, 131.3, 125.2, 120.6, 115.0, 114.5, 73.0, 63.3, 51.3, 40.0, 29.0, 28.3, 26.9, 24.3, 8.9 ppm; UPLC/MS purity: 100%, t_r_ = 4.37 min, monoisotopic mass 382.23, [M]^+^ 382.3

**4-((5-Azidopentyl)carbamoyl)-2,2-dioctyl-1,2-dihydro-[1,2,4]triazolo[4,3-a]quinolin-2-ium bromide 9**, yield: 69% (MsCl procedure); BrC_32_H_54_N_7_O, MW 630.720; ^1^H NMR (500 MHz, CDCl_3_): δ ppm 8.79 (s, 1 H), 8.01 (t, *J* = 5.7 Hz, 1 H), 7.72–7.80 (m, 2 H), 7.51 (d, *J* = 8.3 Hz, 1 H), 7.35–7.43 (m, 1 H), 6.51 (s, 2 H), 4.32 (td, *J* = 12.0, 5.3 Hz, 2 H), 3.76 (td, *J* = 12.0, 5.0 Hz, 2 H), 3.43–3.53 (m, 2 H), 3.27–3.31 (m, 2 H), 1.75–1.80 (m, 4 H), 1.59–1.68 (m, 6 H), 1.38–1.47 (m, 4 H), 1.21–1.31 (m, 16 H), 0.82–0.85 ppm (m, 6 H); ^13^C NMR (126 MHz, CDCl_3_): δ ppm 160.5, 154.5, 146.2, 135.4, 134.3, 131.2, 125.3, 120.6, 115.0, 114.4, 73.9, 68.1, 51.3, 40.0, 31.7, 29.2, 29.1, 28.6, 26.1, 24.3, 23.1, 22.6, 14.1 ppm; UPLC/MS purity: 100.0%, t_r_= 8.20 min, monoisotopic mass: 550.42. [M]^+^ 550.6

**8-((7-Azidoheptyl)carbamoyl)-2,2-diethyl-5,7-dimethyl-2,3-dihydro-[1,2,4]triazolo[4,3-a]pyridin-2-ium bromide 10**, yield: 78% (MsCl procedure); BrC_20_H_34_N_7_O, MW 468.444; ^1^H NMR (500 MHz, CDCl_3_): δ ppm 7.81 (t, *J* = 5.5 Hz, 1 H), 6.51 (s, 2 H), 5.98 (s, 1 H), 3.98 (dd, *J* = 12.9, 7.0 Hz, 2 H), 3.74 (dd, *J* = 12.9, 7.1 Hz, 2 H), 3.26–3.35 (m, 2 H), 3.19–3.22 (m, 2 H), 2.55 (s, 3 H), 2.50 (s, 3 H), 1.50–1.55 (m, 4 H), 1.41 (t, *J* = 7.1 Hz, 6 H), 1.30–1.34 ppm (m, 6 H); ^13^C NMR (126 MHz, CDCl_3_): δ ppm 163.3, 158.3, 156.7, 143.3, 113.9, 109.1, 73.7, 62.4, 51.4, 39.5, 29.3, 28.8, 26.9, 26.7, 22.6, 20.0, 8.9 ppm; UPLC/MS purity 100.0%, t_r_ = 4.42 min, monoisotopic mass: 388.28, [M]^+^ 388.4.

### 4.6. CuAAC Reactions: Synthesis of Triazoles 11–15

**CuAAC reactions with Safirinium-based alkyne probes.** To a dichloromethane solution of Safirinium-based alkyne probe (1.0 eq.), organic azide (1.05 eq.) was added, followed by 0.05M aqueous CuSO_4_ (10 mol%) and AMTC ligand ((2-{4-[(dimethylamino)methyl]- 1,2,3-triazolyl}cyclohexan-1-ol); 10 mol%) [38]. The resulting mixture was heated to 30 °C and the reaction was started by adding 10 mg/mL aqueous sodium ascorbate (10 mol%) and continued under vigorous stirring at 30 °C overnight. After that time, the solvents were evaporated under reduced pressure and the dry residue was purified using column chromatography as described for the Safirinium probes.

**2,2-Diethyl-5,7-dimethyl-8-(((1-tetradecyl-1H-1,2,3-triazol-4-yl)methyl)carbamoyl)-2,3-dihydro-[1,2,4]triazolo[4,3-a]pyridin-2-ium bromide 11**, yield: 46% (35 mg); BrC_30_H_52_N_7_O, MW 606.698, ^1^H NMR (300 MHz, CDCl_3_): δ ppm 8.77 (br. s, 1H), 7.60 (s, 1H), 6.59 (s, 2H), 6.01 (s, 1H), 4.64 (d, *J* = 5.3 Hz, 2H), 4.32 (t, *J* = 7.3 Hz, 2H), 3.98–4.12 (m, 2H), 3.75–3.87 (m, 2H), 2.61 (br. s, 3H), 2.59 (s, 3 H), 1.84–1.93 (m, 2H), 1.45 (t, *J* = 6.7 Hz, 6H), 1.22–1.32 (m, 22H), 0.84–0.90 (m, 3H); ^13^C NMR (75 MHz, CDCl_3_): δ ppm 163.3, 159.4, 156.5, 143.7, 122.2, 114.0, 108.0, 73.6, 62.5, 50.6, 35.0, 31.9, 30.2, 29.6, 29.6, 29.5, 29.4, 29.3, 29.0, 26.5, 22.9, 22.7, 20.0, 14.1, 8.8; UPLC/MS: purity: 99.42%, t_r_ = 7.98 min, monoisotopic mass: 526.42, [M]^+^: 526.4.

**2,2-Diethyl-4-(((1-((3R,4S,5R,6R)-3,4,5-triacetoxy-6-(acetoxymethyl)tetrahydro-2H-pyran-2-yl)-1H-1,2,3-triazol-4-yl)methyl)carbamoyl)-1,2-dihydro-[1,2,4]triazolo[4,3-a]quinolin-2-ium bromide 12**; yield: 91% (9 mg); BrC_32_H_40_N_7_O, MW 762.615, ^1^H NMR (300 MHz, CDCl_3_): δ ppm 10.92 (s, 1 H), 8.83 (s, 1 H), 7.75–7.84 (m, 2 H), 7.42 (m, 1 H), 7.08–7.15 (m, 1 H), 6.93–6.99 (m, 1 H), 6.84 (m, 1 H), 5.21 (t, *J* = 9.1 Hz, 1 H), 5.10 (t, *J* = 9.1 Hz, 1 H), 4,96 (t, *J* = 9.1 Hz, 1 H), 4,65 (d, *J* = 8.8 Hz, 1 H), 4.23–4.31 (m, 1 H), 4.12–4.20 (m, 1 H), 4.03–3.98 (m, 2 H), 3.75–3.83 (m, 1 H), 2.10 (s, 3 H), 2.05–2.09 (m, 5 H), 2.03 (s, 3 H), 2.01 (s, 3 H), 1.86–1.89 (m, 2 H), 1.59 (m, 6 H); UPLC/MS purity: 84.48%, t_r_ = 4.12 min, monoisotopic mass: 682.28, [M]^+^: *m*/*z* 682.2.

**4-(((1-((2-(hydroxymethyl)-5-(5-methyl-2,4-dioxo-1,2,3,4-tetrahydropyrimidin-1-yl)oxolan-3-yl)-1H-1,2,3-triazol-4-yl)methyl)carbamoyl)- 2,2-diethyl-1,2-dihydro-[1,2,4]triazolo[4,3-a]quinolin-2-ium bromide 13,** yield: 15%; BrC_28_H_34_N_9_O_5_, MW 656.542; ^1^H NMR (500 MHz, CD_3_OD) d 9.00 (s, 1H), 8.73 (s, 1H), 8.13 (s, 1H), 7.92 (d, *J* = 7.81 Hz, 1H), 7.86 (s, 1H), 7.81 (t, *J* = 7.38 Hz, 1H), 7.43 (t, *J* = 7.66 Hz, 1H), 7.30 (d, *J* = 8.24 Hz, 1H), 6.45 (t, *J* = 6.59 Hz, 1H), 5.99 (s, 2H), 5.36–5.45 (m, 1H), 4.33–4.41 (m, 1H), 3.82–3.94 (m, 4H), 3.77 (dd, *J* = 3.44, 12.39 Hz, 1H), 3.02 (d, *J* = 7.31 Hz, 1H), 2.81–2.92 (m, 1H), 2.67–2.76 (m, 1H), 1.86–1.91 (m, 3H), 1.43 (t, *J* = 7.05 Hz, 6H);UPLC/MS purity: 73.0%, t_r_ = 3.55 min; monoisotopic mass: 576.267, [M]^+^ 576.2.

**CuAAC reactions with Safirinium-based azide probes.** To a solution of the azide probe (0.78 mmol, 1 eq.) in dichloromethane or methanol (2 mL), the non-fluorescent alkyne was added (1.18 mmol, 1.5 eq), followed by 0.05M aqueous CuSO_4_ (1.58 mL, 10 mol%), AMTC ligand (((2-{4-[(dimethloamino)methyl]- 1,2,3-triazolyl}cyclohexan-1-ol); 20 mol%) [38] and aqueous sodium ascorbate (10 mol%, in water). The overall organic solvent: water volume ratio was adjusted to 1:2 for **14** (DCM: water) and 1:9 (methanol: water). The reaction proceeded at room temperature overnight. After it was completed, the reaction mixture was evaporated under reduced pressure and the residue was purified by column chromatography.

**5,7-Dimethyl-2,2-dioctyl-8-((5-(4-phenyl-1H-1,2,3-triazol-1-yl)pentyl)carbamoyl)-2,3-dihydro-[1,2,4]triazolo[4,3-a]pyridin-2-ium bromide 14**, yield: 51%; BrC_38_H_60_N_7_O, MW 710.850; ^1^H NMR (300 MHz, CDCl_3_): δ ppm 7.78 (br. s, 1H), 7.73 (m, 3H), 7.36 (t, *J* = 7.4 Hz, 2H), 7.26–7.30 (m, 1H), 6.43 (br. s, 2H), 5.86 (s, 1H), 4.38 (t, *J* = 6.6 Hz, 2H), 3.81 (dt, *J* = 13.3, 6.8 Hz, 2H), 3.64 (br. s, 2H), 3.35 (q, *J* = 5.9 Hz, 2H), 2.50 (br. s, 3H), 2.46 (s, 3H), 1.90–2.00 (m, 2H), 1.66–1.82 (m, 4H), 1.61 (q, *J* = 7.0 Hz, 2H), 1.19–1.40 (m. 22H), 0.81 (t, *J* = 6.9 Hz, 6H);^13^C NMR (126 MHz, CDCl_3_): δ ppm 163.4, 158.1, 156.4, 143.1, 130.6, 129.0, 128.2, 125.6, 113.8, 109.0, 74.3, 67.3, 50.1, 42.1, 38.8, 31.7, 29.5, 29.3, 29.2, 28.5, 26.3, 23.6, 23.4, 23.2, 22.7, 22.5, 20.2, 14.1; UPLC/MS: purity: 82.88%, t_r_ = 7.68 min; monoisotopic mass: 630.49. [M]^+^: 630.66.

**2,2-Diethyl-4-({5-[4-(hydroxymethyl)-1H-1,2,3-triazol-1-yl]pentyl}carbamoyl)-1H,2H-[1,2,4]triazolo[4,3-a]quinolin-2-ium bromide 15**, yield: 60%; BrC_23_H_32_N_7_O_2_, MW 518.460; ^1^H NMR (500 MHz, CD_3_OD) δ ppm 1.44 (t, *J* = 7.16 Hz, 7 H) 1.63–1.70 (m, 3 H) 1.95 (quin, *J* = 7.16 Hz, 3 H) 3.43 (t, *J* = 6.73 Hz, 2 H) 3.84–3.95 (m, 4 H) 4.41 (t, *J* = 6.87 Hz, 3 H) 4.60 (s, 2 H) 6.02 (s, 2 H) 7.32 (d, *J* = 8.31 Hz, 1 H) 7.42 (t, *J* = 7.59 Hz, 1 H) 7.76–7.81 (m, 1 H) 7.91; (d, *J* = 8.02 Hz, 2 H) 8.70 (s, 1 H); 13C NMR: ^13^C NMR (126 MHz, CD_3_OD) δ ppm 161.5, 154.6, 145.0, 134.9, 134.3, 131.1, 124.3, 122.9, 120.7, 114.8, 114.0, 72.6, 61.7, 55.1, 49.8, 39.3, 29.5, 28.3, 23.5; UPLC/MS: purity: 97.73% t_r_ = 3.62 min, monoisotopic mass: 438.26, [M]^+^: 438.26.

### 4.7. UV–VIS Absorption and Emission Measurement, and Fluorescence Quantum Yields

UV–VIS absorption spectra (Figures S1 and S3) were recorded using a Hitachi U-2900 spectrophotometer (VWR-Hitachi, Japan), for 1 × 10^−4^ mol/dm^3^ solutions of the compounds in water and acetonitrile, in 200–500 nm wavelength range. The compounds were dissolved in water, acetonitrile, or water–acetonitrile mixtures to obtain 0.01 mol/dm^3^ stock solutions, which were then diluted 100-fold to obtain the final 10^−4^ mol/dm^3^ concentration. Absorption maxima were listed in Table 1.

Fluorescence spectra were recorded using a Hitachi F-70000 spectrofluorimeter (P VWR-Hitachi, Japan), for 1 × 10^−6^ mol/dm^3^ solutions of the compounds in water and acetonitrile, at 365 nm excitation wavelength, using 5 nm excitation and 2.5 nm emission slits, and 700 V photomultiplier voltage. The spectra (Figures S2 and S4) were recorded in 400–700 nm range. The 1 × 10^−6^ mol/dm^3^ solutions were prepared by 100-fold dilution of the solutions used for absorption measurement.

Quantum yields of fluorescence (Table 1) were determined using the comparative method, from Formula (1) [47], using the recorded absorption and emission spectra and diethyl Safirinium P zwitterion A1 (*ϕ* = 0.755 in water) [23] as standard.

(1) $$\Phi =\Phi_{std}\frac{FA_{std}n_{std}^{2}}{F_{std}An^{2}}$$

where *ϕ*, *ϕ_std_*—quantum yields; *F*, *F_std_*—integrals under fluorescence emission curves; *A*, *A_std_*—absorbance at excitation wavelength, and *n*, *n_std_*—refractive indices, for the compound and standard (std subscript), respectively.

### 4.8. RP-HPLC Analysis

RP-HPLC analyses with fluorescence detection (Method B) were performed on a LaChrom Elite HPLC system, (VWR-Hitachi, Japan), equipped with a L-2130 pump, a L-2485 FL detector, a L-2350 column oven, and an L-2200 autosampler, the system was controlled with EZChrom Elite version 3.2, Agilent software. Separations were performed on a SUPELCOSIL LC-PCN 250 × 4.6 mm column with 5 µm grain size, under 1 mL/min isocratic elution with 50:50 *v*/*v* water: acetonitrile mixture, acidified with 0.1% formic acid. Injection volume was 20 µL; 10^−6^ mol/dm^3^ solution of probe **1** in water was prepared in a clear HPLC sample vial and analyzed directly after preparation and after 7 days (168 h) of standing on a lab bench, exposed to natural daylight at room temperature. Example chromatograms are presented in the electronic supporting information.

### 4.9. Cell Culture

CHO-K1 cell line was derived as a subclone from the parental CHO cell line, which was initiated from a biopsy of an ovary of an adult, female Chinese hamster commercially available in ATCC (CCL-61™). NIH-3T3 cell line was obtained from DSMZ, the German Collection of Microorganisms and Cell Cultures. CHO-K1 and NIH-3T3 cells were cultivated in F-12K Medium (ATCC 30-2004) and Dulbecco’s MEM with Glutamax™ 1 Medium (Gibco, Paisley, UK), respectively. Culture media were supplemented with fetal bovine serum (FBS, Gibco, Thermo Fisher Scientific, Waltham, MA, USA) to a final concentration of 10% and antibiotic to final concentration of 1% (pent/strept, Gibco, Thermo Fisher Scientific, Waltham, MA, USA). Cells were cultured in standard culture conditions (5% CO_2_, 37 °C, 95% humidity).

### 4.10. MTT Test

Cell viability was determined by the 3-(4,5-dimethylthiazol-2-yl)-2,5-diphenyltetrazolium bromide (MTT, Sigma Aldrich, Burlington, MA, USA) assay. Cells were seeded into 96-well plates at density of 2 × 10^4^ cell/well and cultured in the presence of compounds (**1**–**4**, **6**–**9**, **11**–**13**, and **15**) at concentration range 1.56–100 µM for 24 h. The MTT reagent (final concentration 0.5 mg/mL) was added to each well for 3 h, and then formazan crystals were dissolved in DMSO. The absorbance was measured at 570 nm (SpectraMax^®^ iD3, Molecular Devices, San Jose, CA, USA) and the experiment was performed two times in duplicates. Cells viability was expressed as a percentage of viability (in comparison to non-treated cells normalized to 100%). Data were analyzed using GraphPad Prism Software (GraphPad Software, Inc., San Diego, CA, USA, version 10.3.1).

### 4.11. Microscopic Visualization

CHO-K1 cells were seeded at a density of 2 × 10^5^/well in 12-well plates containing cover slips at the bottom and cells were incubated for 24 h. Then, cells were fixed in 3.7% formaldehyde in PBS for 10 min in 37 °C, washed twice with PBS, and stained with compound solution (10 µM) for 30 min. After this time, the cells were rinsed 2 times with Gibco FluoroBrite DMEM and a coverslip was placed on a stock carrier with drops of culture medium. Finally, slides were mounted in ProLong™ Glass Antifade Mountant and visualized under an A1 confocal system built on a Nikon Eclipse Ti-E microscope (Japan) equipped with 405 and 561 nm diode lasers and a Plan Apo 100×/1.4 Oil DIC objective.

## Figures and Tables

**Table 1:** UV–VIS absorption and emission maxima and fluorescence quantum yields for probes **1**, **3**, and **8** and their triazole derivatives **11**, **13,** and **15**, measured for aqueous and acetonitrile solutions. The absorption spectra were measured for 1·10^−4^ mol/dm^3^ solutions. The emission spectra were measured for 1·10^−6^ mol/dm^3^ solutions, at 365 nm excitation wavelength. Fluorescence quantum yields were measured against diethyl Safirinium P zwitterion **A1** (ϕ = 0.755, in water [23]).

Compound	λ Absorption [nm]		λ Emission [nm]		Fluorescence Quantum Yield	
	H_2_O	Acetonitrile	H_2_O	Acetonitrile	H_2_O	Acetonitrile
**1**	210; 246; 351	204; 256; 365	446	443	1.00	0.86
**3**	210; 247; 297; 379	213; 246; 294; 303; 370; 385	462	455	0.62	0.71
**8**	213; 247; 297; 385	212; 246; 294; 303; 368; 382	459	452	0.60	0.68
**11**	254; 351	203; 256; 363	446	443	0.28	0.86
**13**	212; 249; 295; 387	248; 294; 303; 368; 381	462	452	0.59	0.45
**15**	213; 247; 297; 385	212; 246; 294; 302; 369; 382	459	454	0.55	0.53

## Data Availability

The original contributions presented in this study are included in the article/Supplementary Materials. Further inquiries can be directed to the corresponding author.

## Supplementary Materials

The following supporting information can be downloaded at: https://www.mdpi.com/article/10.3390/molecules30030731/s1: (1) Solvent screening results for triazole **15**; (2) Spectral and chromatographic data for azide and alkyne probes **1**–**10**; (3) UV-VIS absorption and emission spectra used for fluorescence quantum yield estimation; (4) Stability of Safirinium alkyne probe **1** in solution: fluorescent HPLC results.
