# Supplementary material for: Safirinium Fluorescent “Click” Molecular Probes: Synthesis, CuAAC Reactions, and Microscopic Imaging
Source: Molecules. 2025 Feb 6;30(3):731. doi: 10.3390/molecules30030731 (PMC11820944; doi:10.3390/molecules30030731)
Supplement: Supplementary file 1 [file molecules-30-00731-s001.zip › molecules-3416972-supplementary.pdf]

Electronic Supporting information for the article:

Safirinium Fluorescent "Click" Molecular Probes: Synthesis, CuAAC Reactions, and Microscopic Imaging

Patryk Kasza [1], Przemysław W. Szafrański [1,*], Joanna Fedorowicz [2], Faustyna Krzysztofiak [1], Krzysztof Pociecha [1], Katarzyna Wójcik-Pszczoła [1], Paulina Koczurkiewicz-Adamczyk [1], Mariusz Kępczynski [3], Jarosław Sączewski [2], Paweł Zajdel [1] and Marek Cegła [1]

[1] Faculty of Pharmacy, Jagiellonian University Medical College, Medyczna 9, 30-688 Kraków, Poland

[2] Faculty of Pharmacy, Medical University of Gdańsk, Al. Gen. J. Hallera 107, 80-416 Gdańsk, Poland

[3] Faculty of Chemistry, Jagiellonian University, Gronostajowa 2, 30-387 Kraków, Poland

* Correspondence: p.szafranski@uj.edu.pl

Content

1. Solvent screening results for triazole **15**
2. Spectral and chromatographic data for azide and alkyne probes **1**–**10**
3. UV-VIS absorption and emission spectra used for fluorescence quantum yield estimation
4. Stability of Safirinium alkyne probe **1** in solution: fluorescent HPLC results

1.Solvent screening results for triazole **15**

Table S1: Results of solvent screening for triazole **15.** The reactions were performed on 1 mg scale, using 1 equiv. propargyl alcohol, 10 mol% CuSO4, 20 mol% AMTC ligand and 10 mol% sodium ascorbate, with 900 RPM shaking (Biosan 300C laboratory thermoshaker, Biosan, Latvia) for 18h at 30 °C. After that time 100-200 µL samples were taken, diluted with acetonitrile and analyzed with LC-MS. Peak areas for product (triazole 15) and substrate (probe 6) were compared.

Organic co-solvent	Substrate **6**: Product **15** peak area ratio	
	water: solvent 9:1	water: solvent 1:1
none	46	-
methanol	56	46
ethanol	1	0.5
dichloromethane	50	45
tetrahydrofuran	40	39
accetonitrile	15	1
isopropanol	39	28
tert-butanol	34	38

2.Spectral and chromatographic data for alkyne and azide probes **1-10**

2,2-Diethyl-5,7-dimethyl-8-[(prop-2-yn-1-yl)carbamoyl]-2H,3H-[1,2,4]triazolo[4,3-a]pyridin-2-ium bromide 1

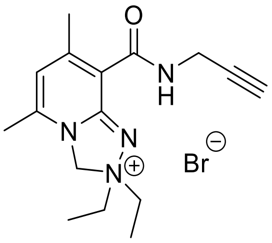

^{1}H NMR (300 MHz, $CDCl_3$)

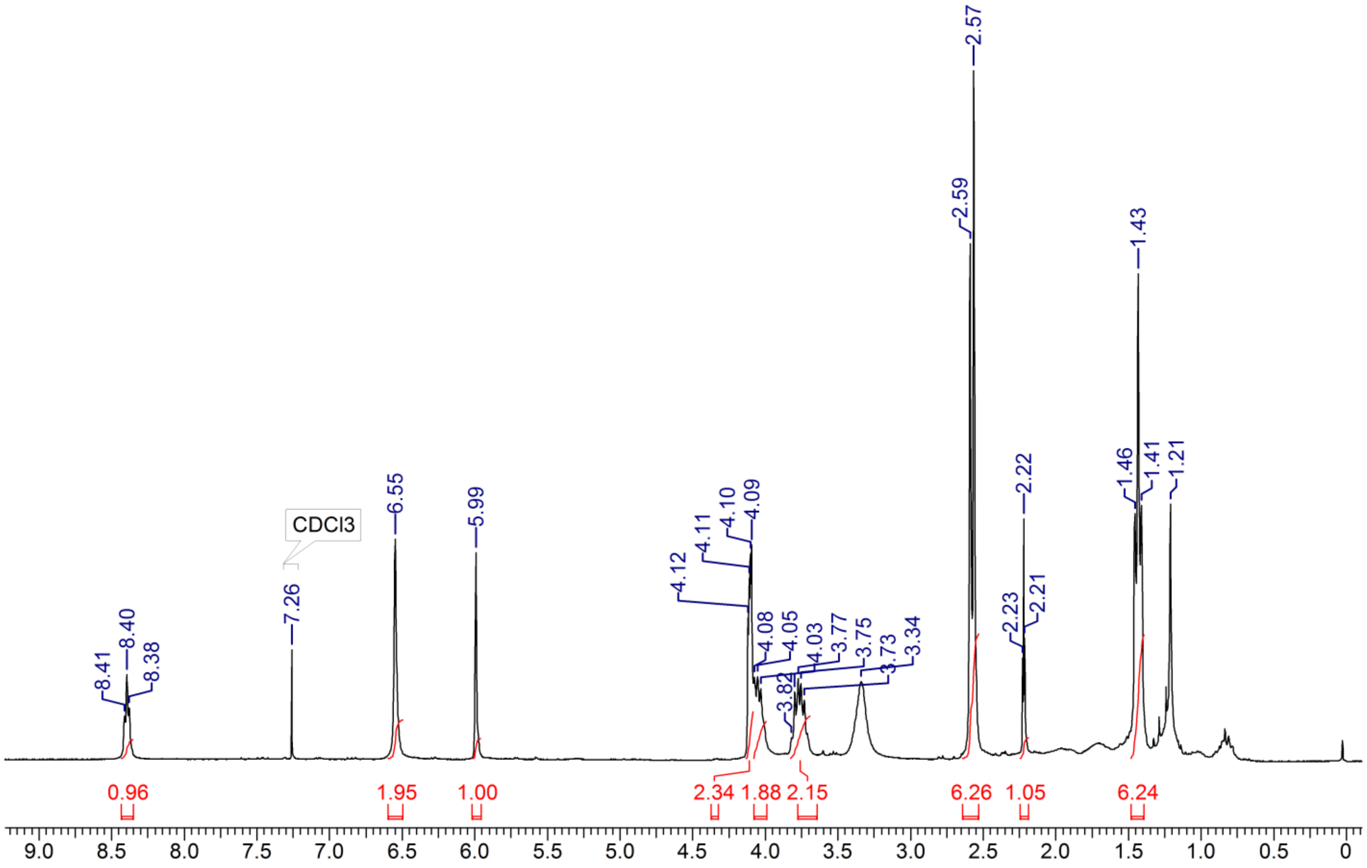

^{13}C NMR (75 MHz, $CDCl_3$)

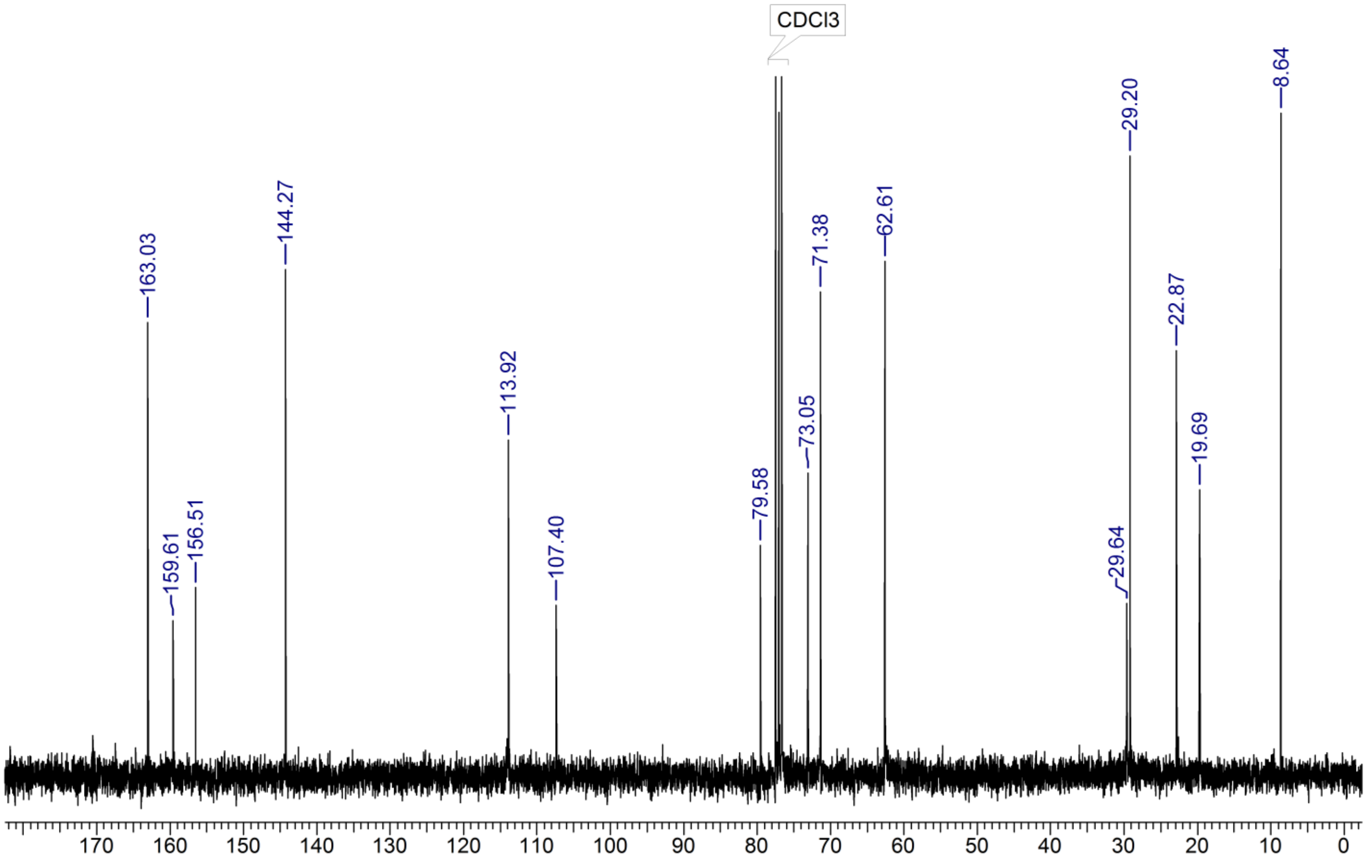

LC (DAD)

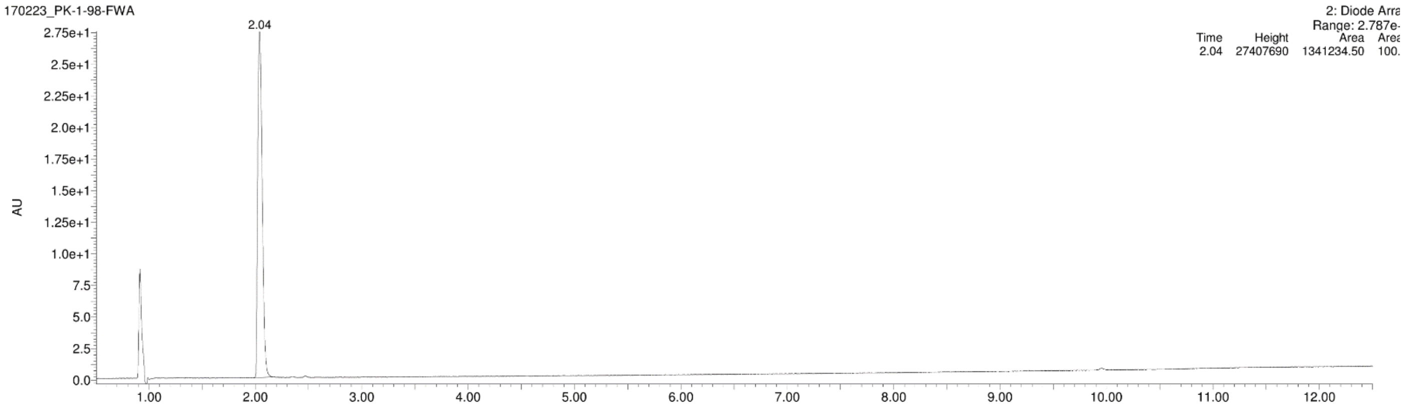

MS

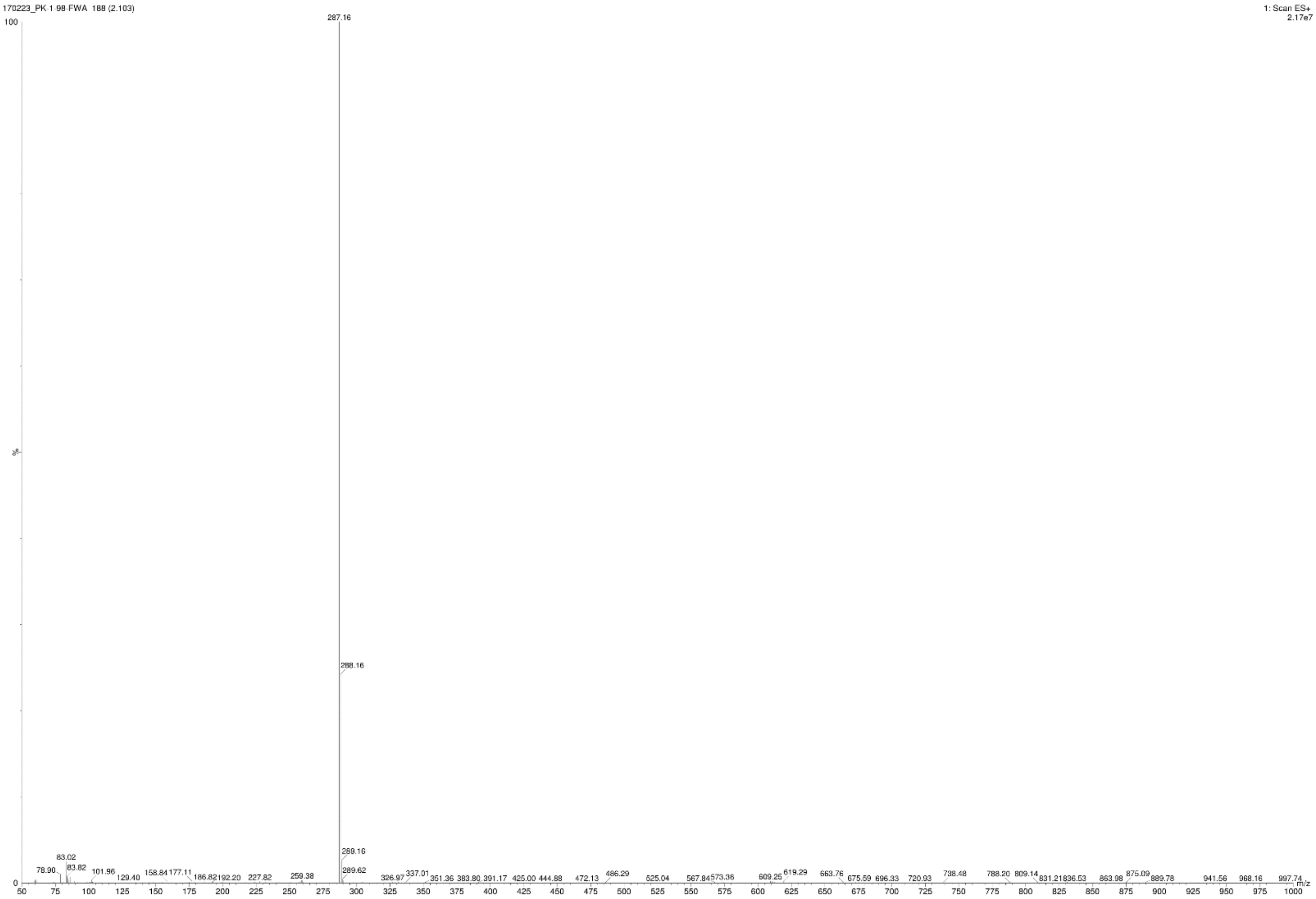
170223_PK-1-98 FWA 188 (2.103)
1: Scan ES+
2.17e7
100
%
0
287.16
288.16
289.16
289.62
83.02
78.90
83.82
101.96
129.40
158.84
177.11
186.82
192.20
227.82
259.38
326.97
337.01
351.36
383.80
391.17
425.00
444.88
472.13
486.29
525.04
567.84
573.36
609.25
619.29
663.76
675.59
696.33
720.93
738.48
788.20
809.14
831.21
836.53
863.98
875.09
889.78
941.56
968.16
997.74
m/z
50 75 100 125 150 175 200 225 250 275 300 325 350 375 400 425 450 475 500 525 550 575 600 625 650 675 700 725 750 775 800 825 850 875 900 925 950 975 1000

5,7-Dimethyl-2,2-dioctyl-8-(prop-2-yn-1-ylcarbamoyl)-2,3-dihydro-[1,2,4]triazolo[4,3-a]pyridin-2-ium bromide 2

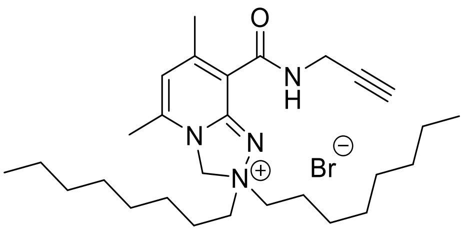

^{1}H NMR (300 MHz, CD_3OD)

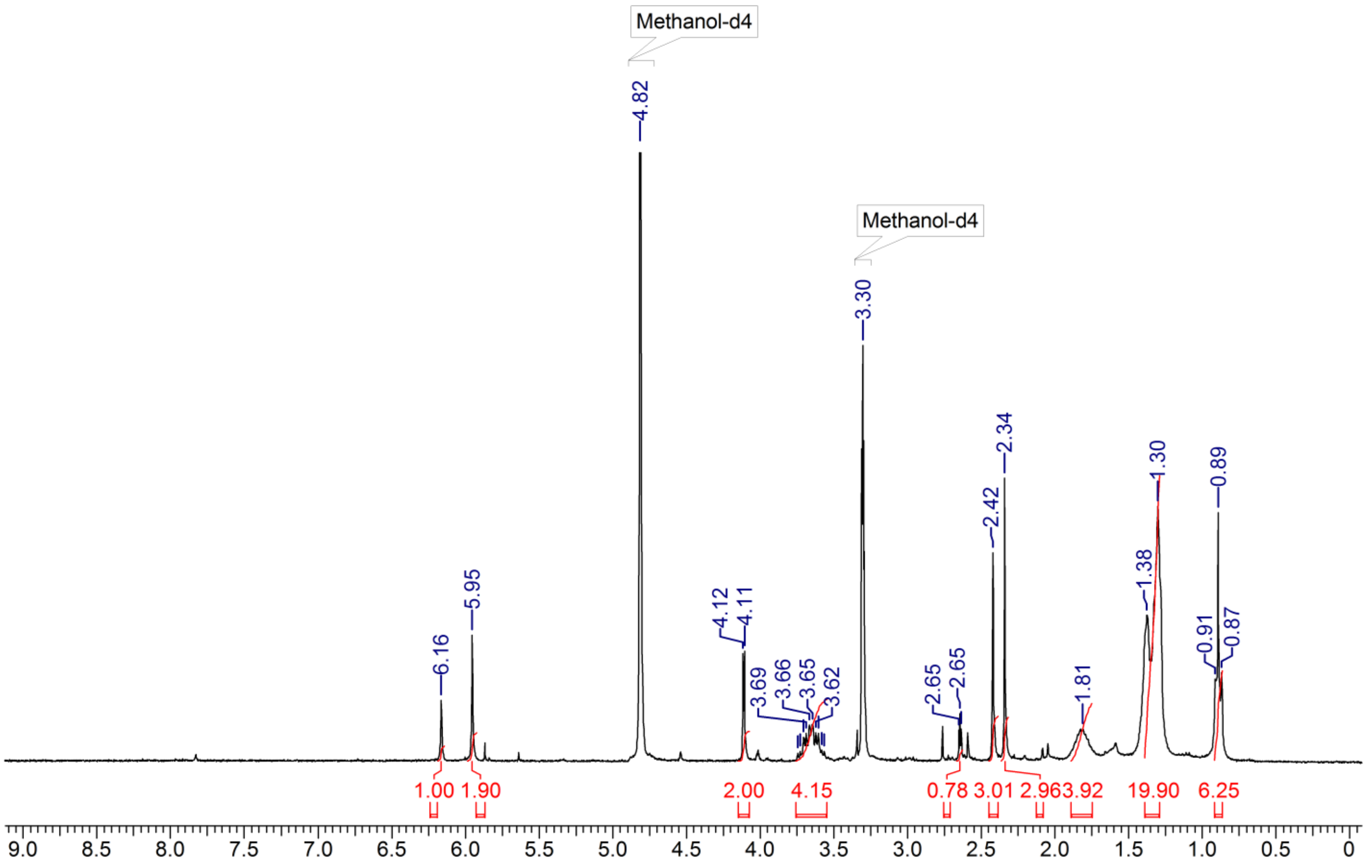

^{13}C NMR (75 MHz, $CDCl_3$)

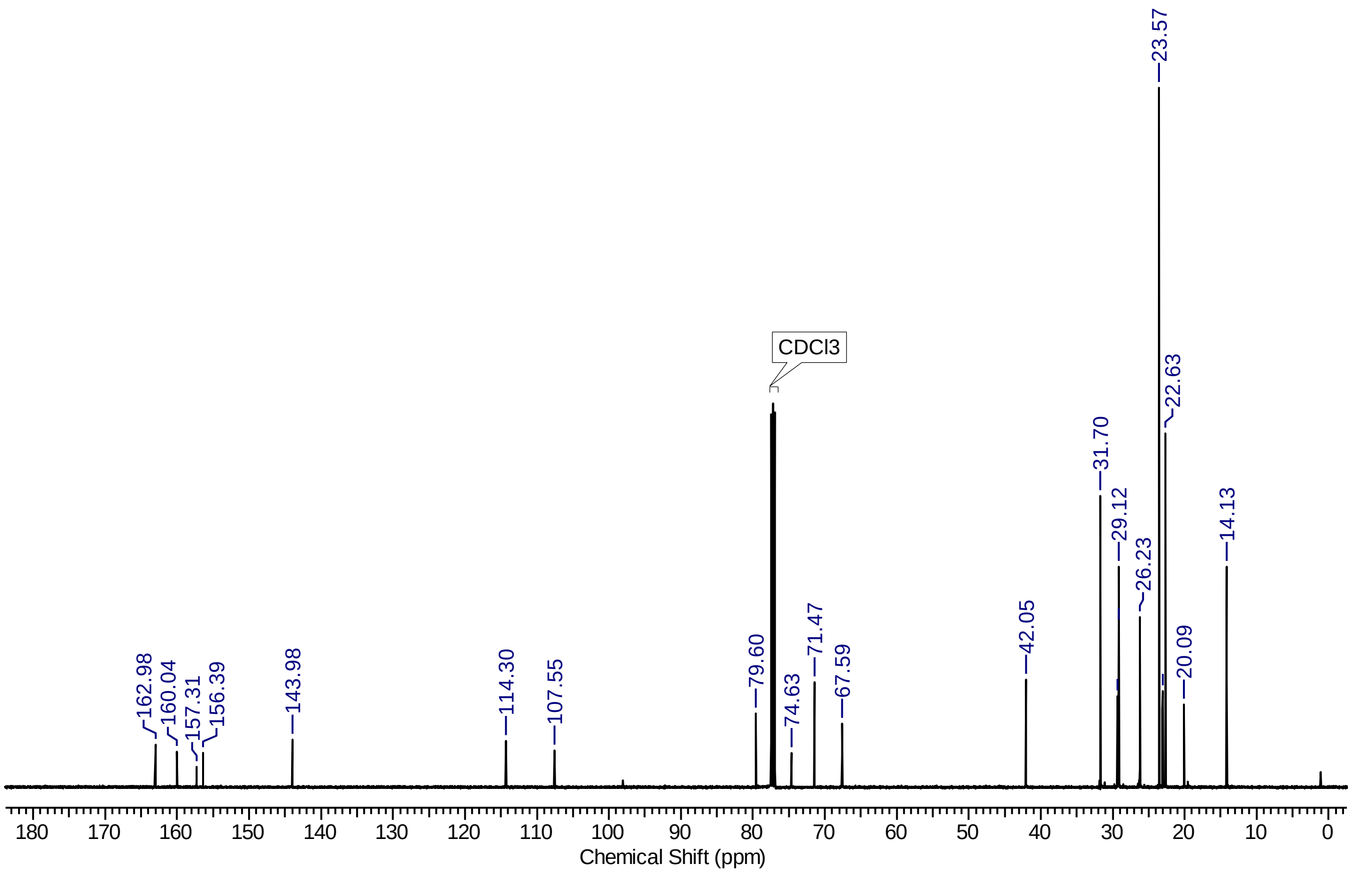

LC (DAD)

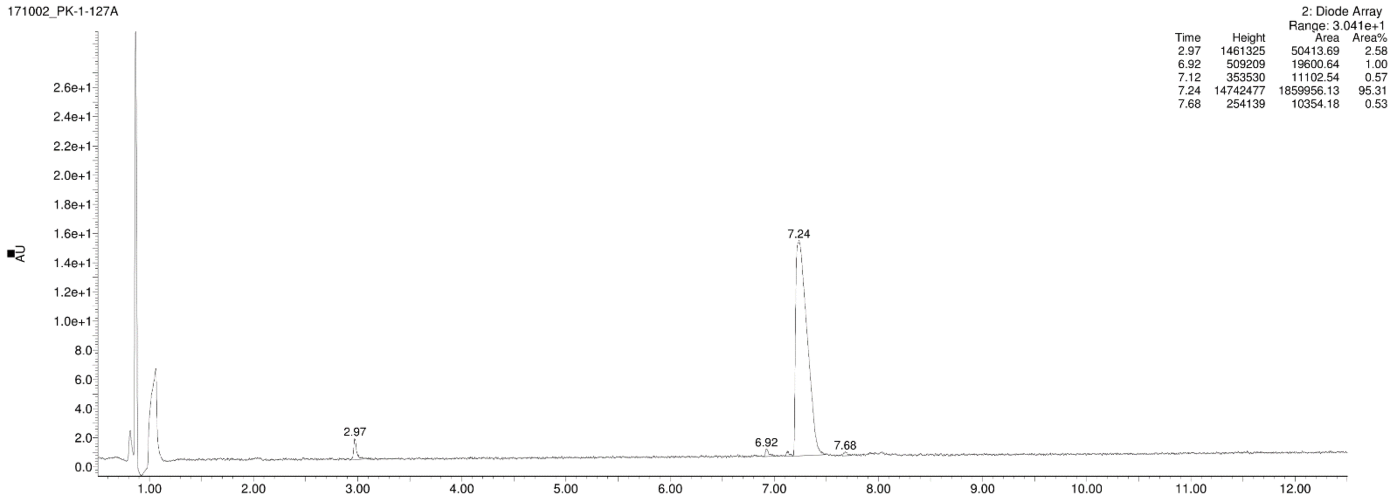

MS

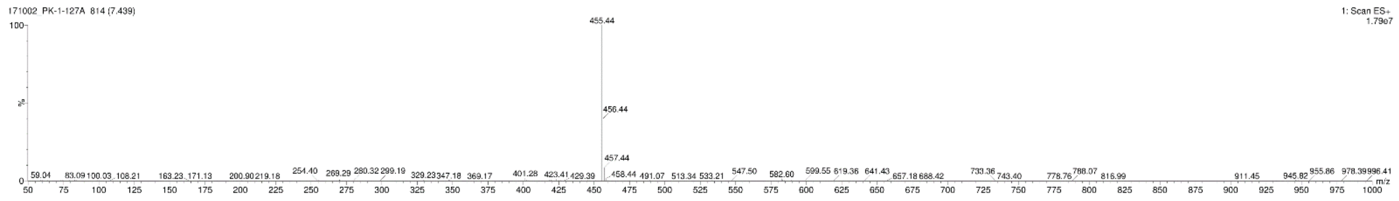

2,2-Diethyl-4-(prop-2-yn-1-ylcarbamoyl)-1,2-dihydro-[1,2,4]triazolo[4,3-a]quinolin-2-ium bromide 3

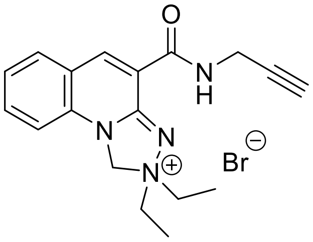

^{1}H NMR (300 MHz, CD_3OD)

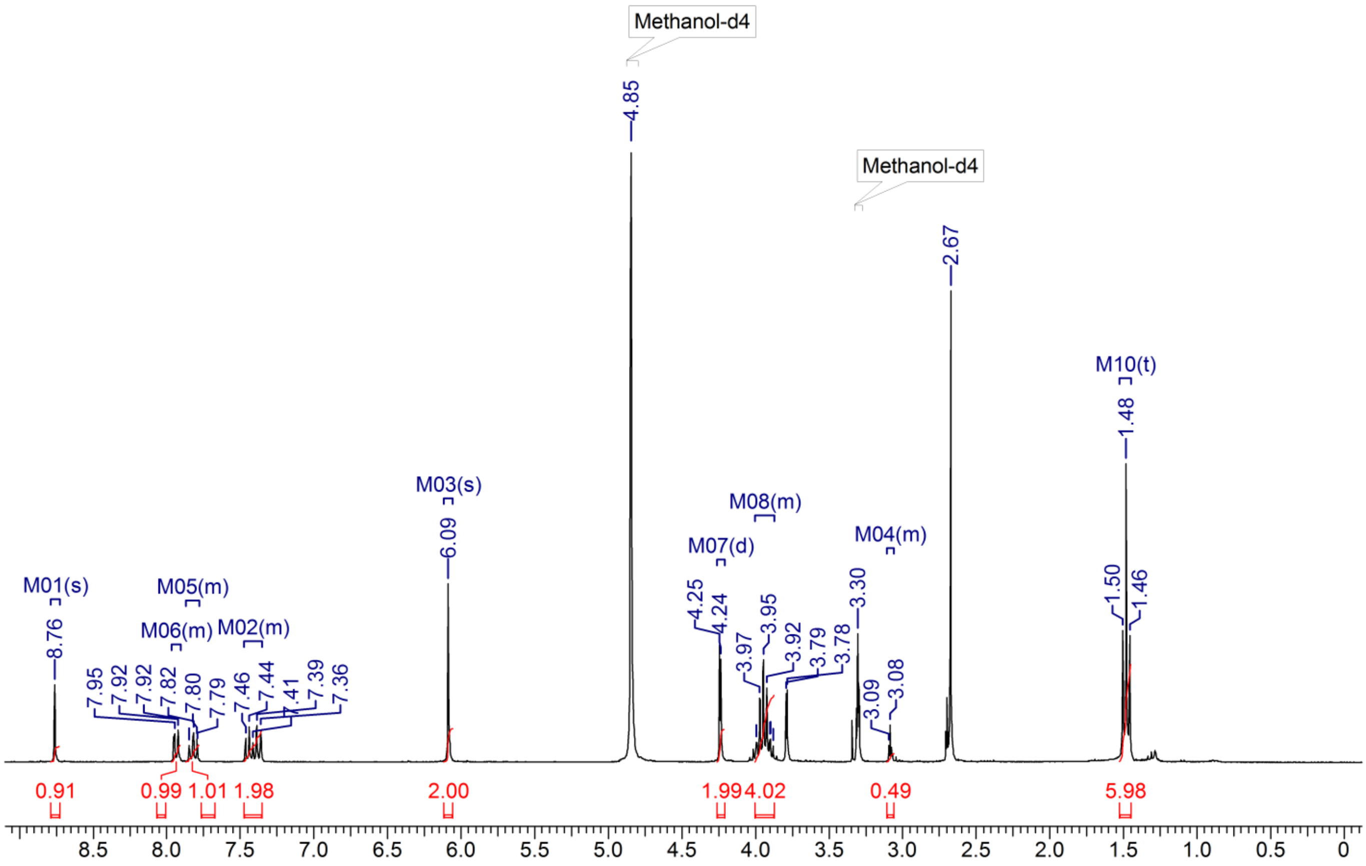

^{1}H NMR (300 MHz, DMSO-d6)

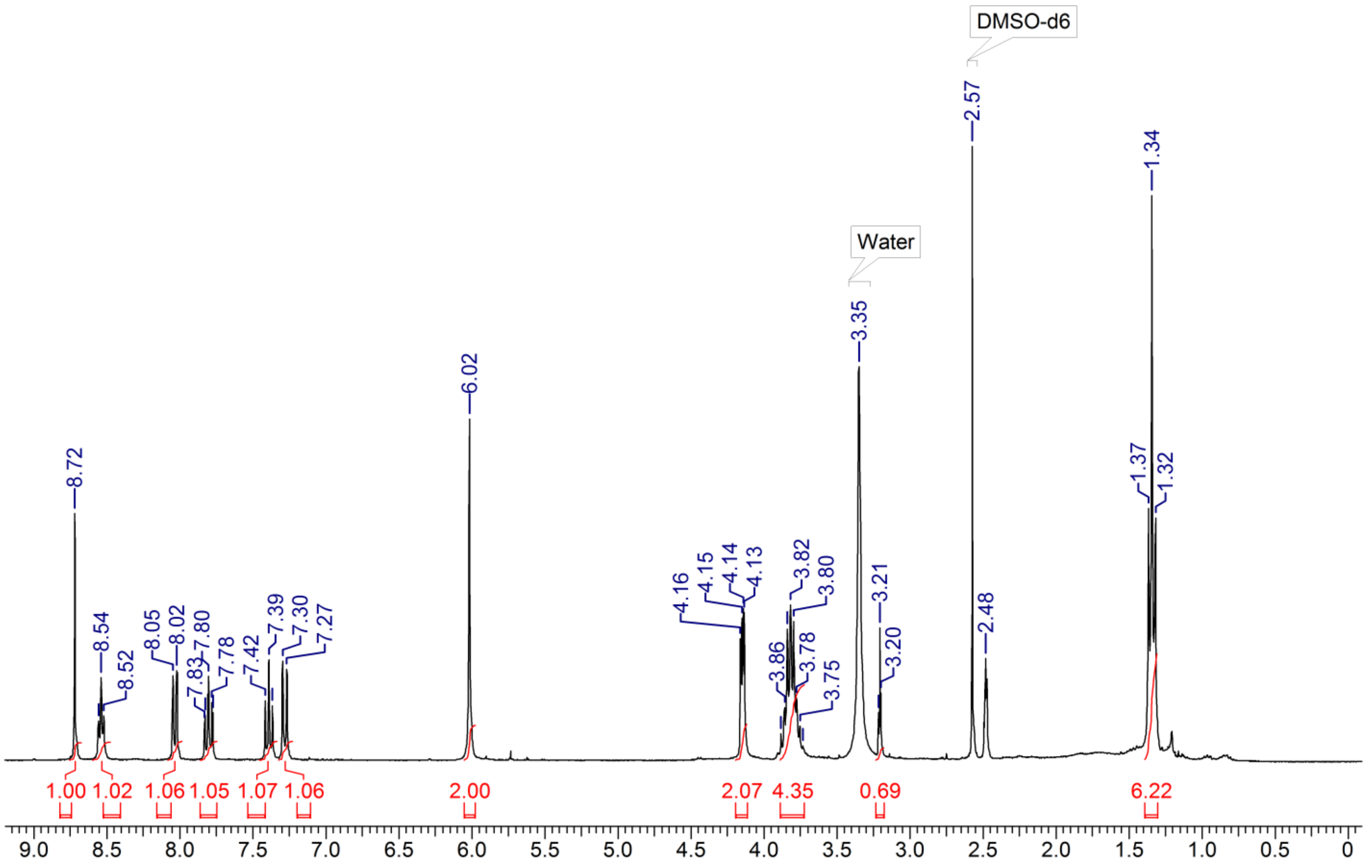

^{13}C NMR (75 MHz, DMSO-d)

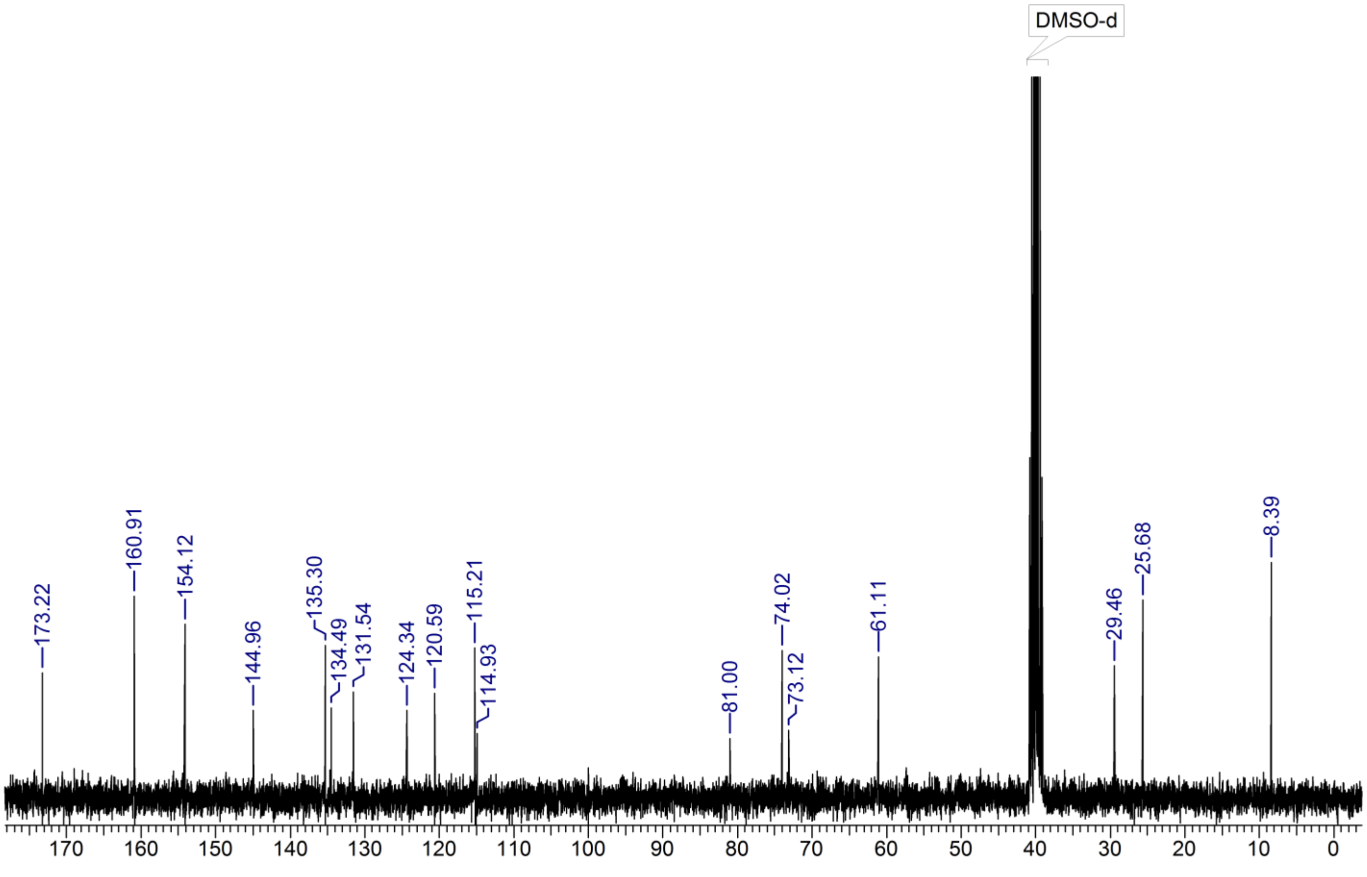

LC (DAD)

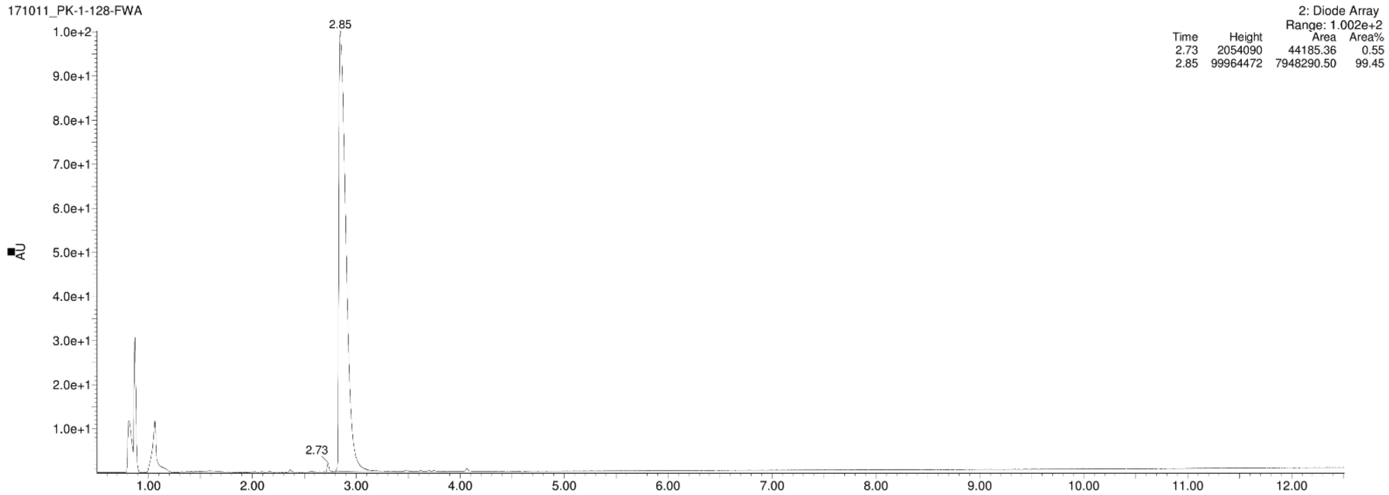
171011_PK-1-128-FWA
2: Diode Array
Range: 1.002e+2
Time Height Area Area%
2.73 2054090 44185.36 0.55
2.85 99964472 7948290.50 99.45
2.85
2.73
AU
1.0e+2
9.0e+1
8.0e+1
7.0e+1
6.0e+1
5.0e+1
4.0e+1
3.0e+1
2.0e+1
1.0e+1
1.00 2.00 3.00 4.00 5.00 6.00 7.00 8.00 9.00 10.00 11.00 12.00

MS

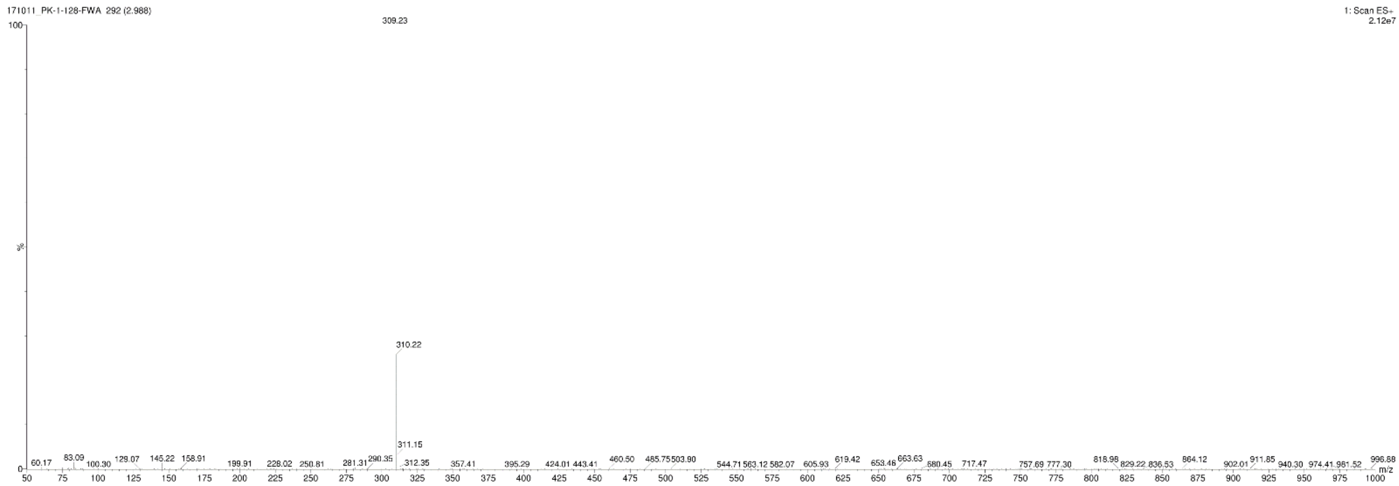
171011_PK-1-128-FWA 292 (2.988)
1: Scan ES+
2.12e7
309.23
310.22
311.15
%
100
0
m/z
50 75 100 125 150 175 200 225 250 275 300 325 350 375 400 425 450 475 500 525 550 575 600 625 650 675 700 725 750 775 800 825 850 875 900 925 950 975 1000

2,2-Dioctyl-4-(prop-2-yn-1-ylcarbamoyl)-1,2-dihydro-[1,2,4]triazolo[4,3-a]quinolin-2-ium bromide 4

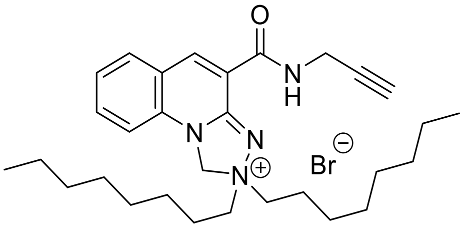

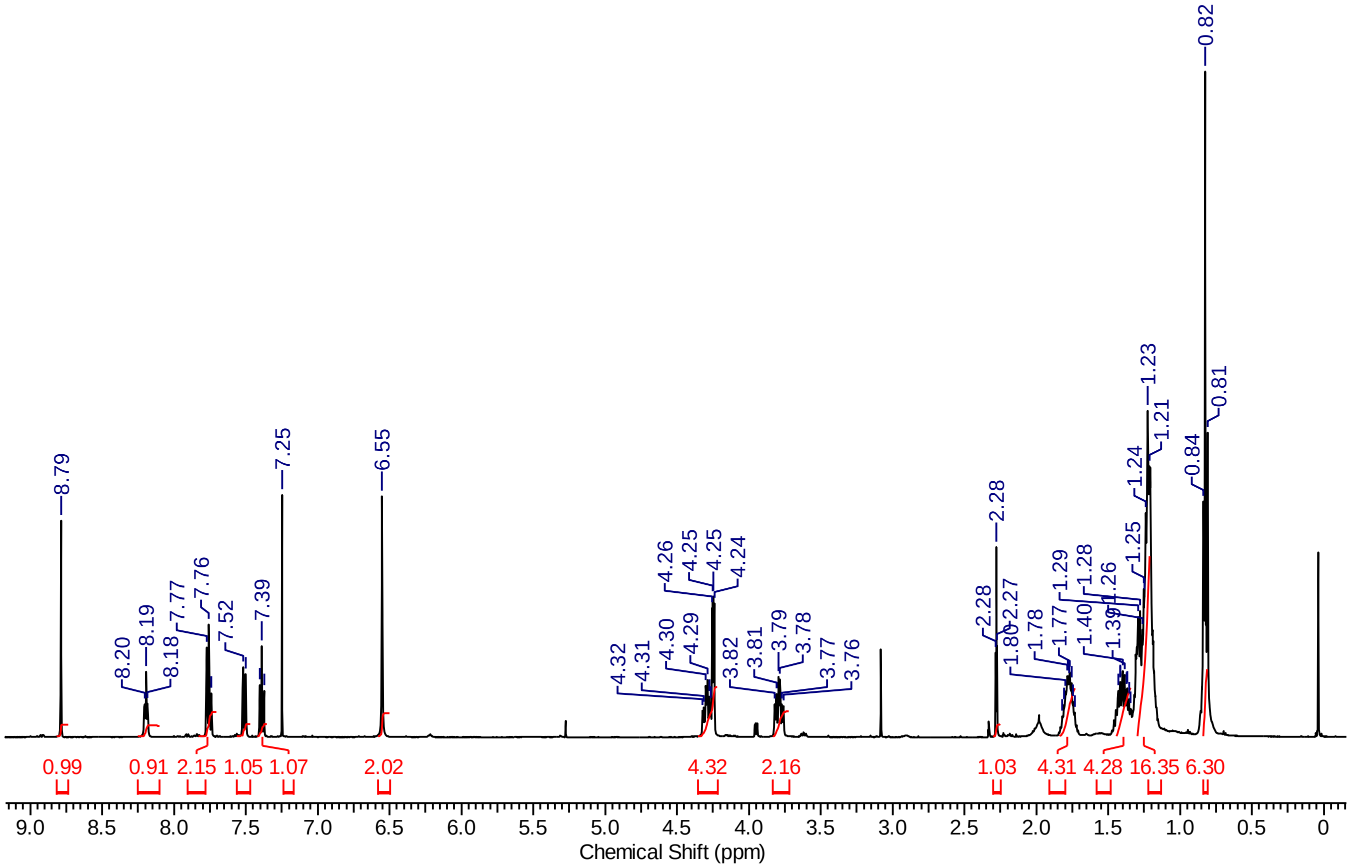

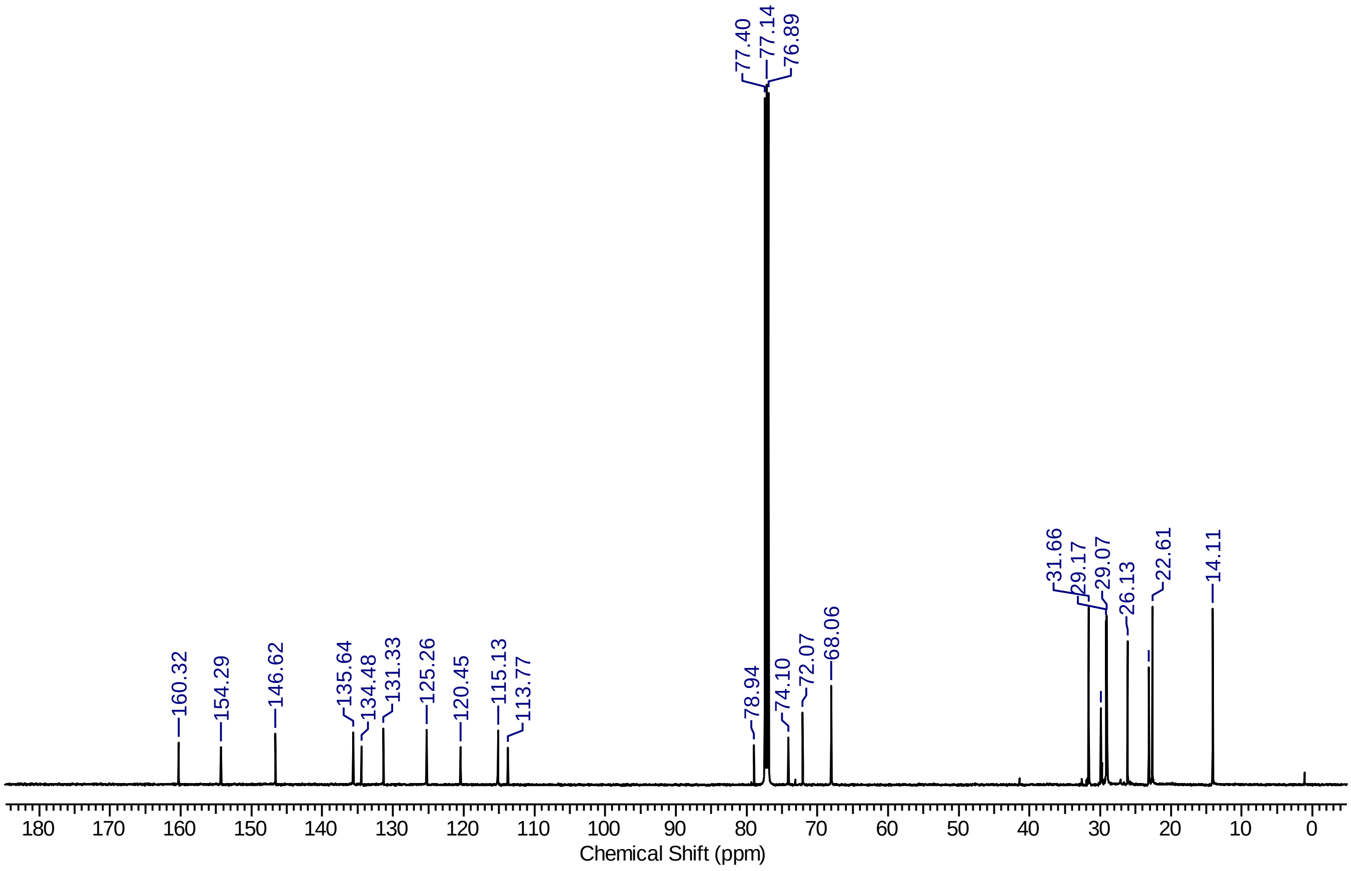
160.32
154.29
146.62
135.64
134.48
131.33
125.26
120.45
115.13
113.77
78.94
77.40
77.14
76.89
74.10
72.07
68.06
31.66
29.17
29.07
26.13
22.61
14.11
180
170
160
150
140
130
120
110
100
90
80
70
60
50
40
30
20
10
0
Chemical Shift (ppm)

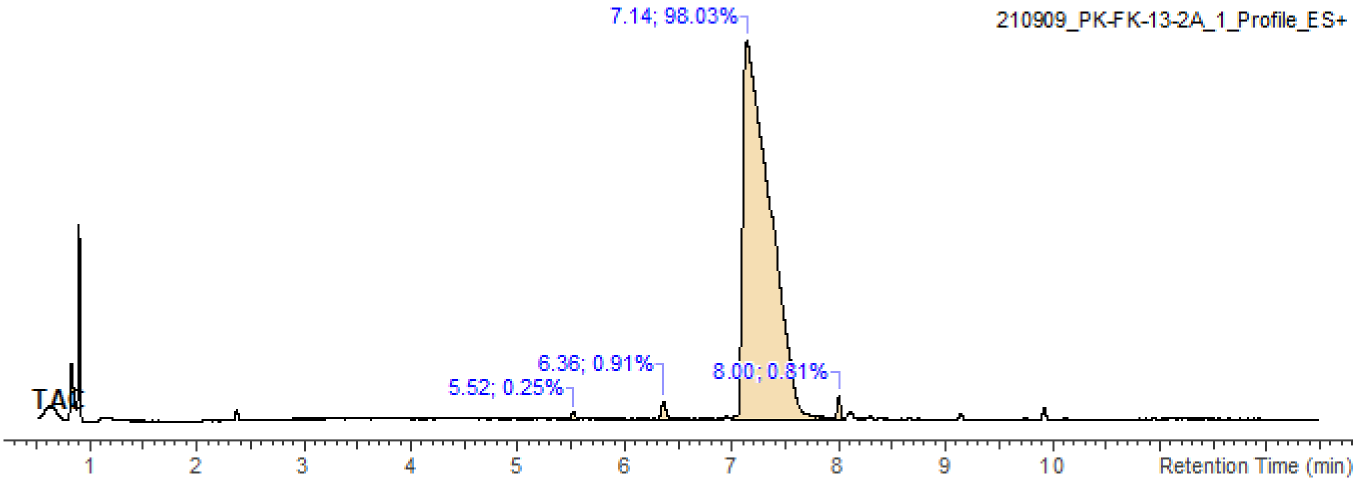
210909_PK-FK-13-2A_1_Profile_ES+
7.14; 98.03%
6.36; 0.91%
5.52; 0.25%
8.00; 0.81%
TAC
1
2
3
4
5
6
7
8
9
10
Retention Time (min)

Retention Time: 7.140

Ion Mode: ES+

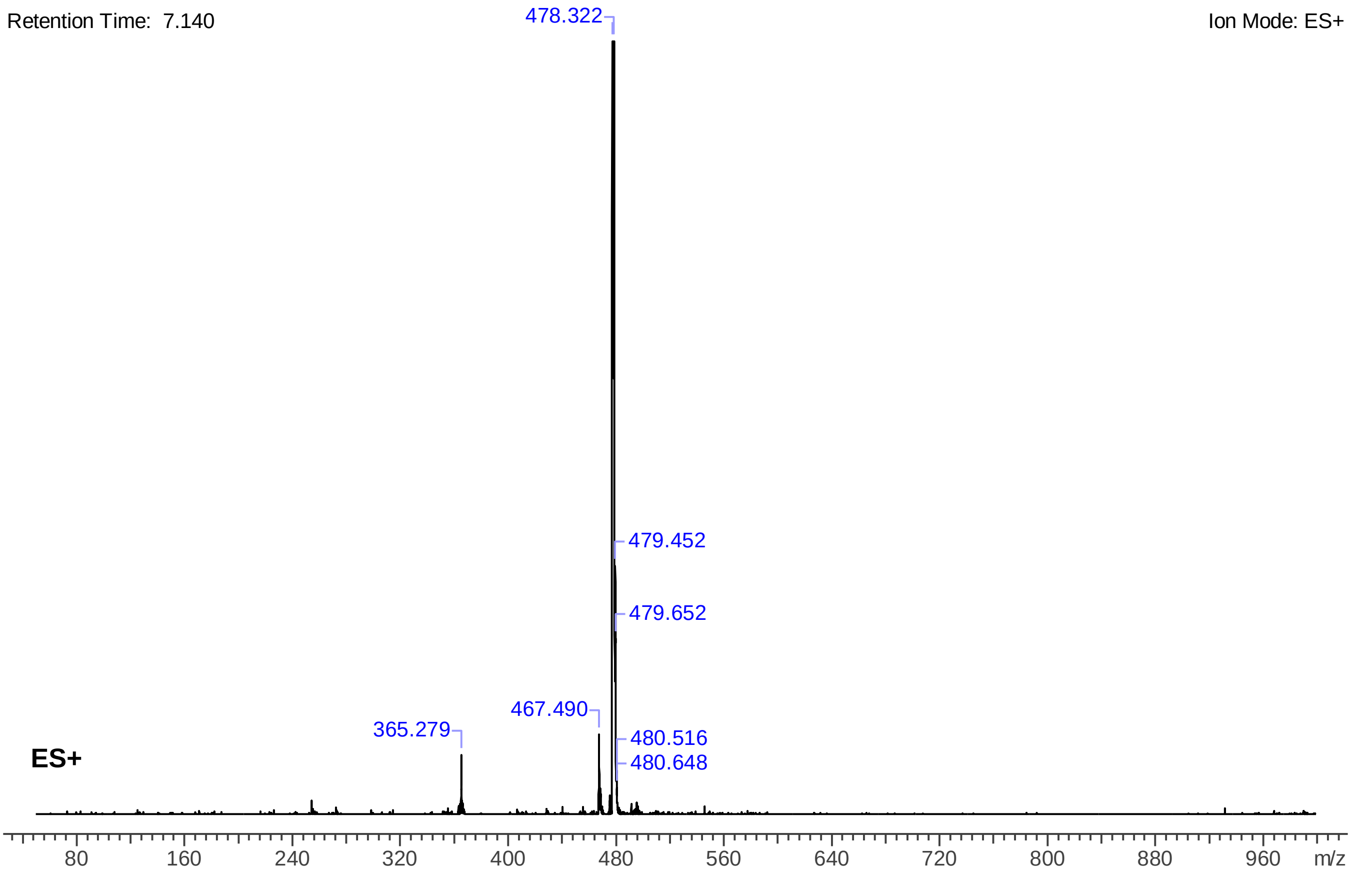

2,2-Diethyl-5,7-dimethyl-8-((prop-2-yn-1-yloxy)carbonyl)-2,3-dihydro-[1,2,4]triazolo[4,3-a]pyridin-2-ium bromide 5

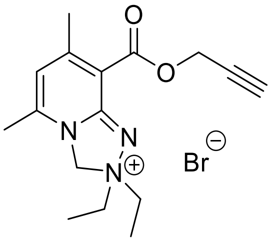

^{1}H NMR (300 MHz, $CDCl_3$)

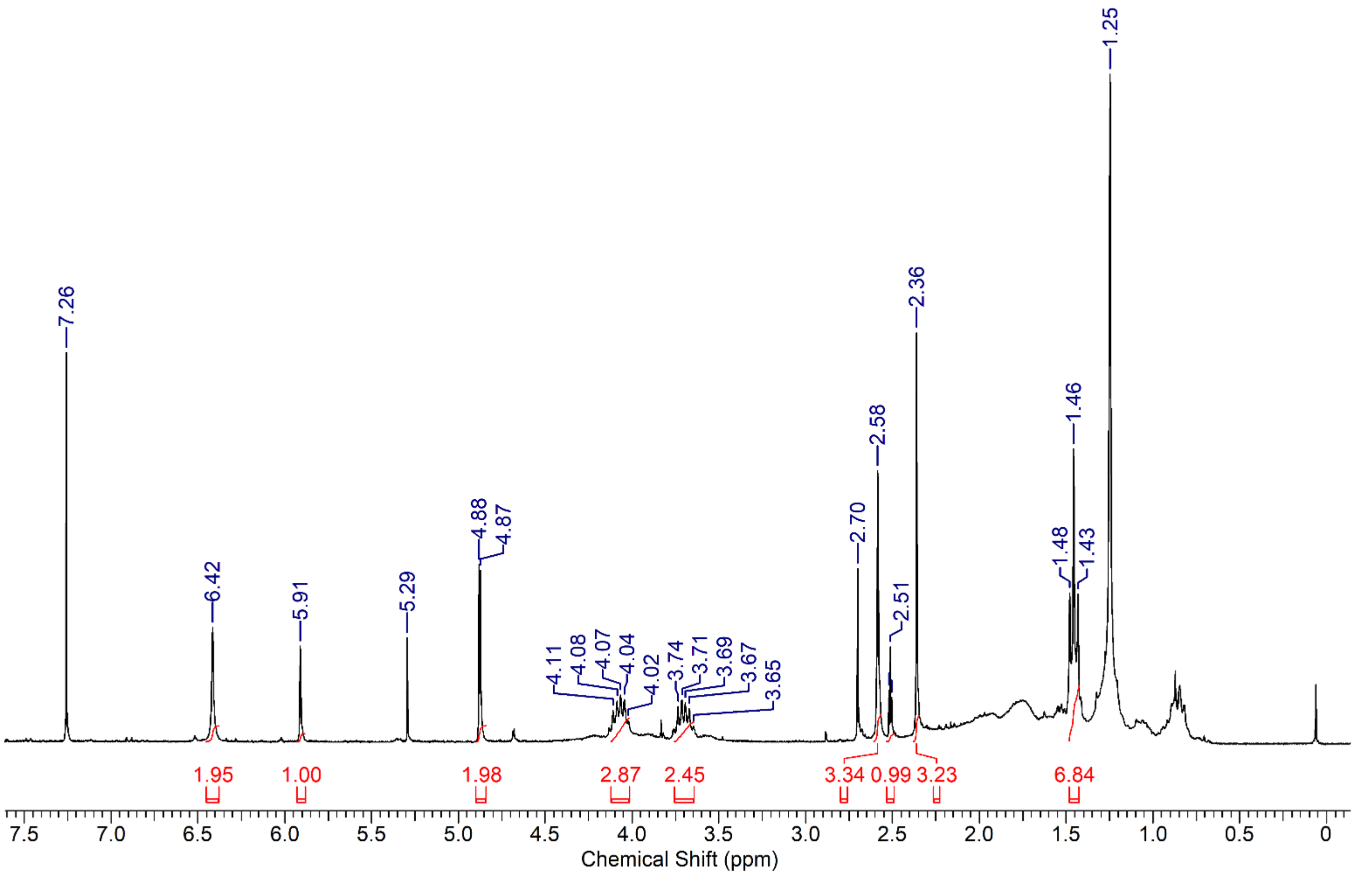

^{13}C NMR (75 MHz, $CDCl_3$)

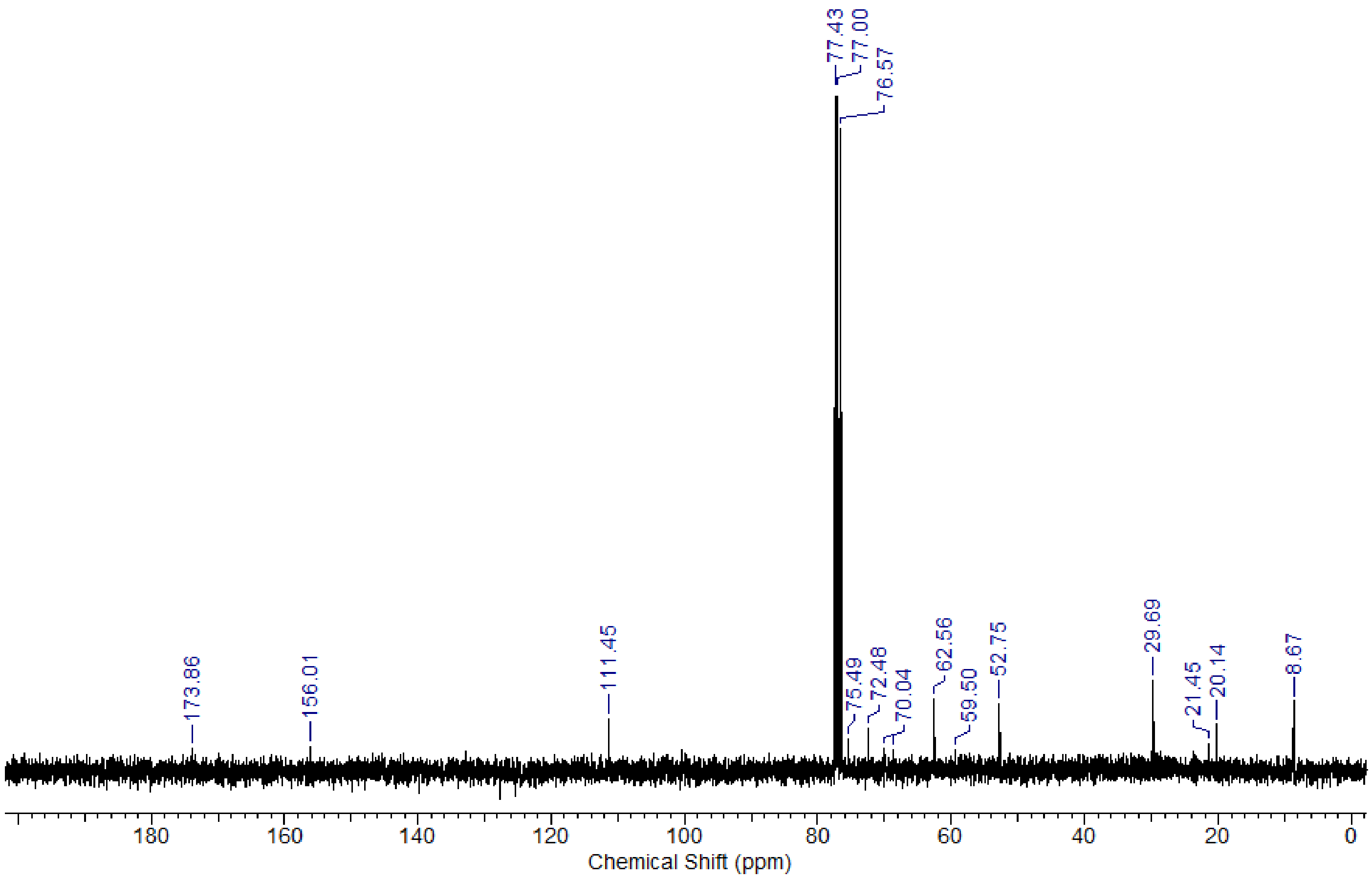

LC (DAD)

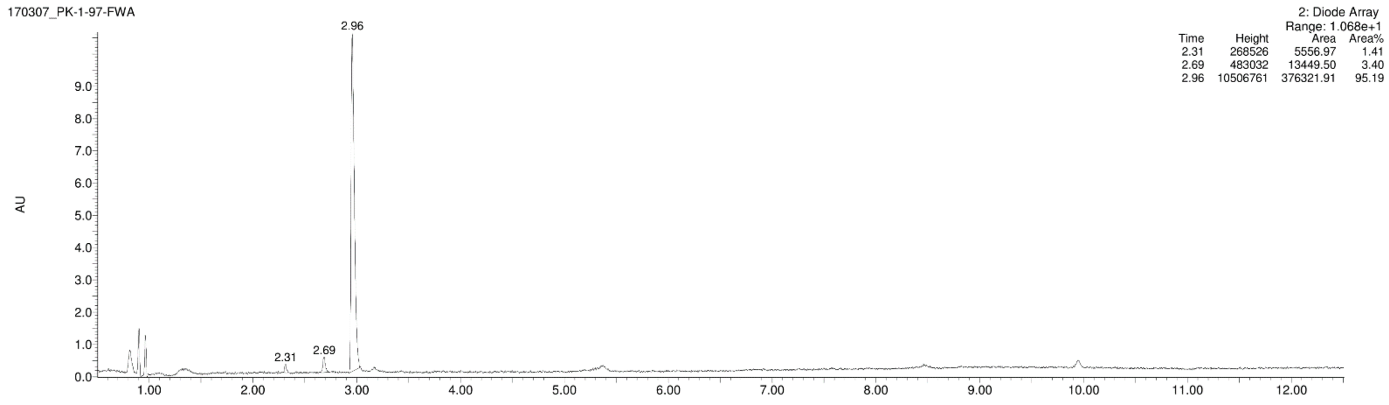

MS

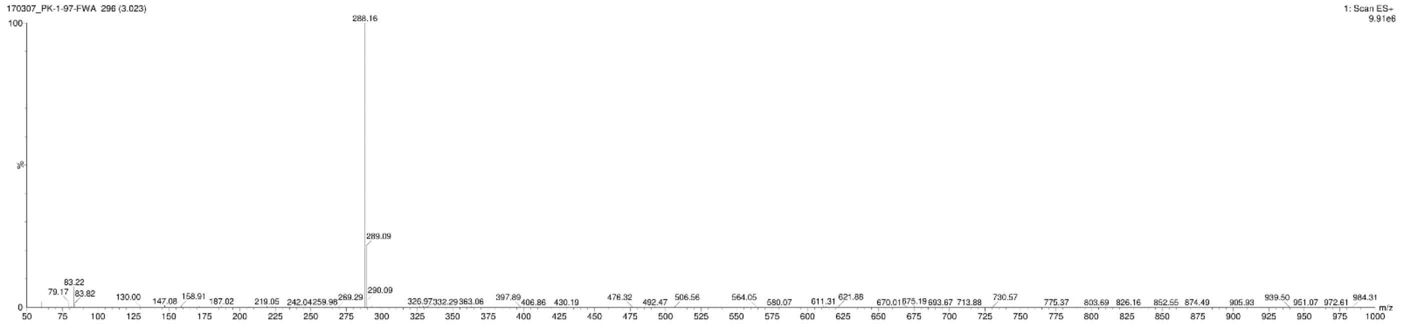

8-((5-Azidopentyl)carbamoyl)-2,2-diethyl-5,7-dimethyl-2,3-dihydro-[1,2,4]triazolo[4,3-a]pyridin-2-ium bromide 6

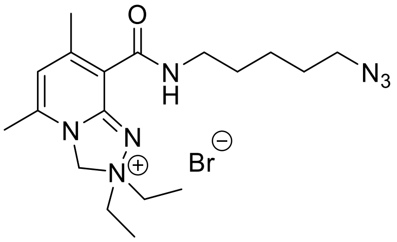

^{1}H NMR (500 MHz, $CDCl_3$)

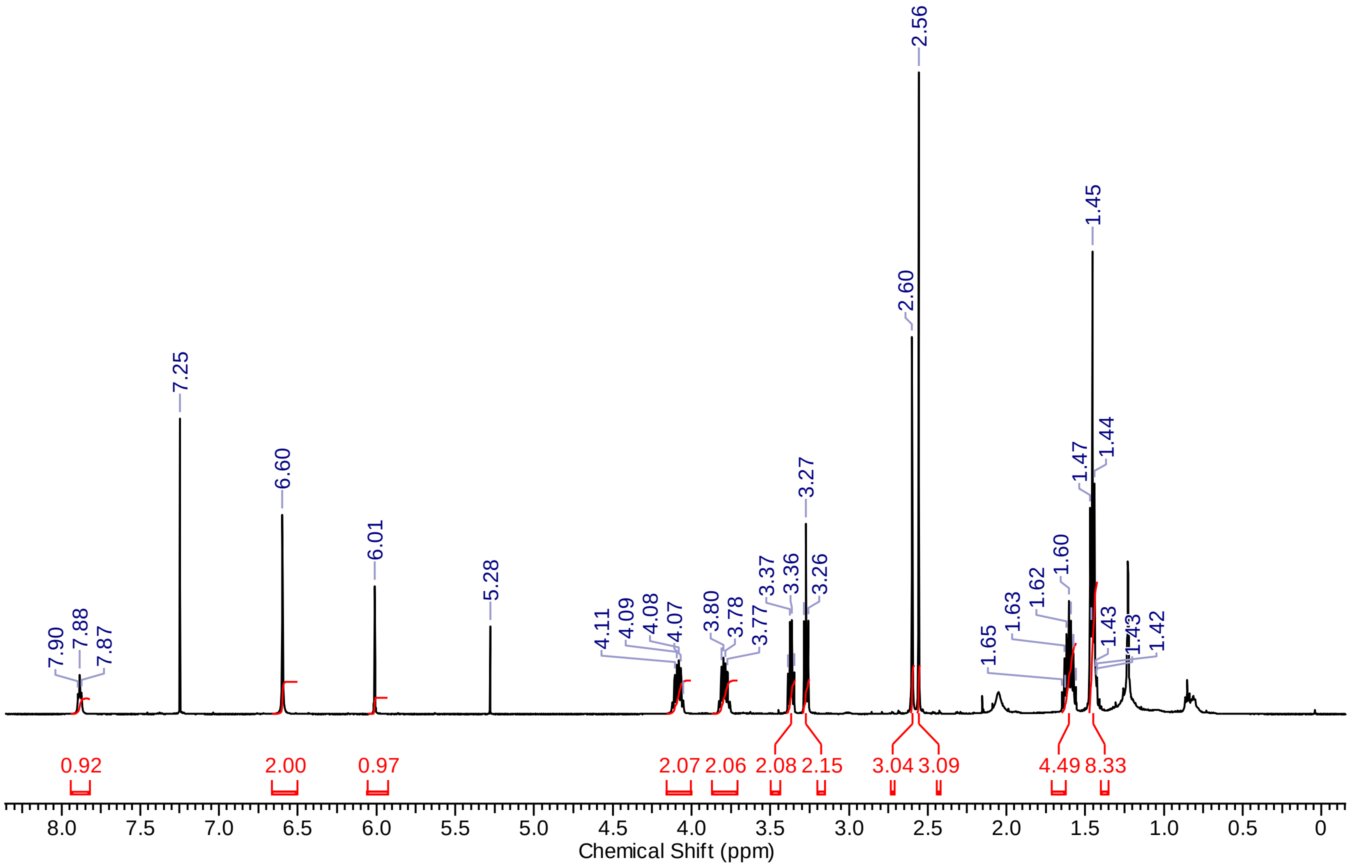

^{13}C NMR (126 MHz, $CDCl_3$)

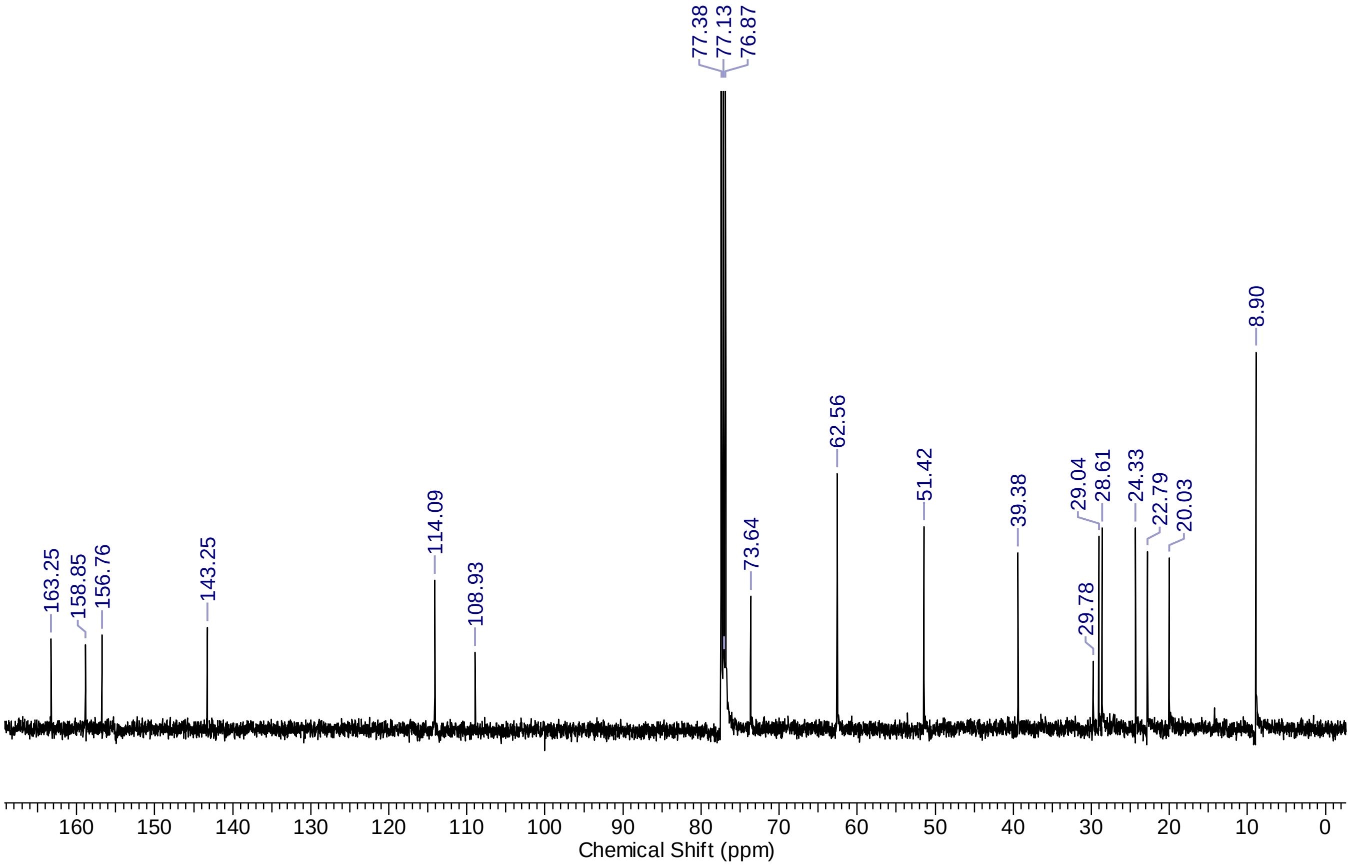

LC (DAD)

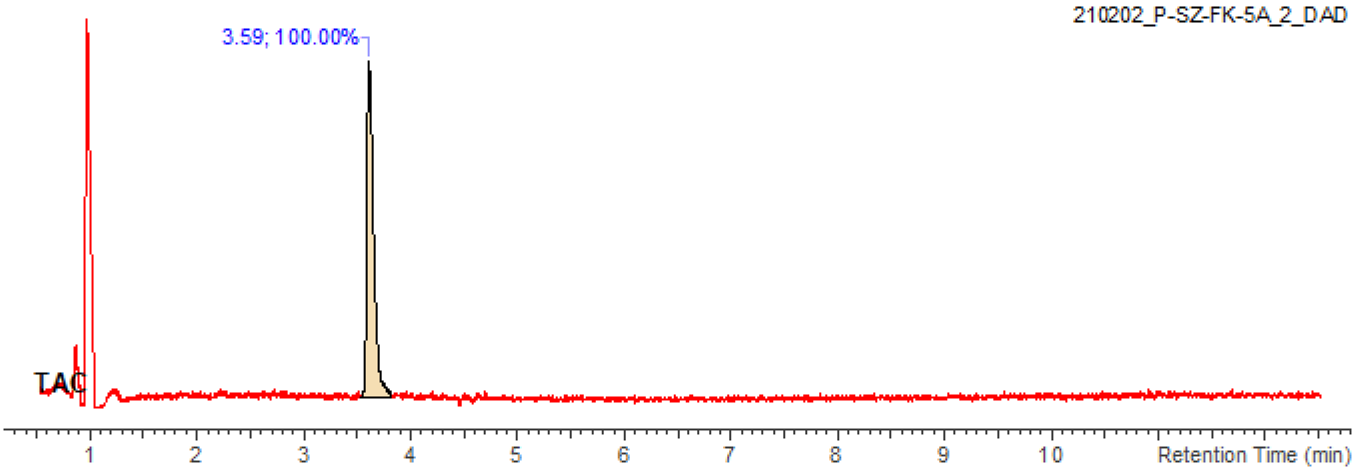

MS

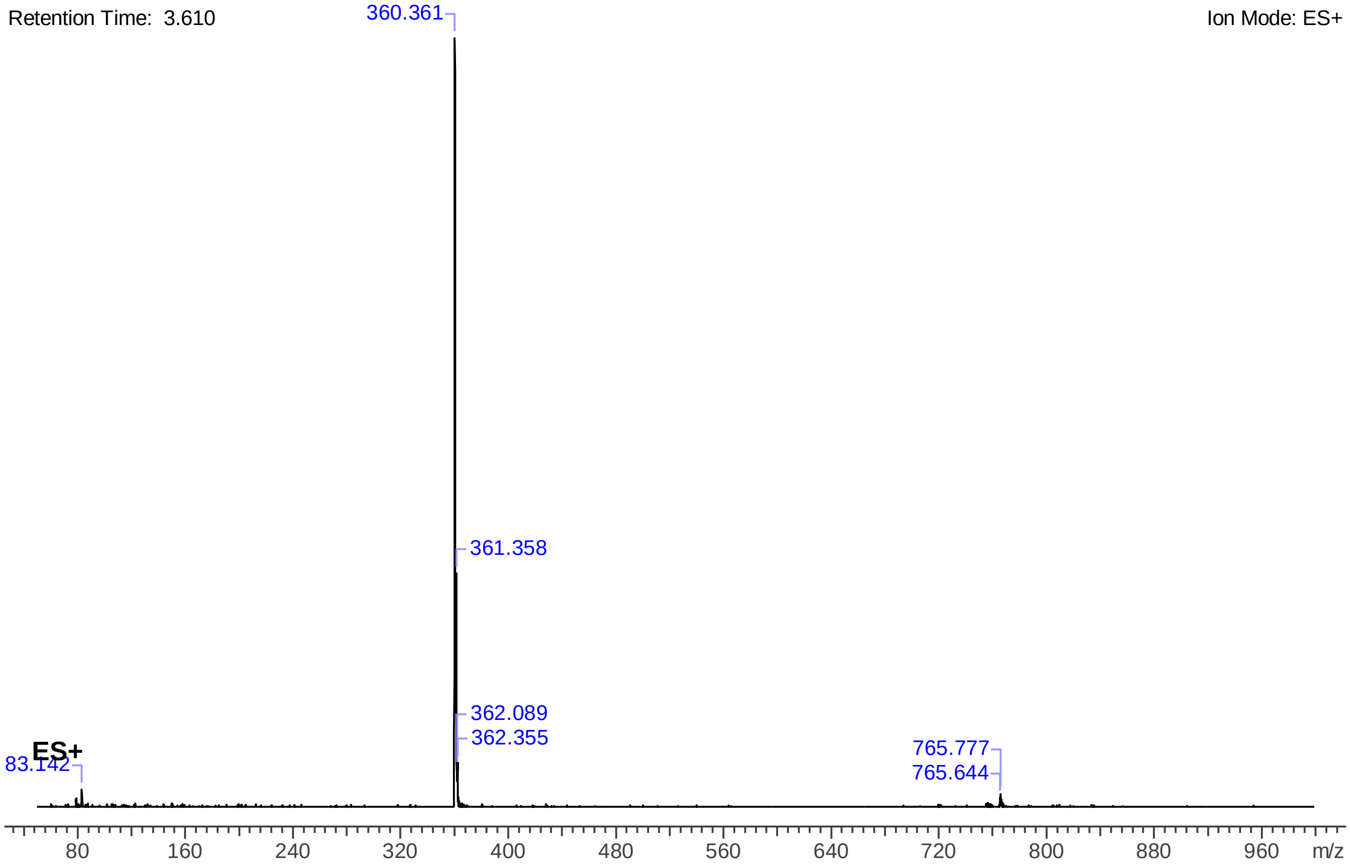

Retention Time: 3.610
Ion Mode: ES+
ES+
360.361
361.358
362.089
362.355
83.142
765.777
765.644
80
160
240
320
400
480
560
640
720
800
880
960
m/z

8-((5-Azidopentyl)carbamoyl)-5,7-dimethyl-2,2-dioctyl-2,3-dihydro-[1,2,4]triazolo[4,3-a]pyridin-2-ium bromide 7

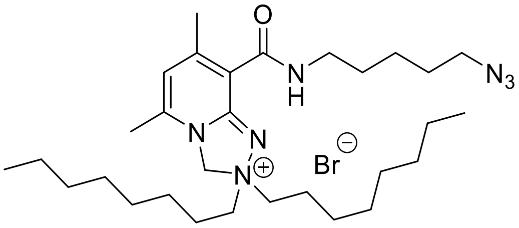

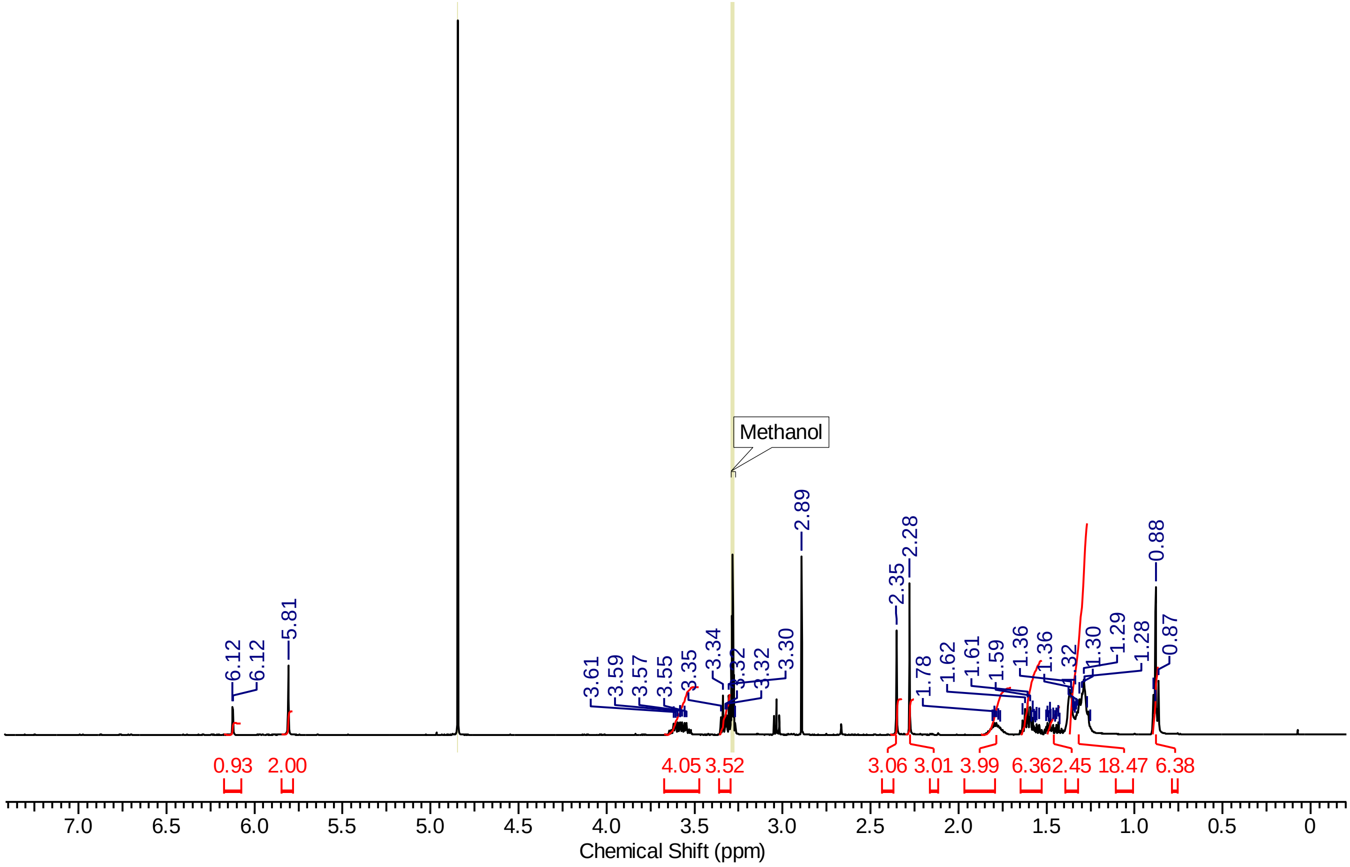

LC (DAD)

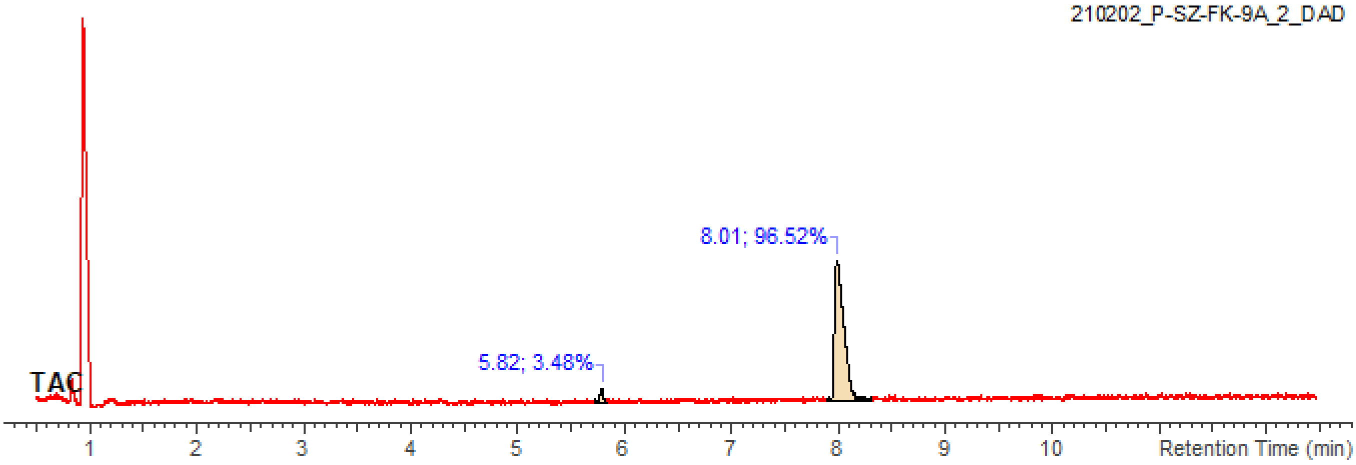

MS

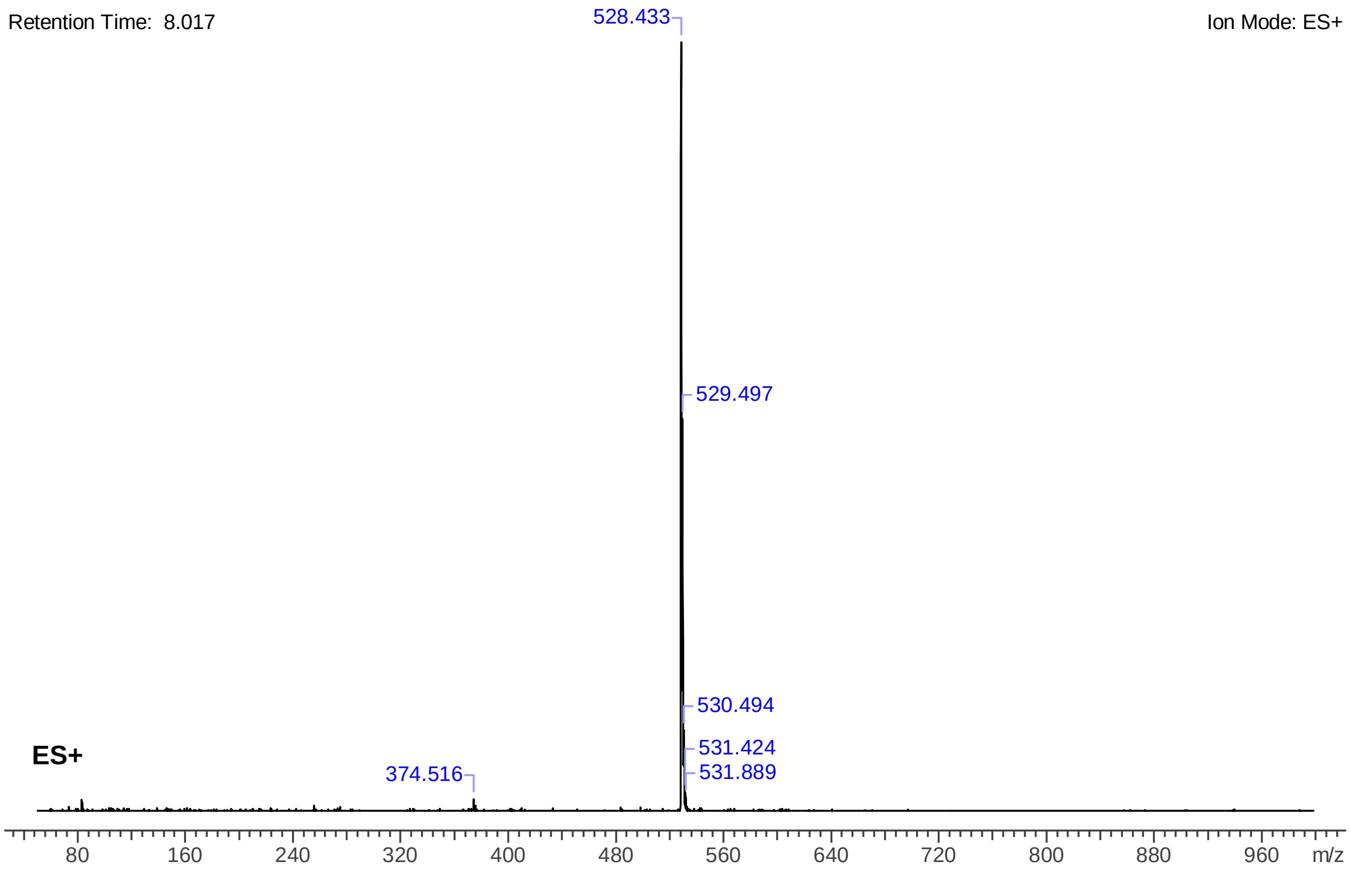

Retention Time: 8.017
Ion Mode: ES+
528.433
529.497
530.494
531.424
531.889
374.516
ES+
80
160
240
320
400
480
560
640
720
800
880
960
m/z

4-((5-Azidopentyl)carbamoyl)-2,2-diethyl-1,2-dihydro-[1,2,4]triazolo[4,3-a]quinolin-2-ium bromide 8

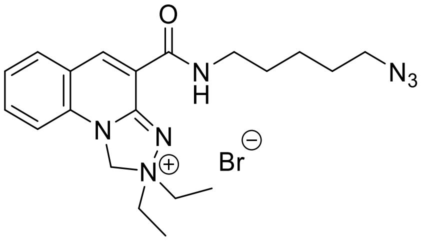

^{1}H NMR (500 MHz, $CDCl_3$)

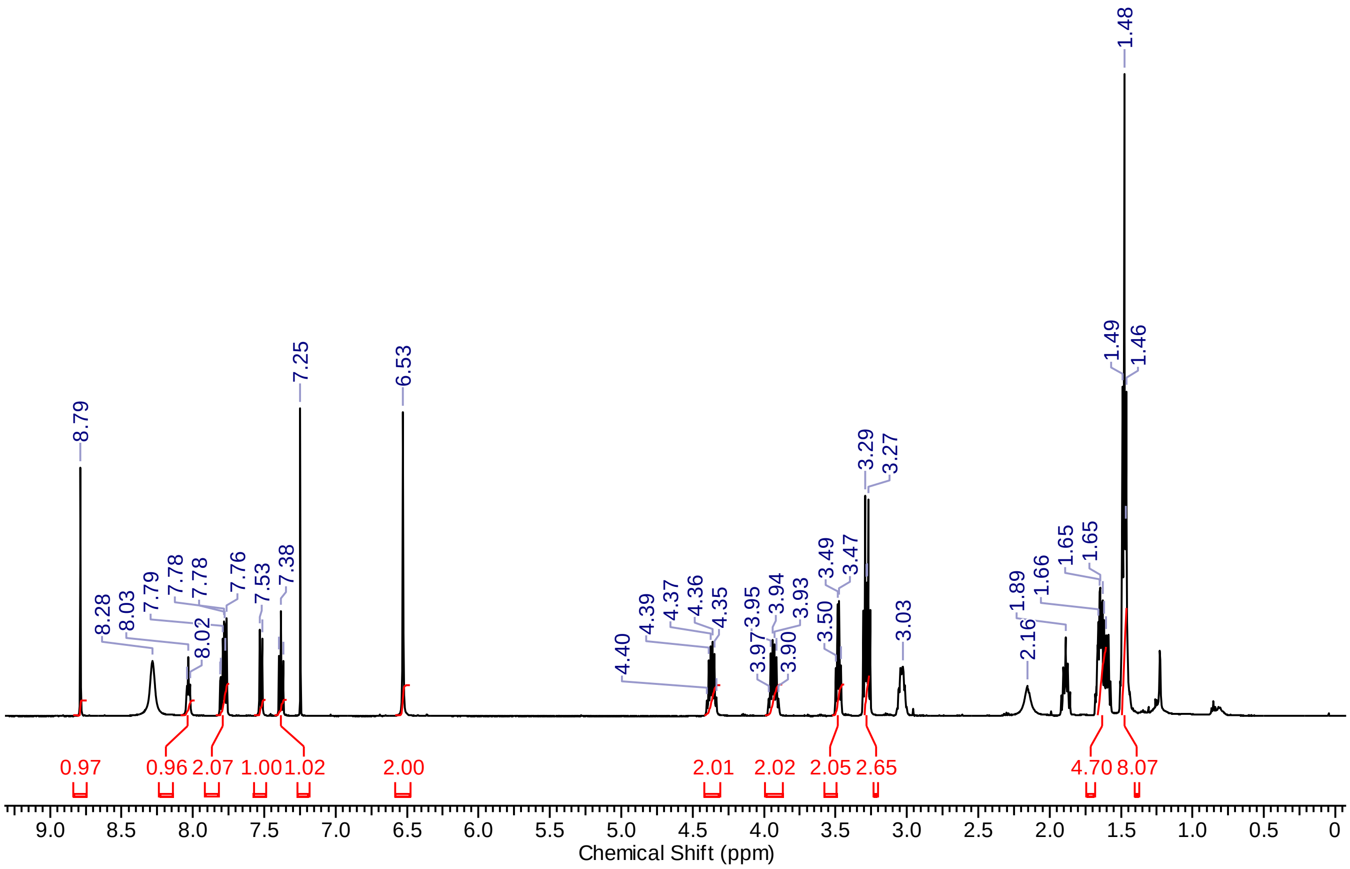

^{13}C NMR (126 MHz, $CDCl_3$)

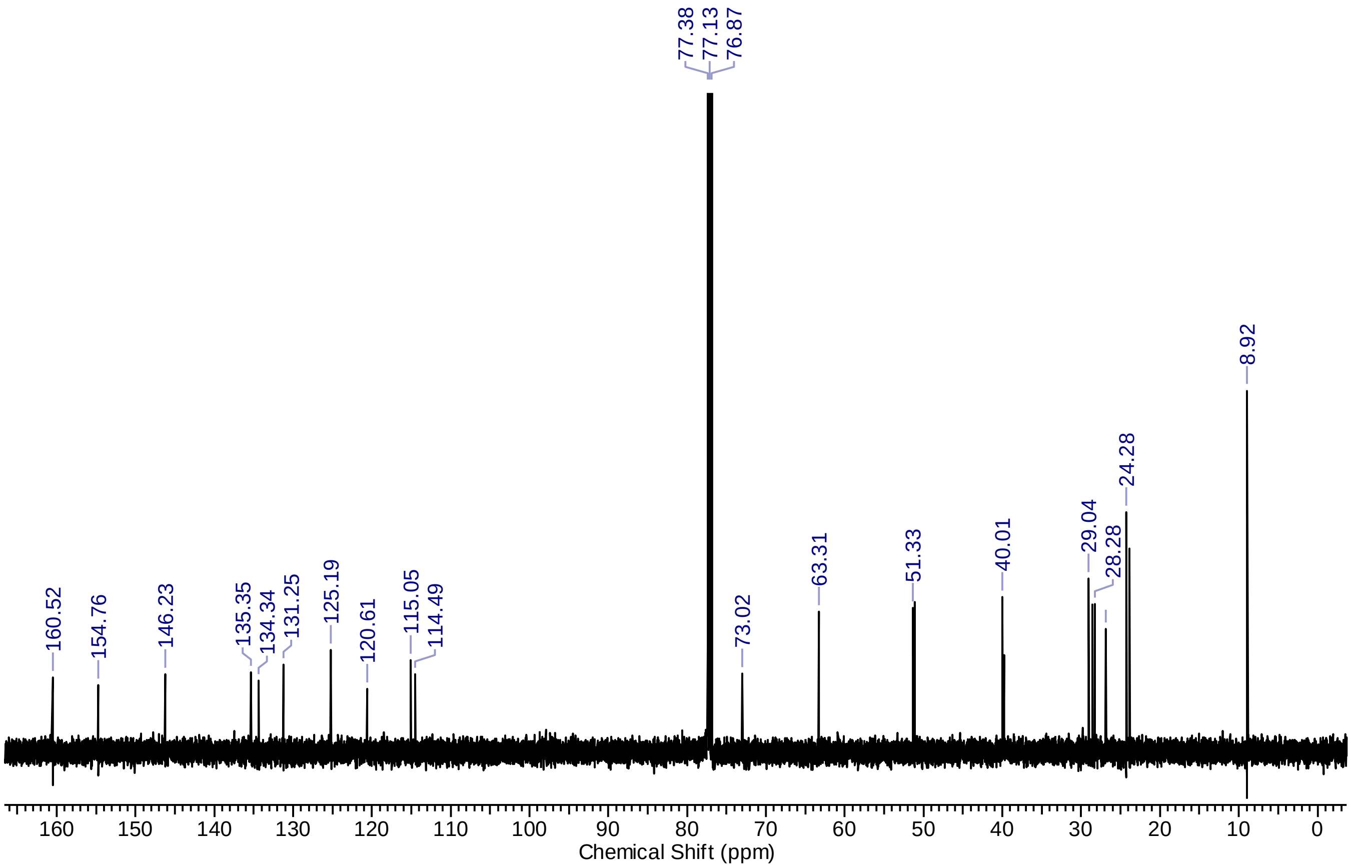
77.38
77.13
76.87
160.52
154.76
146.23
135.35
134.34
131.25
125.19
120.61
115.05
114.49
73.02
63.31
51.33
40.01
29.04
28.28
24.28
8.92
160 150 140 130 120 110 100 90 80 70 60 50 40 30 20 10 0
Chemical Shift (ppm)

LC (DAD)

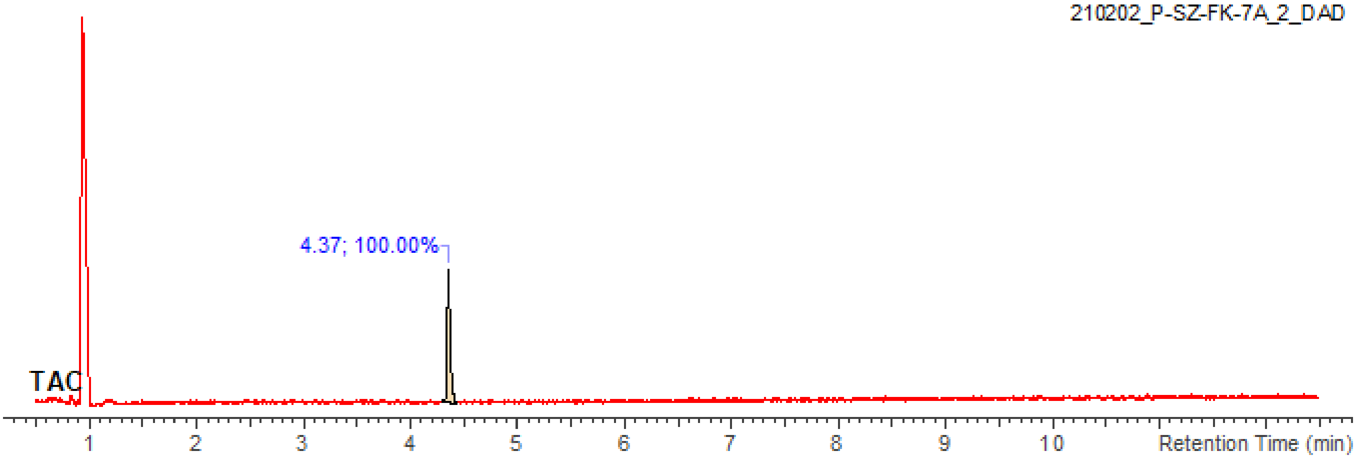
210202_P-SZ-FK-7A_2_DAD
4.37; 100.00%
TAC
1 2 3 4 5 6 7 8 9 10
Retention Time (min)

MS

Retention Time: 4.396

Ion Mode: ES+

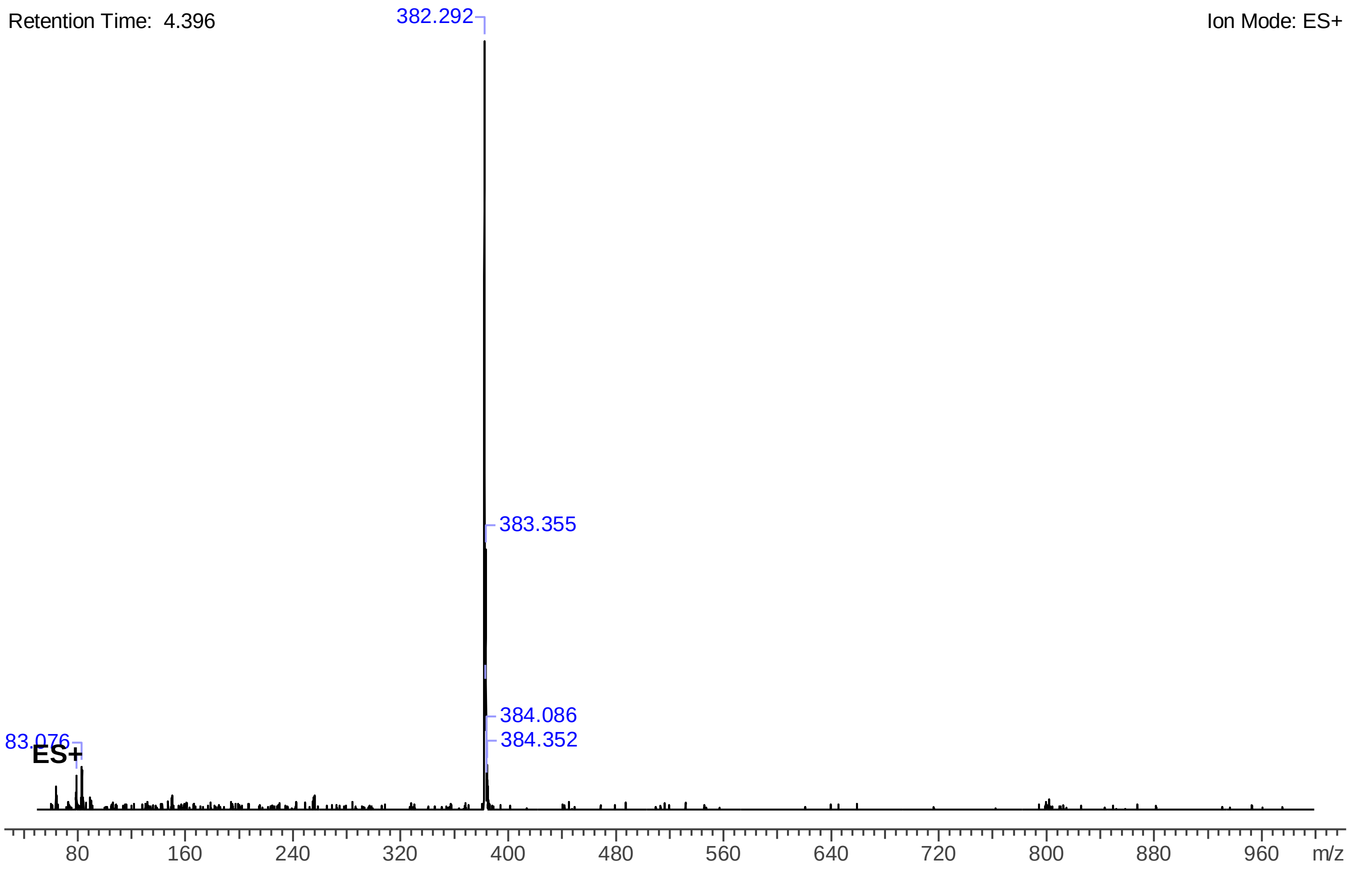

4-((5-Azidopentyl)carbamoyl)-2,2-dioctyl-1,2-dihydro-[1,2,4]triazolo[4,3-a]quinolin-2-ium bromide 9

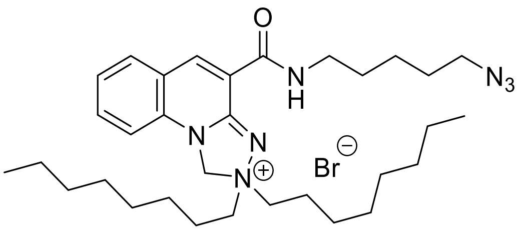

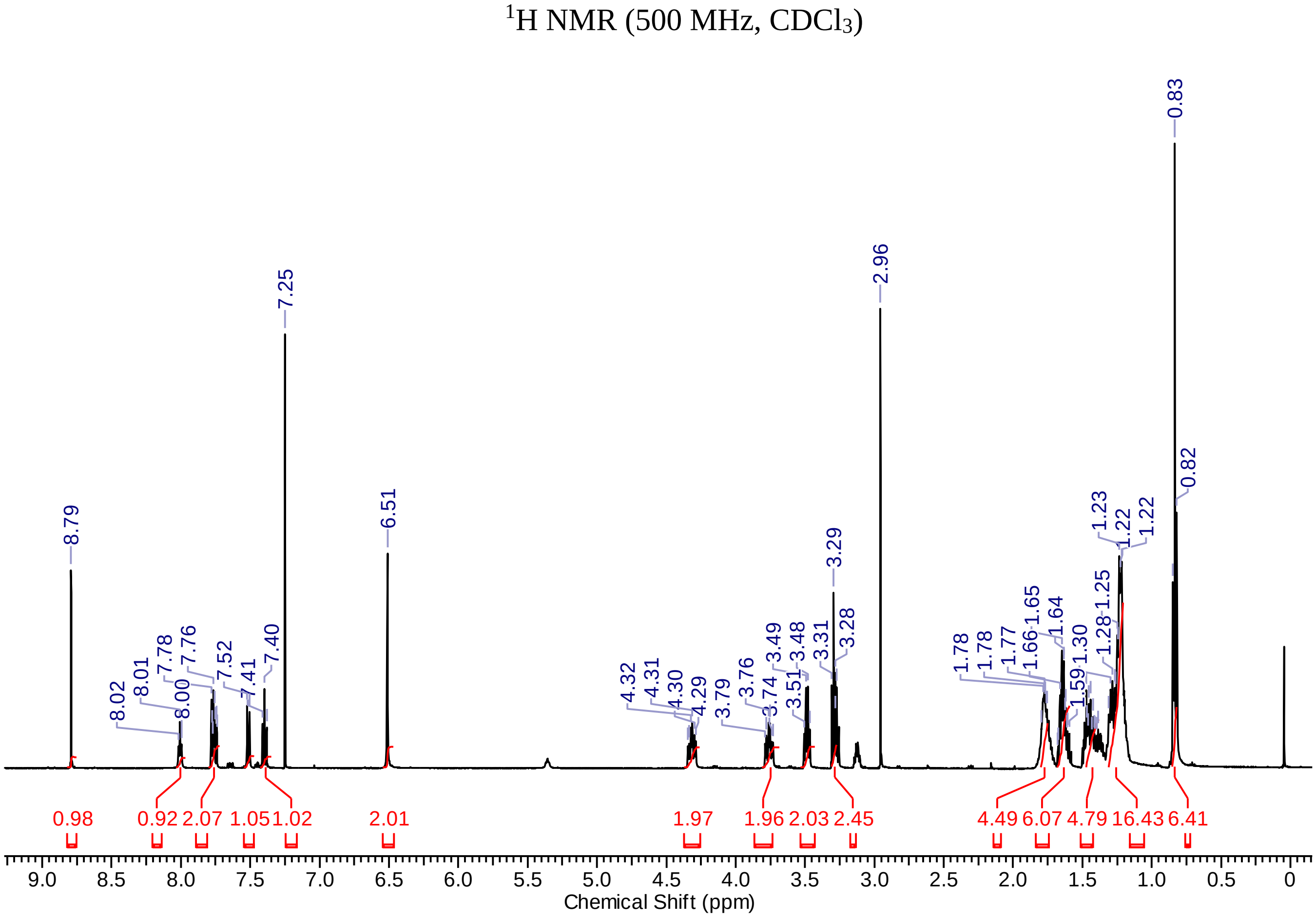

^{13}C NMR (126 MHz, $CDCl_3$)

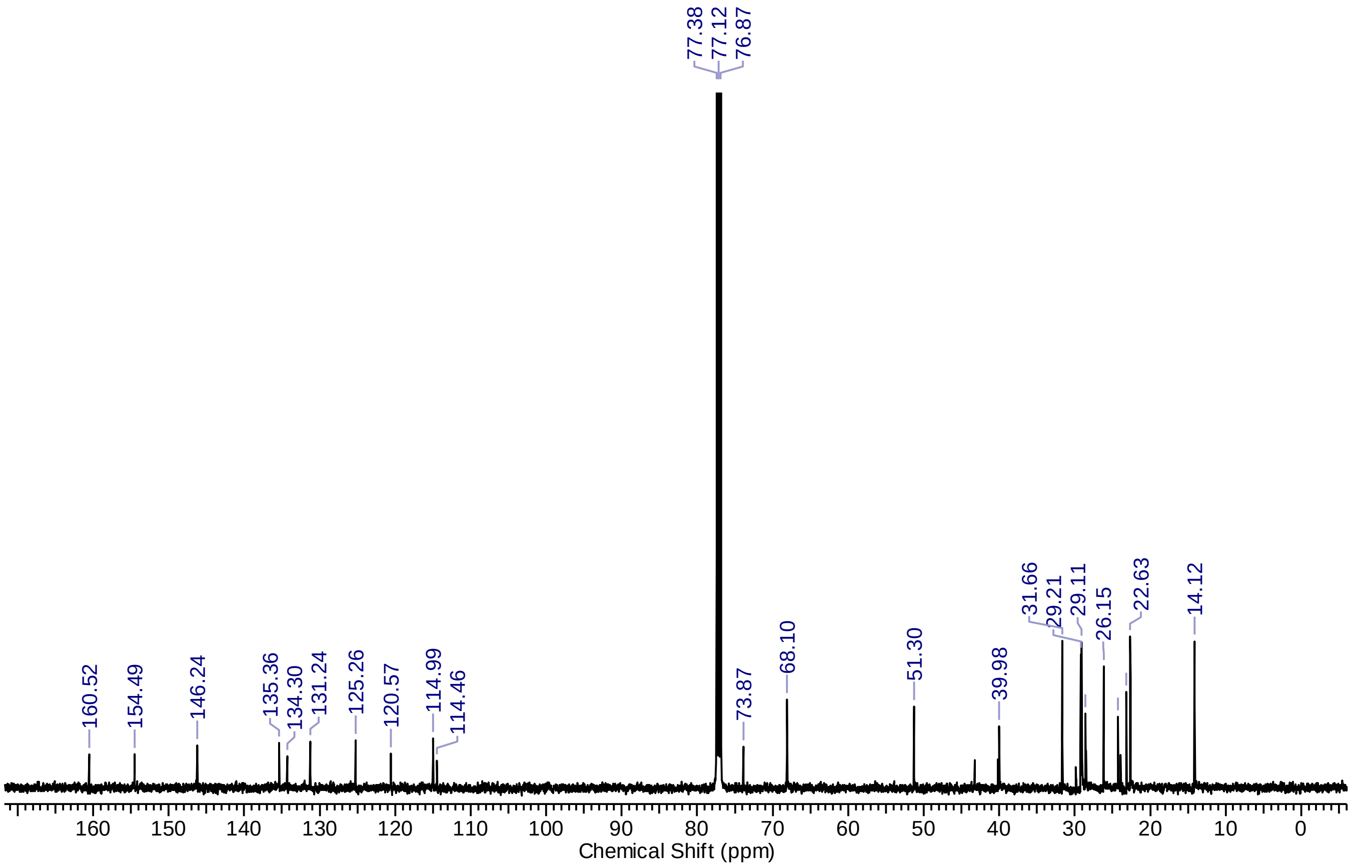

LC (DAD)

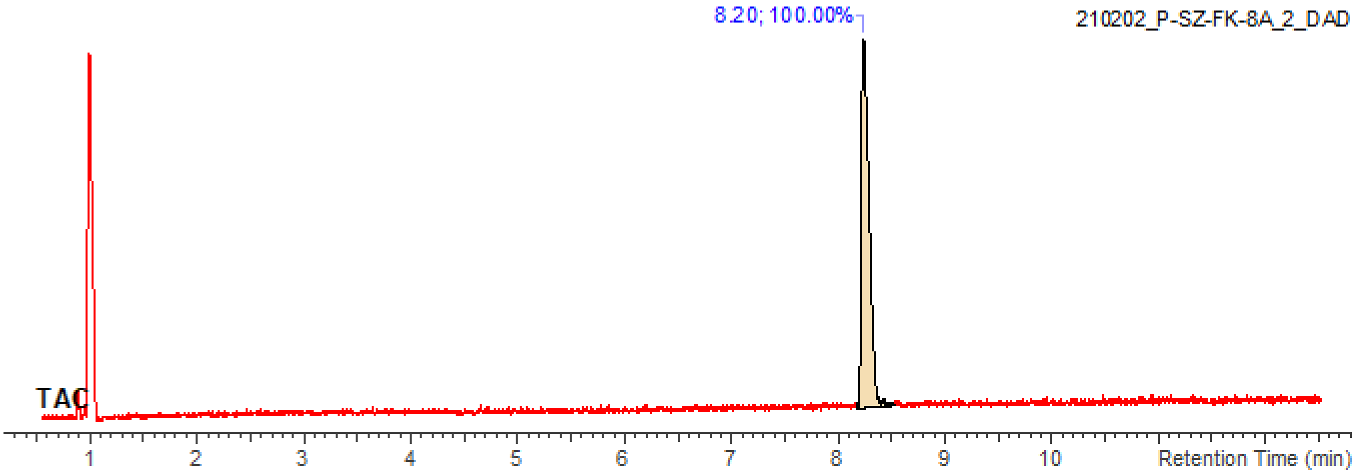

MS

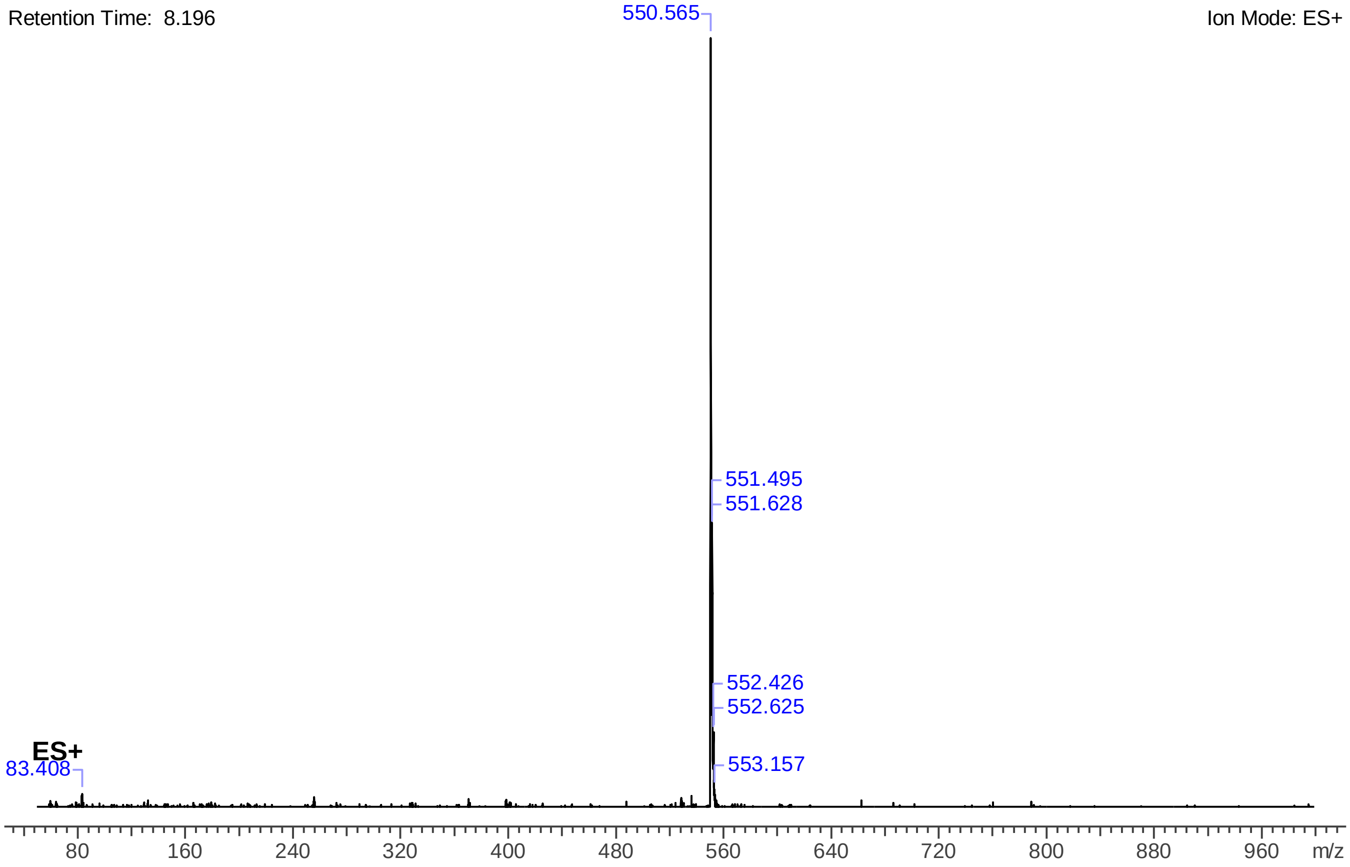
Retention Time: 8.196
Ion Mode: ES+
550.565
551.495
551.628
552.426
552.625
553.157
ES+
83.408
80
160
240
320
400
480
560
640
720
800
880
960
m/z

8-((7-Azidoheptyl)carbamoyl)-2,2-diethyl-5,7-dimethyl-2,3-dihydro-[1,2,4]triazolo[4,3-a]pyridin-2-ium bromide 10

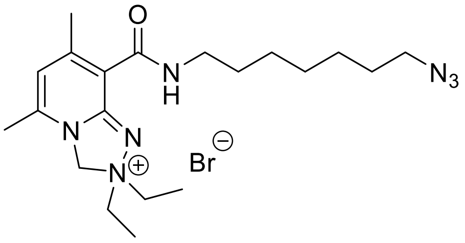

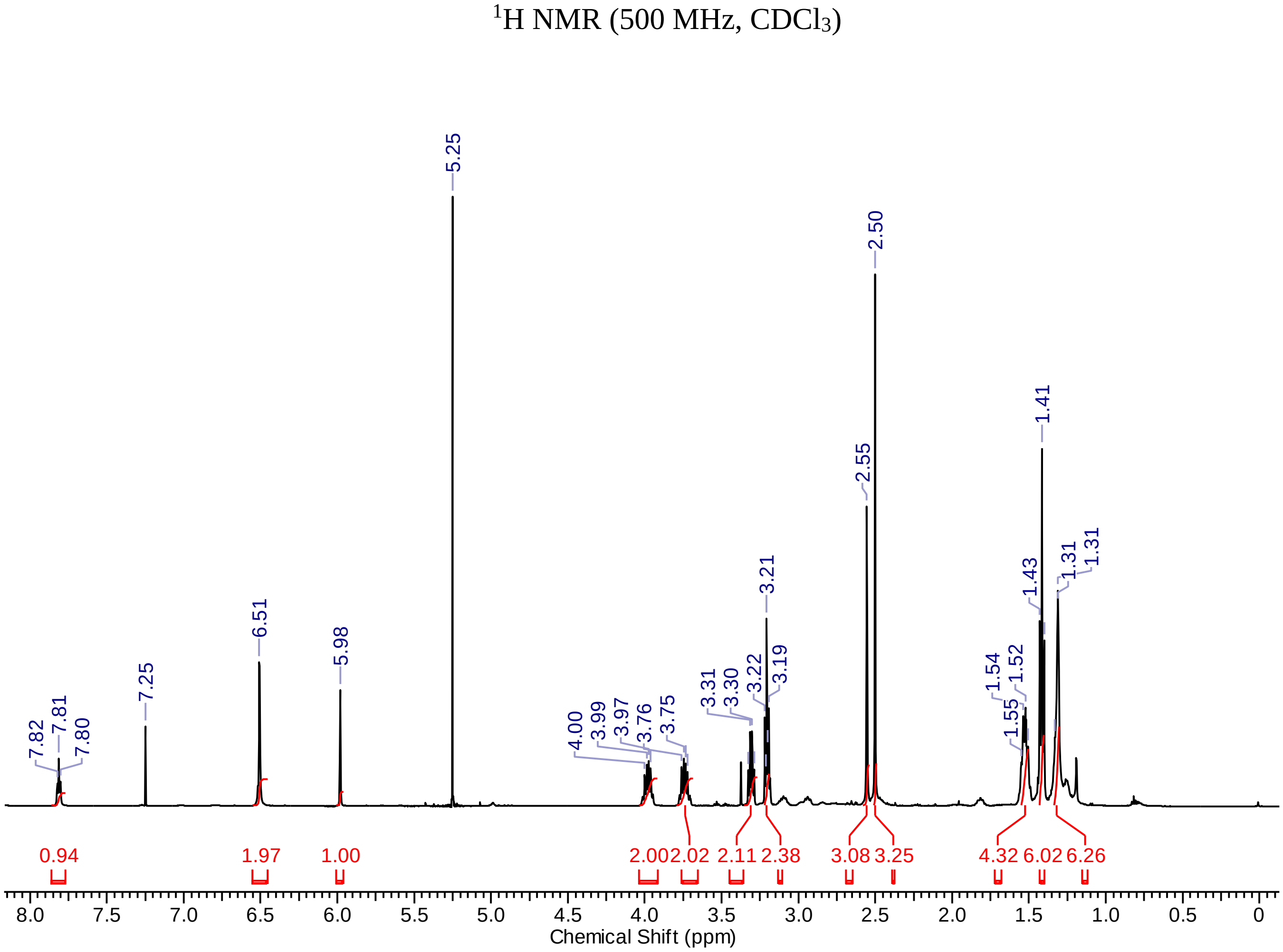

^{13}C NMR (126 MHz, $CDCl_3$)

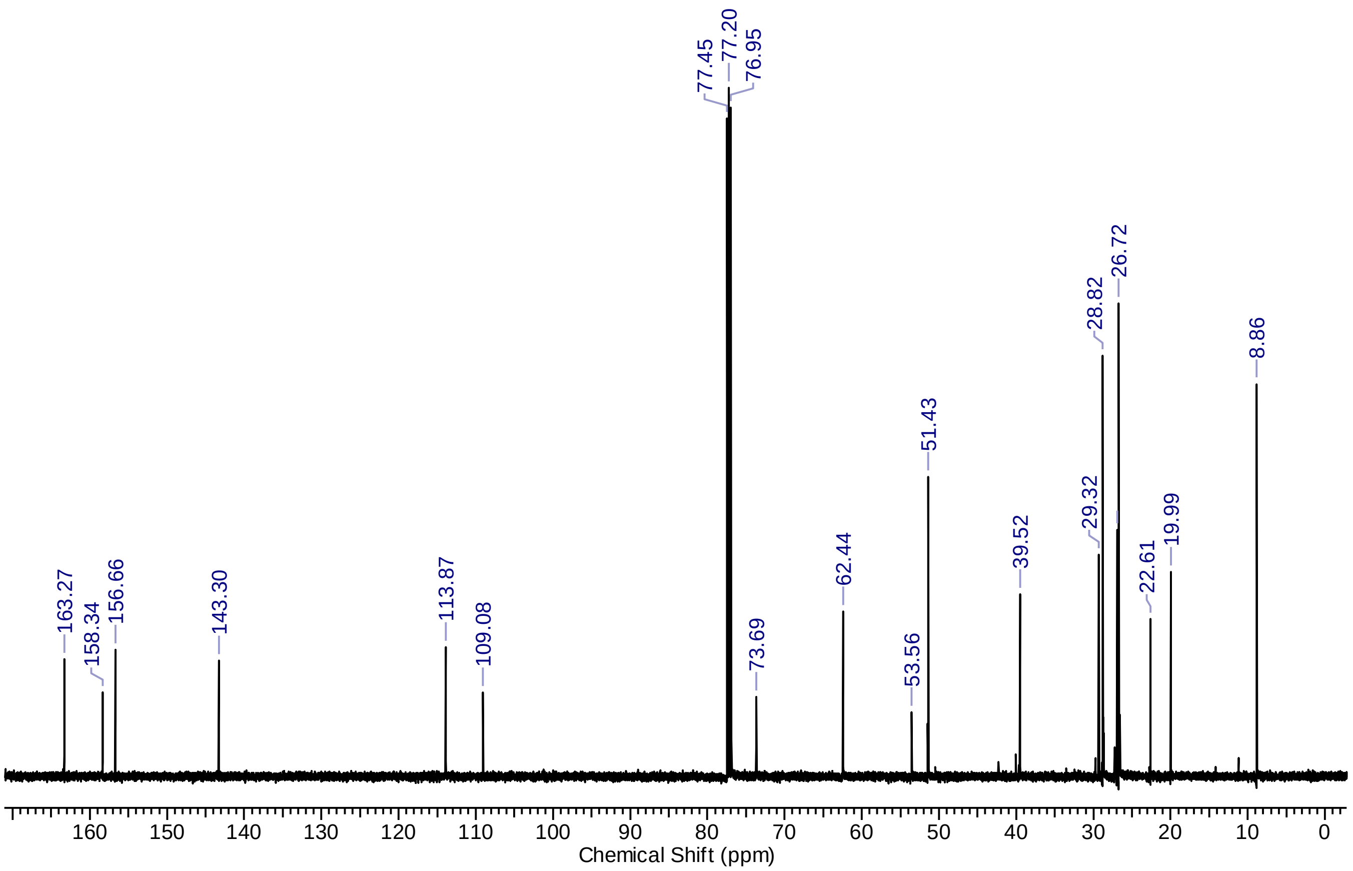

LC (DAD)

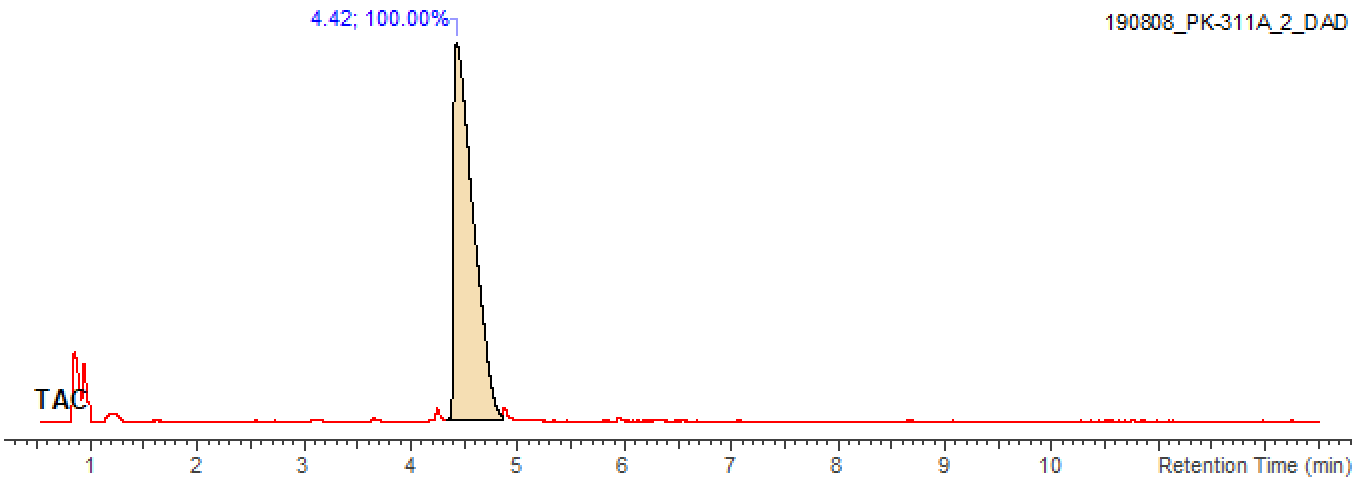

MS

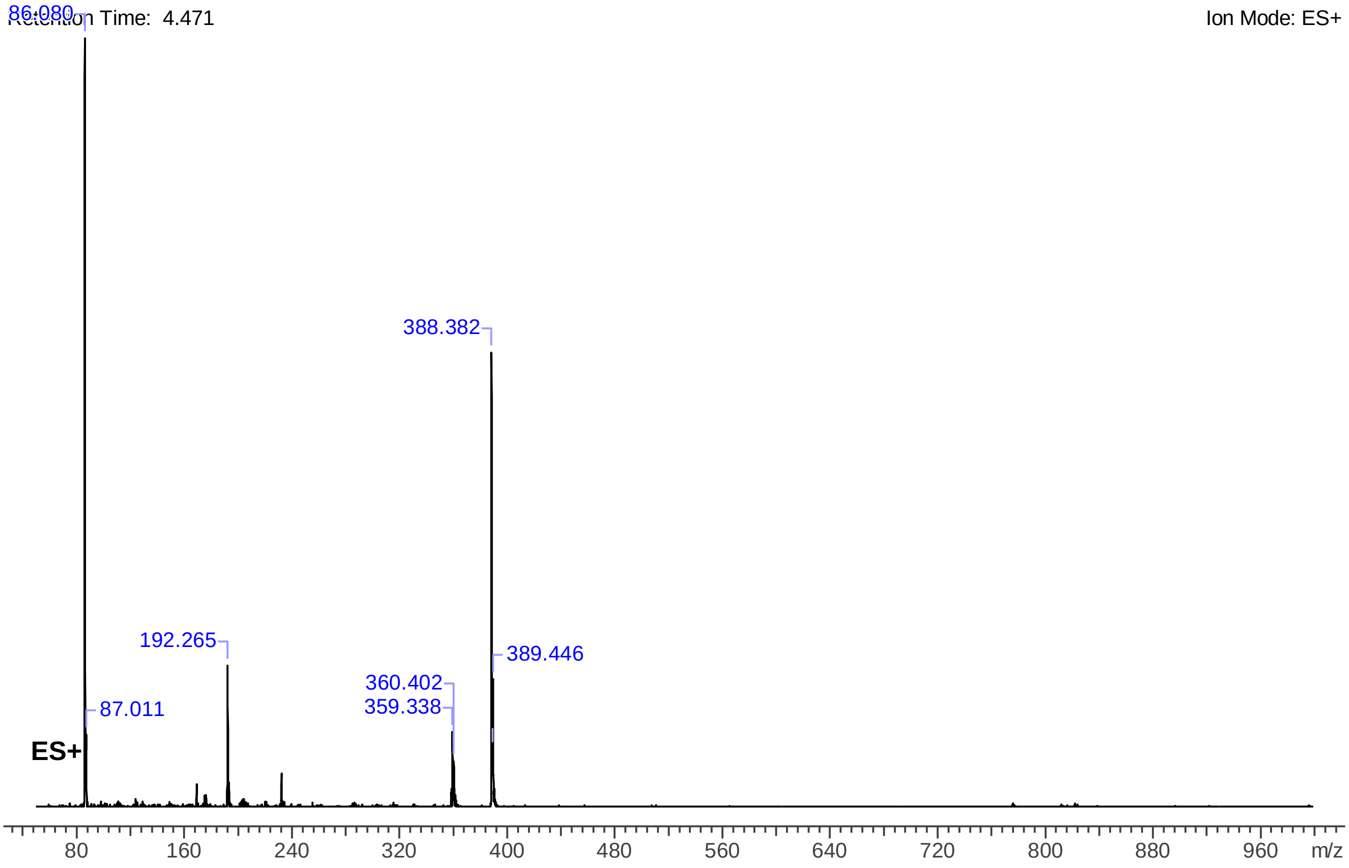

Retention Time: 4.471
Ion Mode: ES+
86.080
87.011
192.265
360.402
359.338
388.382
389.446
ES+
80
160
240
320
400
480
560
640
720
800
880
960
m/z

3. UV-VIS absorption and emission spectra used for quantum yield estimation

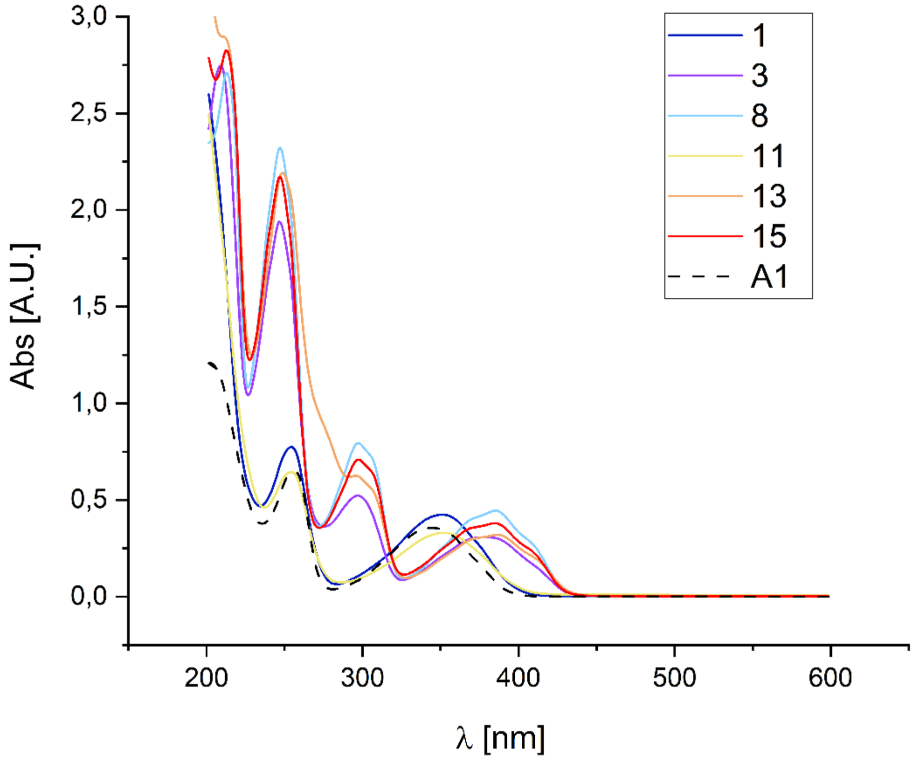

Figure S1: UV-VIS absorption spectra for $1 \cdot 10^{-4}$ mol/dm^3 aqueous solutions of compounds 1, 3, 8, 11, 13, 15 and A1

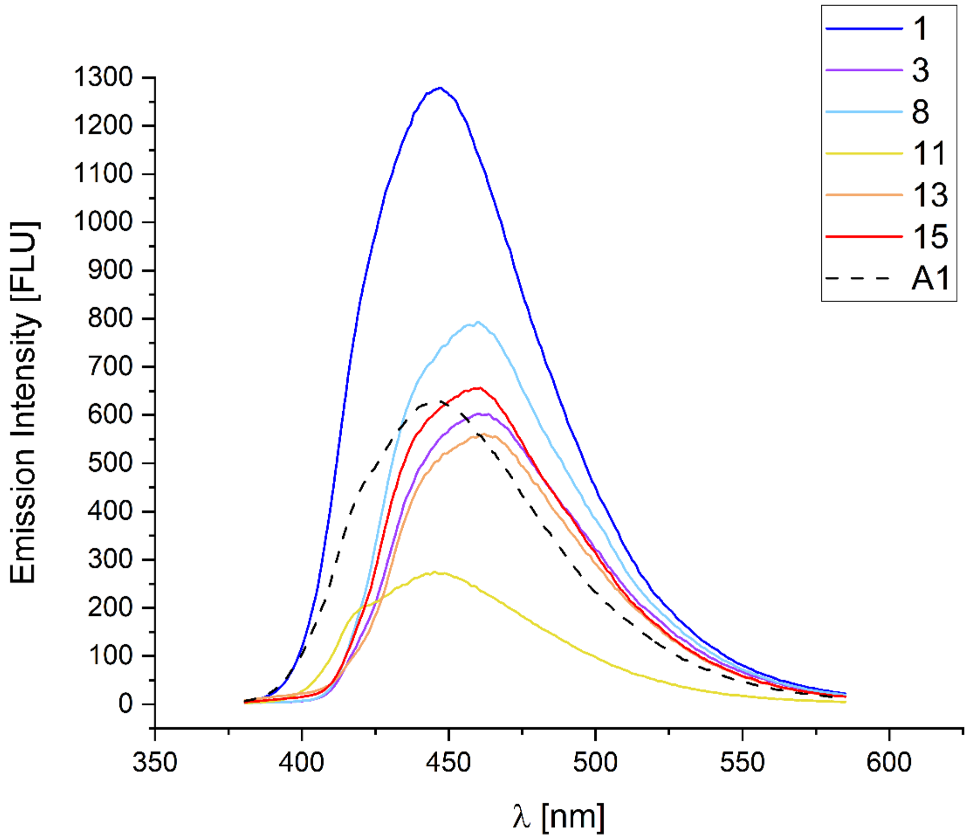

Figure S2: Non-normalized UV-VIS emissionn spectra, recorded at 365 nm excitation wavelength (5 nm excitation slit, 2.5 nm emission slit, 700V PMT voltage), for $1 \cdot 10^{-6}$ mol/dm^3 aqueous solutions of compounds 1, 3, 8, 11, 13, 15 and A1

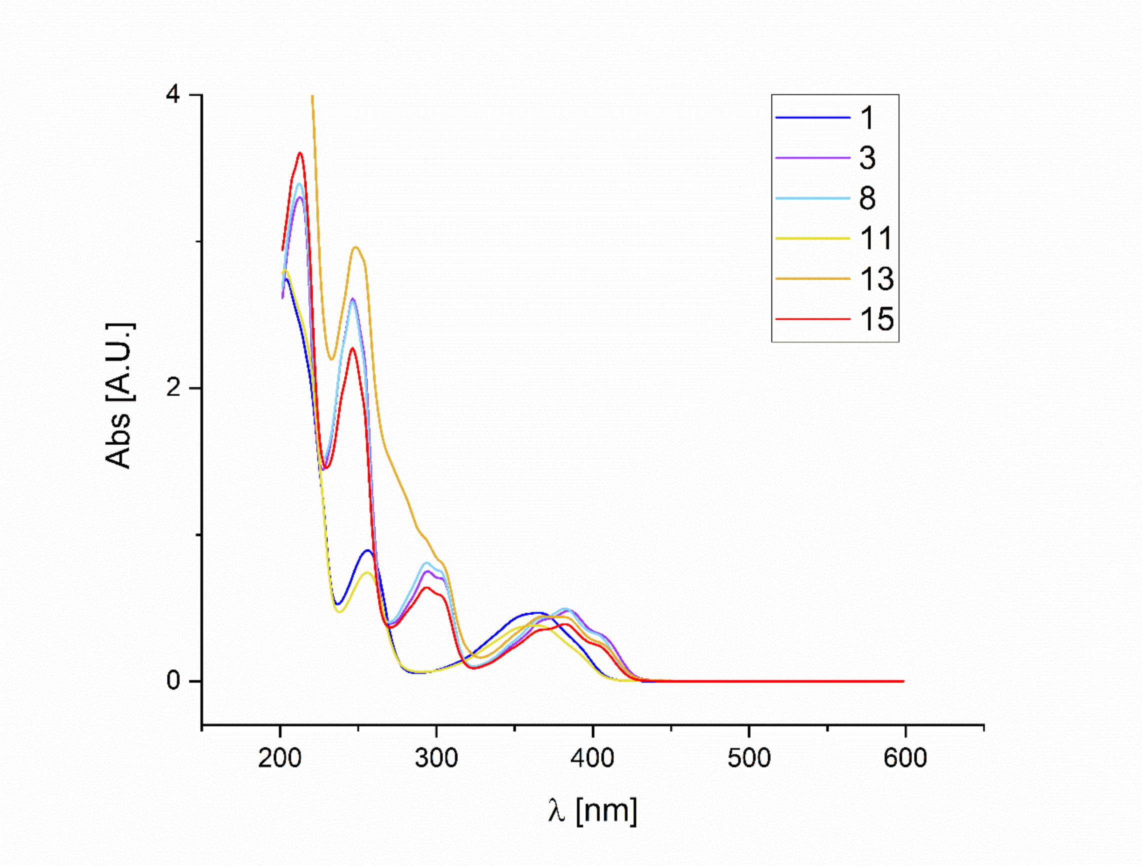

Figure S3: UV-VIS absorption spectra for $1{\cdot}10^{-4}$ mol/dm^3 acetonitrile solutions of compounds 1, 3, 8, 11, 13, and 15

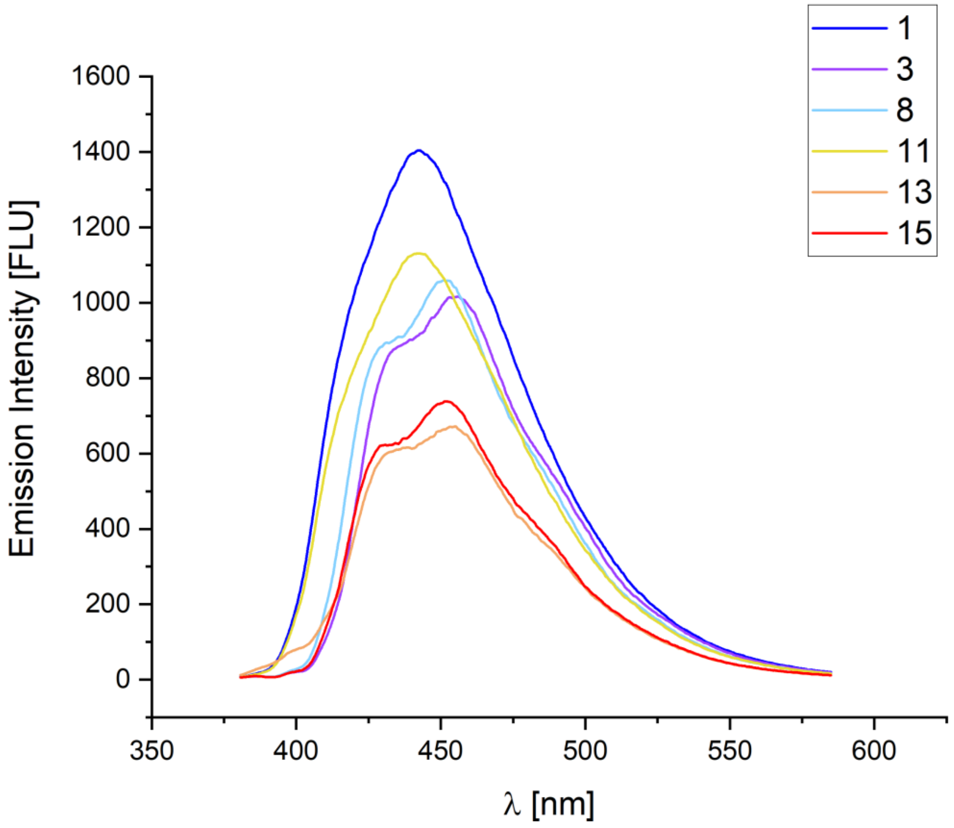

Figure S4: Non-normalized UV-VIS emissionn spectra, recorded at 365 nm excitation wavelength (5 nm excitation slit, 2.5 nm emission slit, 700V PMT voltage) for $1{\cdot}10^{-6}$ mol/dm^3 acetonitrile solutions of compounds 1, 3, 8, 11, 13 and 15

4. Stability of Safirinium alkyne probe **1**: fluorescent RP-HPLC results

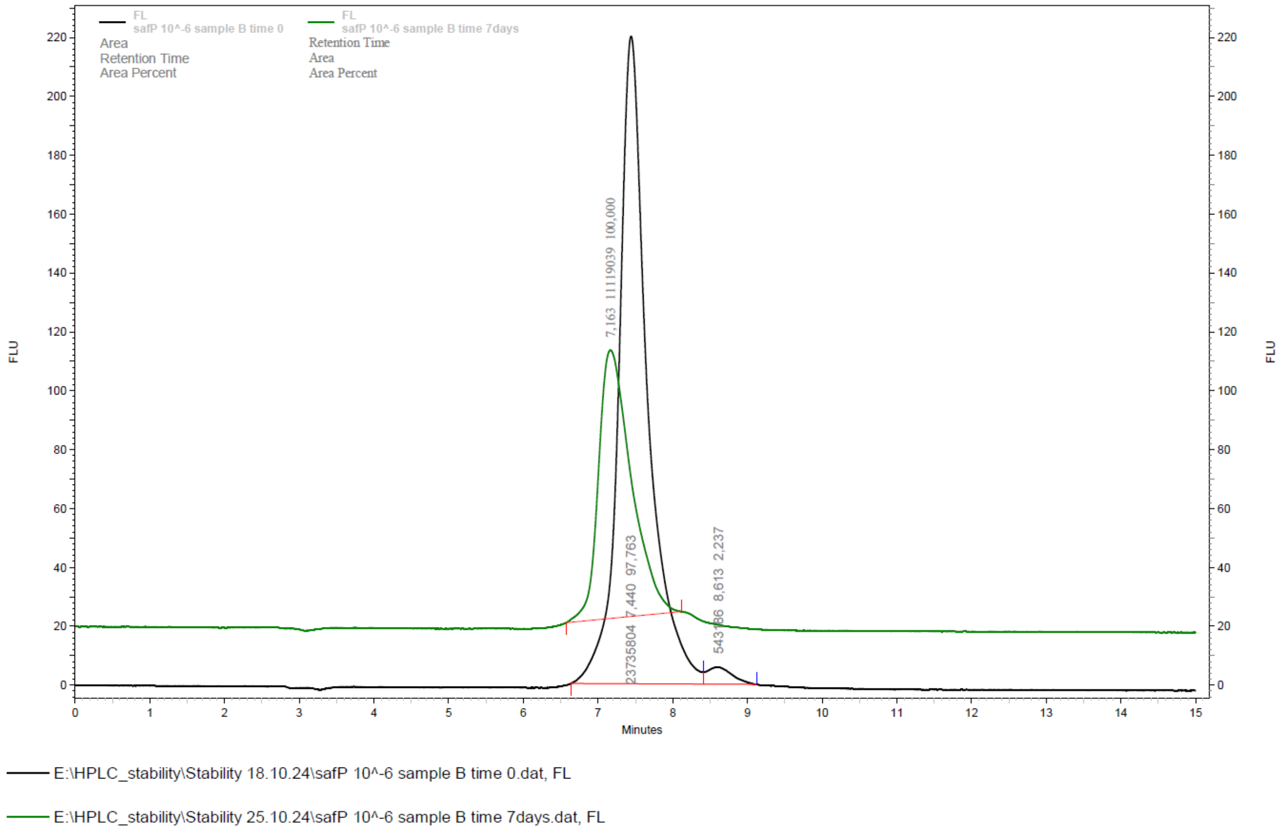

Figure S5: RP-HPLC-Fluorescence chromatograms recorded for the initial stability study of alkyne probe 1.
